# Exploring the effect of Yinzhihuang granules on alcoholic liver disease based on pharmacodynamics, network pharmacology and molecular docking

**DOI:** 10.1186/s13020-023-00759-z

**Published:** 2023-05-11

**Authors:** Yingying Tan, Fanqin Zhang, Xiaotian Fan, Shan Lu, Yingying Liu, Zhishan Wu, Zhihong Huang, Chao Wu, Guoliang Cheng, Bing Li, Jiaqi Huang, Antony Stalin, Wei Zhou, Jiarui Wu

**Affiliations:** 1 Department of Clinical Chinese Pharmacy, School of Chinese Materia Medica, Beijing University of Chinese Medicine, Beijing, China; 2 State Key Laboratory of Generic Manufacture Technology of Chinese Traditional Medicine, Linyi, China; 3 Institute of Fundamental and Frontier Sciences, University of Electronic Science and Technology of China, Chengdu, China; 4 Department of Pharmacy, China-Japan Friendship Hospital, Beijing, 100029 China

**Keywords:** Yinzhihuang granules, Alcoholic liver disease, Network pharmacology, Molecular docking

## Abstract

**Background:**

Yinzhihuang granules (YZHG) is a commonly used Chinese patent medicine for the treatment of liver disease. However, the mechanism of YZHG in alcoholic liver disease (ALD) is still unclear.

**Methods:**

This study combined liquid chromatography-mass spectrometry technology, pharmacodynamics, network pharmacology and molecular docking methods to evaluate the potential mechanism of YZHG in the treatment of ALD.

**Results:**

A total of 25 compounds including 4-hydroxyacetophenone, scoparone, geniposide, quercetin, baicalin, baicalein, chlorogenic acid and caffeic acid in YZHG were identified by ultra performance liquid chromatography tandem mass spectrometry (UPLC-MS/MS). The pharmacodynamic investigations indicated that YZHG could improve liver function and the degree of liver tissue lesions, and reduce liver inflammation and oxidative stress in ALD mice. Network pharmacology analysis showed that YZHG treated ALD mainly by regulating inflammation-related signaling pathways such as the PI3K-Akt signaling pathway. The results of the PPI network and molecular docking showed that the targets of SRC, HSP90AA1, STAT3, EGFR and AKT1 could be the key targets of YZHG in the treatment of ALD.

**Conclusion:**

This study explored the potential compounds, potential targets and signaling pathways of YZHG in the treatment of ALD, which is helpful to clarify the efficacy and mechanism of YZHG and provide new insights for the clinical application of YZHG.

**Supplementary Information:**

The online version contains supplementary material available at 10.1186/s13020-023-00759-z.

## Introduction

Alcoholic liver disease (ALD) is one of the most common chronic liver diseases in the world caused by long-term excessive alcohol consumption [1]. It can progress and lead to liver fibrosis, cirrhosis, and even liver cancer, which seriously endangers people's health [2–4]. The pathogenesis of ALD is complex and diverse, and many factors interact with each other. The main influencing factors are oxidative stress related to alcohol metabolism, inflammatory mediators, endotoxin in the gut etc., [5–8]. At present, there is no specific drug for the clinical treatment of ALD [9, 10]. Western medicine can improve liver injury to some extent, but it is easy to tolerate and the clinical effect is not significant [11, 12]. Traditional Chinese medicine has a long history in the treatment of ALD and has accumulated a lot of valuable experience and characteristic drugs, which are a great treasure for drug development [13–15].

**Fig. 1:**
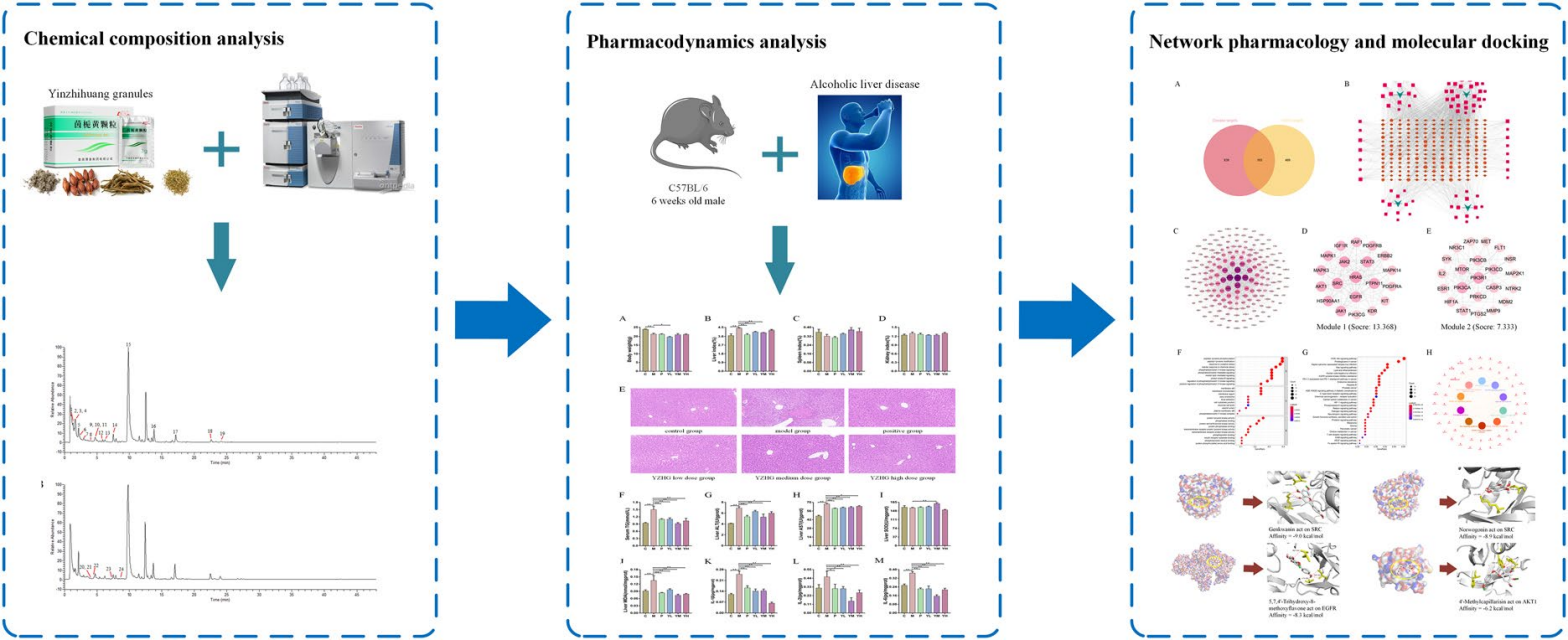
The workflow of YZHG in treating ALD. Firstly, UPLC-MS/MS method was used to identify the chemical composition of YZHG, and then the pharmacodynamic study of YZHG in the treatment of ALD was conducted. Finally, the potential mechanism of YZHG in the treatment of ALD was explored by network pharmacology, and was preliminary verified by molecular docking

Yinzhihuang granules (YZHG) is obtained from “Yinchenhao Decoction”, which consists of four herbs extracts: *Artemisia capillaris* Thunb. (Artemisiae scopariae herba, Yinchen), *Gardenia jasminoides* J.Ellis (Gardeniae fructus, Zhizi), *Scutellaria baicalensis* Georgi (Scutellariae radix, Huangqin) and *Lonicera japonica* Thunb. (Lonicerae japonicae flos, Jinyinhua). YZHG can repair liver cells, improve liver function, and alleviate inflammatory reactions [16, 17]. It is a common Chinese patent medicine for the treatment of liver disease in the clinic [18]. In previous clinical studies, compared to the treatment of ALD with western drugs alone, YZHG combined with western drugs can significantly increase the clinical total effective rate, reduce the incidence of clinical adverse reactions, and enhance the liver protection effect of western drugs, which is worth clinical promotion [19, 20]. However, the clinical and experimental studies on the treatment of ALD with YZHG alone have not been reported. The chemical composition of YZHG is rarely studied, and its pharmacodynamic material basis is still unclear. In addition, the pharmacodynamic investigations of YZHG in the treatment of ALD has not been reported, and its mechanism needs to be elucidated. Therefore, this study investigated the potential active components, targets, and signaling pathways of YZHG in the treatment of ALD by UPLC-MS/MS, network pharmacology and molecular docking. Moreover, a mice model of ALD was established to further verify the efficacy of YZHG in the treatment of ALD. This study provides scientific basis for further clinical research and application of YZHG in the treatment of ALD. The workflow of this study is shown in Fig. 1.

## Materials and methods

### Drugs and reagents

YZHG was provided from the Lunan Houpu Pharmaceutical Co., Ltd. (Shandong, China). The methanol, formic acid, and acetonitrile (mass spectrometry grade) were purchased from Thermo Fisher Scientific Co., Ltd (Shanghai, China). Silibinin capsule was purchased from Tianjin Tasly Sants Pharmaceutical Co., Ltd. (Tianjin, China). 56° Hongxing Erwotou was purchased from Beijing Hongxing Co., Ltd. (Beijing, China). The triglyceride (TG), alanine aminotransferase (ALT), aspartate aminotransferase (AST), superoxide dismutase (SOD) and total protein (TP) test kits were purchased from Nanjing Jiancheng Bioengineering Institute (Nanjing, China). The malondialdehyde (MDA) enzyme linked immunosorbent assay (ELISA) kits was purchased from Jiangsu Kete Biotechnology Co., Ltd. (Yancheng, China). The interleukin-1β (IL-1β), interleukin-2 (IL-2), and interleukin-6 (IL-6) ELISA kits were purchased from Beijing Dogesce Boye Biotechnology Co., Ltd. (Beijing, China).

### UPLC-MS/MS

#### Preparation of YZHG solution

YZHG was accurately weighed 2.000 g, dissolved in 10 mL water, ultrasonic extraction for 30 min, and then centrifuged at 6000 rpm/min for 5 min. The obtained supernatant filtered by 0.22 μm micropore filter membrane, and the YZHG solution was obtained.

#### UPLC-MS/MS system and operating conditions

The liquid chromatography-mass spectrometry system consisted of the Thermo Scientific UItimate 3000 ultra-high performance liquid chromatograph and the Thermo Scientific LTQ-Orbitrap XL mass spectrometer equipped with a electrospray ionization (ESI) source. The column was Waters Acquity PRM UPLC BEH C_18_ Column with Van Guard FIT (1.7 μm, 2.1 × 100 mm).

The mobile phase composed of 0.1% (*V/V*) formic acid–water (A) and acetonitrile (B). The flow rate was 0.2 mL/min. The injection volume was 2 μL and the elution procedure was set as follows: 1% (B) in 0–5 min, 1–15% (B) in 5–14 min, 15–35% (B) in 14–28 min, 35–55% (B) in 28–38 min, 55–95% (B) in 38–48 min.

Mass spectrometry conditions were as follows: ESI source, negative and positive ion detection mode; sheath gas volume flow, 40 L/min; auxiliary gas volume flow, 20 L/min; spray voltage, 3.0 kV; ion transmission tube temperature, 350 °C; scanning range *m*/*z* 50–1200; detection resolution, 3000; collision voltage, 6–10 V. Multi-stage fragment ion detection by mass spectrometry was performed by data-dependent scanning, and the fragmentation mode was collision-induced dissociation. All MS spectra were collected using the Xcalibur 2.1 software.

#### Data processing

After the sample solution was analyzed and detected according to the above conditions, the Xcalibur 2.1 software was used to complete data processing, including the extraction of the ion chromatogram and fragmentation behavior information. The mass error of each main fragment ion was within 5 ppm. The measured secondary mass spectrometric fragments were matched with mz Cloud database and self-built traditional Chinese medicine component database from literature. Finally, ion fragment information, accurate molecular mass and comparative inference were used to further validate the compounds.

### Animal experiment

#### Animals

All animal experiments were conducted strictly according to the guidelines and requirements of the animal experiment ethics committee of Beijing University of Chinese Medicine (permit number: BUCM-4-2021091007-3156). Male C57BL/6 mice (18–20 g body weight and 6 weeks old) were purchased from SPF Biotechnology Co., Ltd. (certification: SCXK Beijing, 2016–0002; Beijing, China). All mice were housed in the animal laboratory of Beijing University of Chinese Medicine (temperature 20 ~ 24 ℃, 40% ~ 45% humidity, and a 12 h light–dark cycle), and the mice were free to eat and drink during the rearing period.

#### Experimental animal model and drug interventions

After 1 week of adaptive feeding, mice were randomly divided into 6 groups (*n* = 6), namely control group (**C**), model group (**M**), 43.05 mg/kg of positive group (**P**), 3.69 g/kg of YZHG low dose group (**YL**), 7.38 g/kg of YZHG medium dose group (**YM**) and 14.76 g/kg of YZHG high dose group (**YH**). The doses of the positive drug (silibinin capsule) and YZHG were converted into corresponding doses based on the body surface area of humans and animals. The low, medium and high doses of YZHG correspond to 1, 2, and 4 times the clinical equivalent dose, respectively. The alcoholic liver disease model was replicated according to the reference [21]. Every morning, the **C** group was weighed and gavaged with distilled water, and the other groups were gavaged with 56° Hongxing Erwotou. And 8 h later, the **C** and **M** groups were fed with distilled water and the other groups received appropriate drug feeding. The amount of each gavage was 0.1 mL/10 g, for a total of 2 weeks, during which the mice were fed normally. After the last administration, the mice were fasted for 12 h, weighed and the mice were anesthetized by intraperitoneal injection of chloral hydrate. And then the samples were collected.

#### Determination of organ index

The sacrificed mice were quickly dissected to remove the liver, spleen and kidney, and the fat and fascia were removed. The surface liquid was swabbed with filter paper, and the corresponding organ index was calculated. The calculation formulas: spleen index = spleen weight (g)/body weight (g) × 100%, kidney index = kidney weight (g)/body weight (g) × 100%, liver index = liver weight (g)/body weight (g) × 100%.

#### Evaluation of liver histology

Part of the liver tissue was fixed with 4% paraformaldehyde. The samples were dehydrated step by step in an automatic dehydrator, embedded in paraffin, and sliced, and then stained with hematoxylin–eosin (HE) reagent. After mounting, the pathological changes of the liver were observed under the microscope.

#### Determination of biochemical indicators

After standing at room temperature for 1 h, the blood samples were centrifuged at 4 °C and 3 000 rpm/min for 15 min, and the serum was collected. The liver homogenate was prepared according to the instructions of the quantitative protein assay kit and its protein concentration was determined. The levels of TG and ALT, AST, SOD and MDA were determined according to the corresponding steps in the instructions of the kit instructions.

#### Determination of inflammatory cytokines

The relative expression levels of IL-1β, IL-2 and IL-6 in liver tissue were determined by ELISA kits, and the procedures were performed strictly according to the instructions.

### Network pharmacology

#### Screening of potential active ingredients and targets of YZHG

Traditional Chinese Medicine Systems Pharmacology Database and Analysis Platform (TCMSP, http://lsp.nwu.edu.cn/tcmsp.php) [22, 23] was used to collect the potential active ingredients of YZHG by using “Artemisiae scopariae herba”, “Gardeniae fructus”, “Scutellariae radix” and “Lonicerae japonicae flos” as keywords with the screening conditions of oral bioavailability (OB) ≥ 30% and drug-likeness (DL) ≥ 0.18 [24, 25]. Retrieved on November 19, 2021. At the same time, combined with UPLC-MS/MS identification results and the literature [18], the potential active ingredients of YZHG were improved and integrated. The SMILES information of active ingredients was collected from the PubChem database (https://pubchem.ncbi.nlm.nih.gov/) [26], and the SMILES information was imported into the SWISS TargetPrediction database (http://www.swisstargetprediction.ch/) [27] to predict the potential targets of YZHG. Retrieved on November 19, 2021.

#### Screening of ALD targets

The GeneCards (https://www.genecards.org/) [28], DisGeNET (https://www.disgenet.org/) [29] and OMIM (https://omim.org/) [30] databases were used to search for disease targets using the keyword “alcoholic liver disease”. Retrieved on February 10, 2022. There are too many ALD targets in the GeneCards database, and a relevance score greater than 20 was set as the screening condition [31]. The drug targets and disease targets were mapped online by Jvenn (http://www.bioinformatics.com.cn) [32], and the common targets were obtained as potential targets of YZHG in the treatment of ALD. The herbs, active ingredients, and common targets of YZHG were imported into Cytoscape 3.7.2 software, and the herbs-active ingredients-targets network of YZHG in the treatment of ALD was constructed.

#### Protein–protein interaction (PPI) network construction and module analysis

The STRING 11.5 database (https://cn.string-db.org/) [33] was used to analyze common targets, setting “Homo sapiens” as the organism and a confidence score  ≥ 0.7. Cytoscape 3.7.2 software was then used to visualize the results and construct a PPI network. The PPI network was analyzed by the MCODE plug-in in Cytoscape 3.7.2 software, and the k-Core value was set to 5 to screen key protein modules [34].

#### Gene ontology (GO) analysis and Kyoto encyclopedia of genes and genomes (KEGG) enrichment analysis

R 3.6.1 software was used to analyze common targets with greater than average degree values in PPI networks. GO and KEGG enrichment analysis were also performed [35], and the results were presented in the form of bubble charts.

### Molecular docking

Molecular docking can be used to evaluate the tightness of binding between the active ingredient and the core target. Molecular docking was performed after screening key targets and the key active ingredients from the PPI network and the “herbs-active ingredients-targets” network of YZHG in treatment of ALD. The 3D crystal structure of the core protein gene was downloaded from the RCSB PDB database (http://www1.rcsb.org/) [36] and imported into PyMol 1.7.4.5 to isolate the original ligand and receptor, and then the target protein was obtained. The mol2 format files of key ingredients were downloaded from the TCMSP database. AutoDock Tools 1.5.6 and AutoDock Vina 1.1.2 were used for molecular docking, and PyMol 1.7.4.5 software was used to visualize the molecular docking results [37].

### Statistical analysis

SPSS 20.0 software was used for statistical analysis of experimental data, and the results were expressed as mean ± SEM. One-way analysis of variance and Fisher's least significant difference methods were used to analyze the differences between groups, and *P* < 0.05 was considered statistically significant. GraphPad Prism 8.0 software was used to visualize the statistical results.

## Results

### Chemical composition analysis of YZHG based on UPLC-MS/MS

**Fig. 2:**
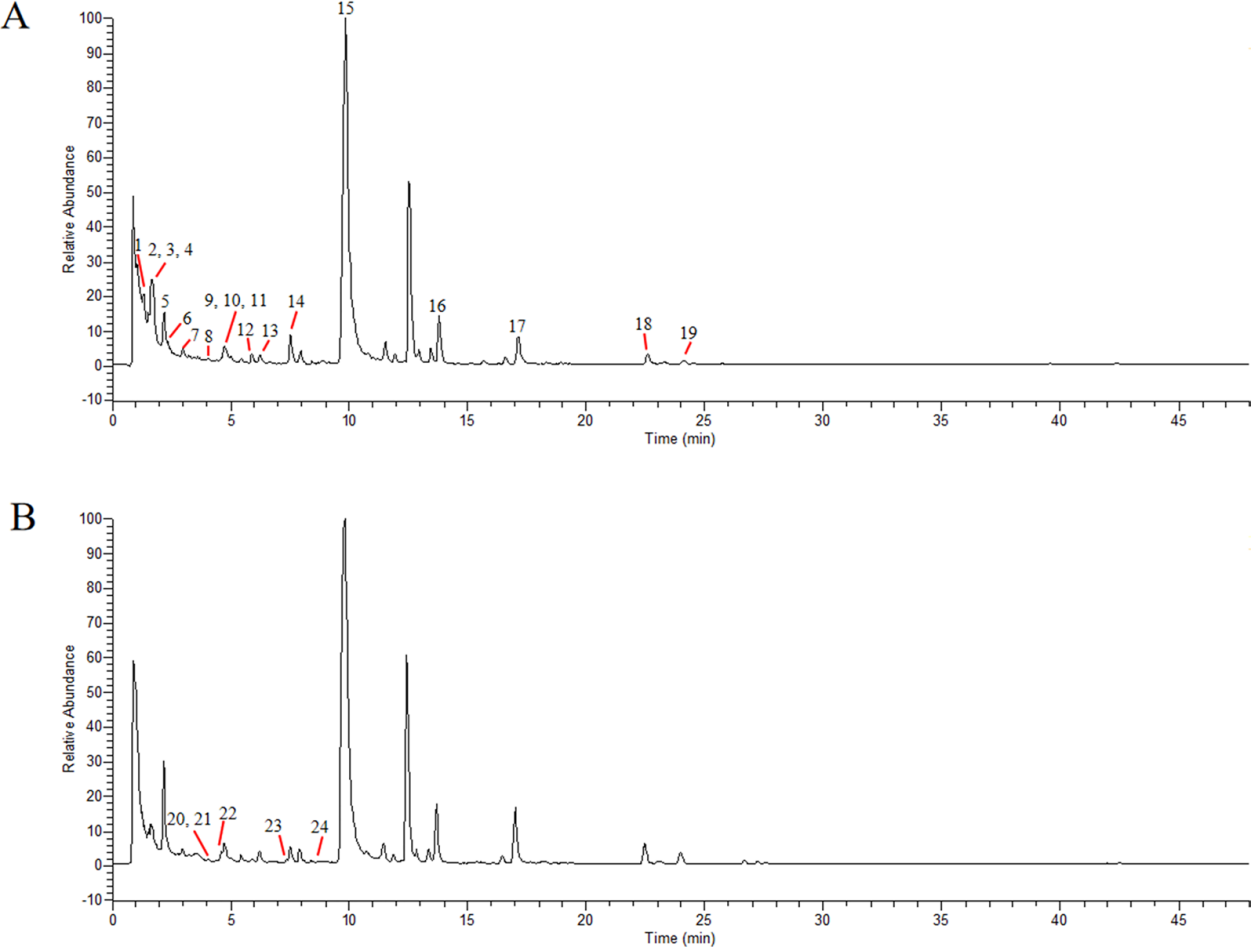
The total ion chromatograms of YZHG solution in negative (**A**) and positive (**B**) ion mode

25 compounds were identified from YZHG solution based on UPLC-MS/MS. The chemical composition of YZHG solution was determined preliminarily, which can be divided into three categories: flavonoids (luteolin, cynaroside, quercetin, rutin, hyperoside, scutellarin, baicalin, wogonoside, baicalein, wogonin, oroxylin A and scutellarein), organic acids (neochlorogenic acid, chlorogenic acid, cryptochlorogenic acid, caffeic acid, ferulic acid, 1,3-dicaffeoylquinic acid, 3,4-dicaffeoylquinic acid, 3,5-dicaffeoylquinic acid and 4,5-dicaffeoylquinic acid), iridoids (geniposide and deacetylasperulosidic acid methyl ester) and others (scoparone, 4-hydroxyacetophenone). The total ion chromatograms of YZHG solution in the positive and negative ion mode is shown in Fig. 2.

### Pharmacodynamics analysis of YZHG in the treatment of ALD

#### Effects of YZHG on body weight and organ index

**Fig. 3:**
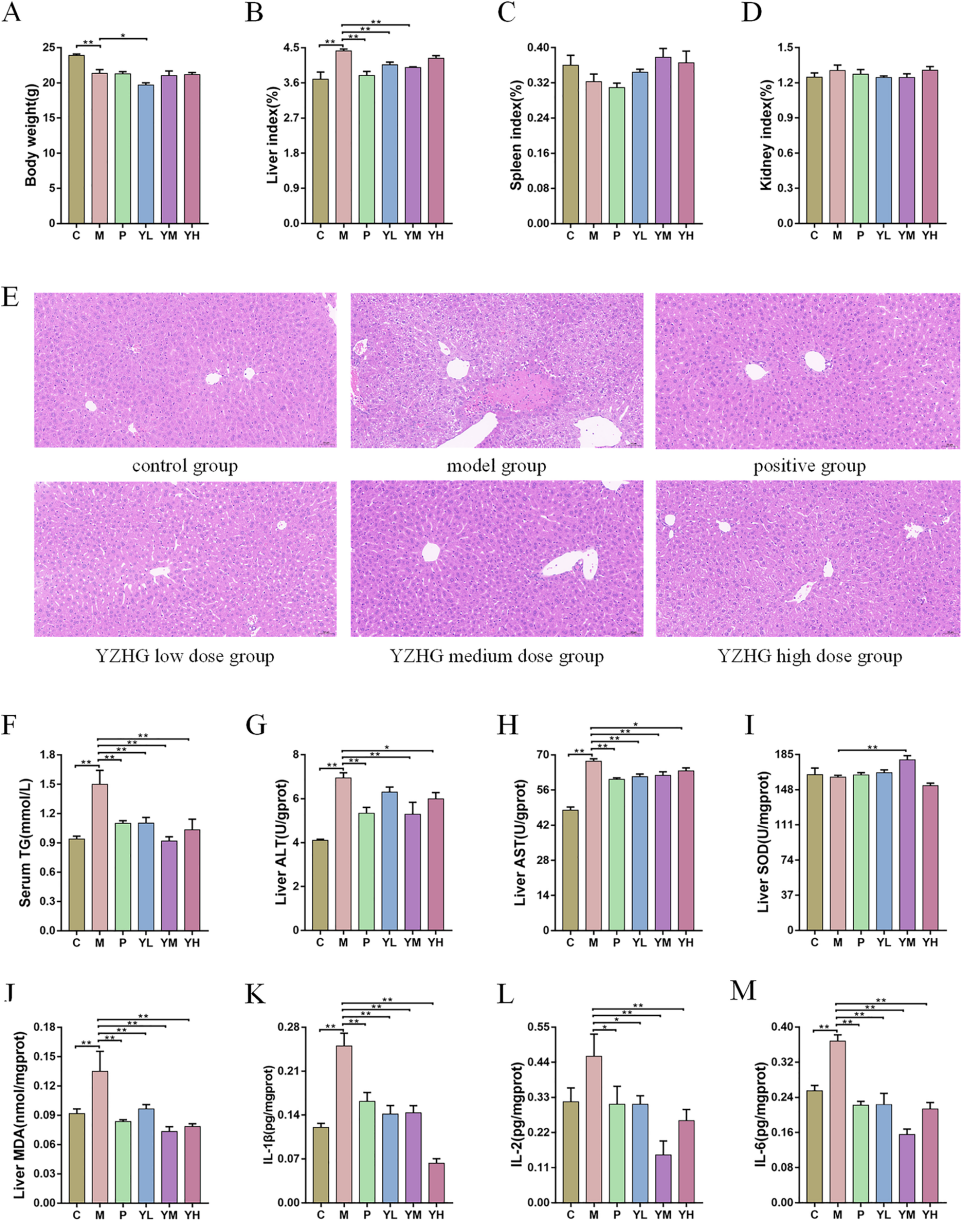
Pharmacodynamic analysis of YZHG against ALD. **A** Body weight. **B** Liver index. **C** Spleen index. **D** Kidney index. **E** HE staining results of liver tissues in each group (Bar = 50 μm). **F** Serum TG. **G** liver ALT. **H** liver AST. **I** liver SOD. **J** Liver MDA. **K** IL-1β. **L** IL-2. **M** IL-6. Data are presented as the mean ± SEM in each group. ^*^P < 0.05 and ^**^P < 0.01 compared to the model group

As shown in Fig. 3A, the bodyweight of mice in the **M** group decreased significantly compared with the **C** group (*P* < 0.01); compared with the M group, the other three groups, except for the **YL** group, did not show significant changes. Compared with **C** group, the liver index of mice in the **M** group was significantly increased (*P* < 0.01); compared with the **M** group, except for the **YH** group, the liver indexes of the other four groups were decreased (Fig. 3B). The spleen index and kidney index of the mice were not significantly different from the **M** group, and there was no statistical significance (Fig. 3C, D). This indicated that YZHG can improve alcohol-induced liver hypertrophy.

#### Effects of YZHG on liver histopathology

As shown in Fig. 3E, the liver tissue of the **C** group showed normal liver structure. In contrast, the liver tissue of the **M** group showed obvious and significant histopathological changes, such as a larger amount of hepatocyte granular degeneration, localized focal necrosis of hepatocytes, and punctate infiltration of neutrophils. Compared with **M** group, the administration group had different degrees of alleviation, suggesting that YZHG could alleviate alcohol-induced pathological liver damage.

#### Effects of YZHG on biochemical indicators

AS shown in Fig. 3F, compared with the **C** group, the level of TG in the **M** group were significantly increased, and the difference was statistically significant (*P* < 0.01). Compared with the **M** group, the level of serum TG was significantly decreased in the administration group (*P* < 0.01).The effects of YZHG on the liver biochemical indexes of ALD mice are shown in Fig. 3G–J. Compared with the **M** group, the ALT of **C**, **P**, **YM** and **YH** groups decreased significantly, and the AST and MDA of **C** group and the four administration groups decreased significantly (*P* < 0.05). In addition, the SOD level was significantly increased in the **YM** group compared with the **M** group (*P* < 0.01). This indicated that YZHG can reduce fat accumulation in the liver, alleviate alcohol-induced liver damage, and have a protective effect on liver oxidative damage.

#### Effects of YZHG on inflammatory cytokines

As shown in Fig. 3K–M, IL-1β and IL-6 were significantly increased in the **M** group compared with the **C** group (*P* < 0.01), indicating that alcohol can cause inflammatory liver damage. Compared with the **M** group, IL-1β, IL-2 and IL-6 were significantly decreased in the administration group (*P* < 0.05). It is worth noting that the effect of reducing IL-1β in the **YH** group was better than that of the **P** group, and the effect of reducing IL-2 and IL-6 in the **YM** group was better than that of the **P** group. These results suggested that YZHG can improve the liver inflammation caused by alcohol to protect the liver.

### Network pharmacology

#### Herbs-active ingredients-targets network of YZHG in the treatment of ALD

**Fig. 4:**
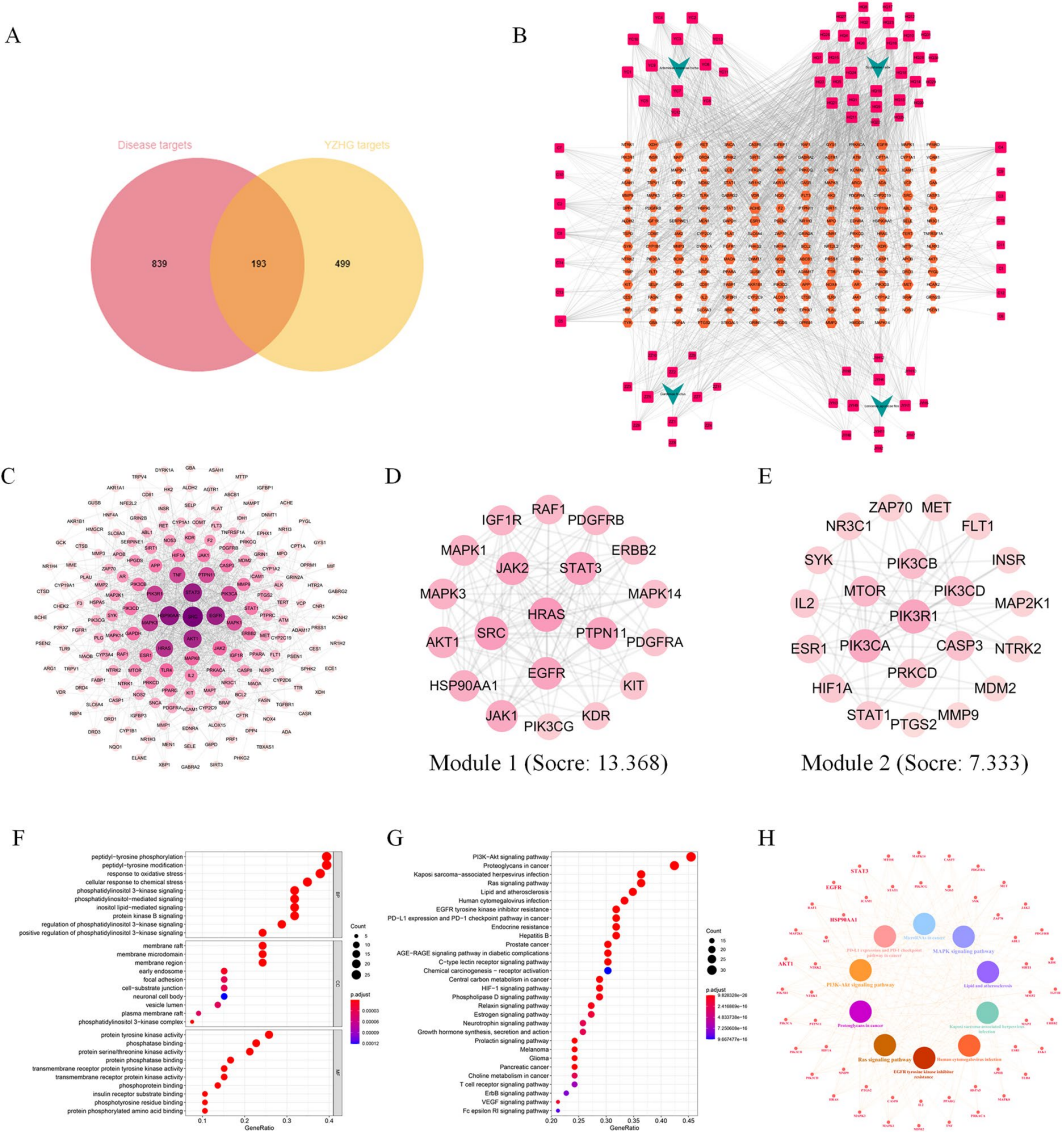
Network pharmacological analysis. **A** Venn diagram of the common targets of YZHG and ALD. **B** The herbs-active ingredients-targets network of YZHG in the treatment of ALD. The green arrow node represents herb, the red square node represents active ingredient, and the orange hexagon node represents target. The larger the node area is, the more important the node is in the network. The edge represents the interrelationship between herbs, active ingredients, and targets. **C** The PPI network of common targets. The darker the node color is, the larger the node area is, and the more important the node is in the PPI network. **D** Module 1 of module analysis. **E** Module 2 of module analysis. **F** Bubble diagram showing the results of GO enrichment analysis, including top 10 terms in BP, MF, and CC, respectively. **G** Top 30 pathways of KEGG enrichment analysis. **H** The network showing the detailed genes involved in the top 10 pathways and the key targets were enlarged. The nodes of innermost circle represent the pathways, and the nodes of outer two circles represent the targets

Combined with the results from UPLC-MS/MS, literature and TCMSP database, a total of 82 active ingredients of YZHG were obtained (Additional file 1: Table S1). The target predictions of the potential active ingredients were predicted by the Swiss TargetPrediction database, and 692 targets were obtained after deduplication. The GeneCards, DisGeNET, and OMIM databases were searched, and after deduplication, a total of 1032 target genes were found to be associated with ALD. Jvenn online mapping identified 193 common targets (Fig. 4A) that were potential targets of YZHG in the treatment of ALD. And the herbs-active ingredients-targets network of YZHG in the treatment of ALD was constructed, as shown in Fig. 4B. In this network, the top 8 active ingredients in degree value are moslosooflavone, 4'-methylcapillarisin, norwogonin, 5,7,4'-trihydroxy-8-methoxyflavone, 5,7,2,5-tetrahydroxy-8,6-dimethoxyflavone, genkwanin, 5,2'-dihydroxy-6,7,8-trimethoxyflavone, and 3-methylkempferol. These ingredients may be the key active ingredients of YZHG in the treatment of ALD.

#### PPI network and module analysis

The PPI network of common targets of YZHG and ALD is shown in Fig. 4C. The network consisted of a total of 186 nodes and 1194 edges. From the inner circle to the outer circle, the degree value decreases. According to the degree value in the PPI network, the five targets SRC, HSP90AA1, STAT3, EGFR and AKT1 could be the key targets of YZHG in the treatment of ALD. The MCODE plug-in in Cytoscape 3.7.2 software was used for module analysis, and a total of 2 protein modules were obtained, as shown in Fig. 4D, E.

#### KEGG and GO analysis

**Fig. 5:**
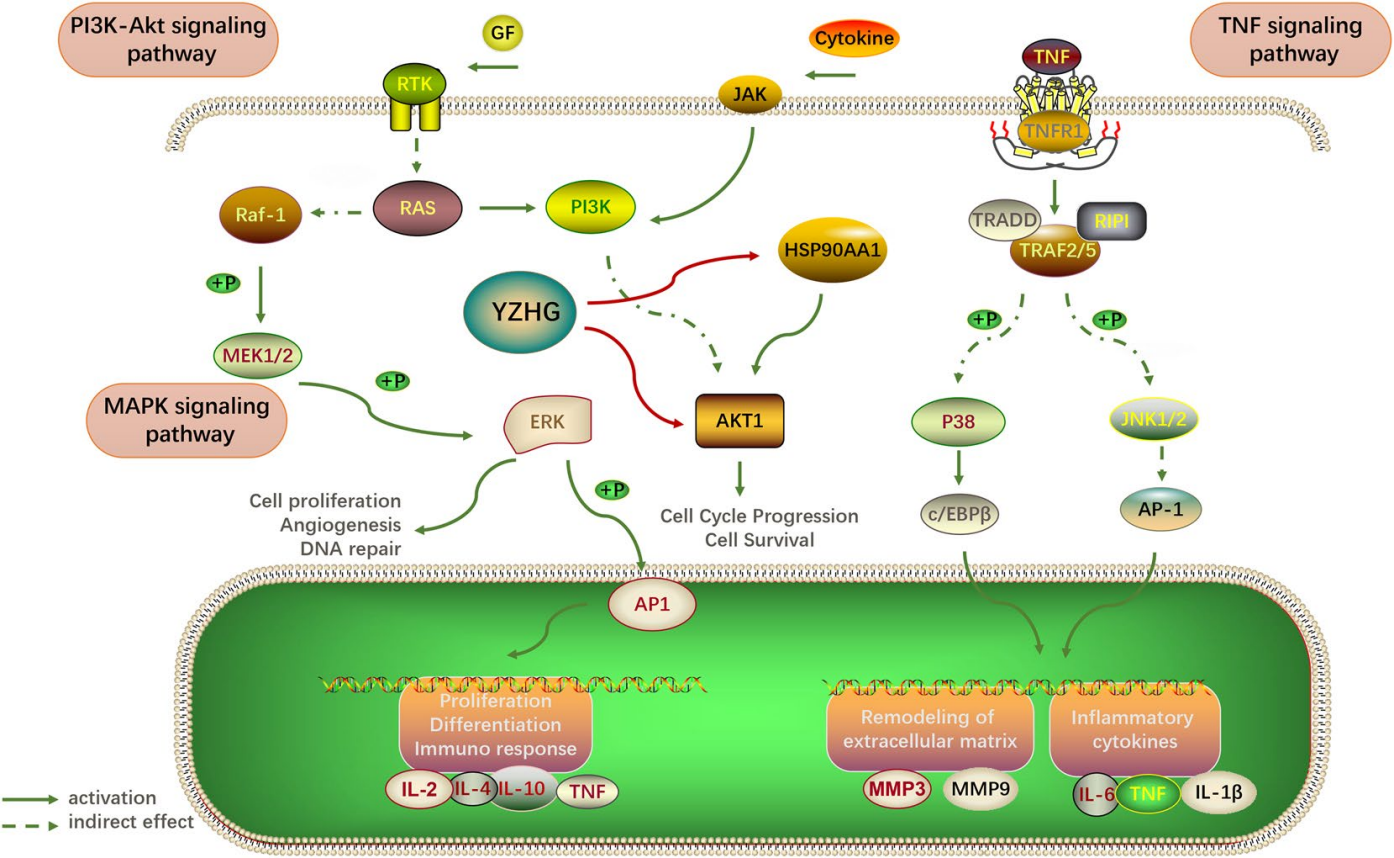
Illustration of the crucial biological processes involving potential targets of YZHG in the treatment of ALD

R software was used to perform GO and KEGG enrichment analysis for the 67 key targets with greater than average degree value in the PPI network. The results showed that these targets were enriched in 2125 biological processes (BP), 60 molecular functions (MF), 113 cellular components (CC) (Additional file 1: Table S2) and 228 KEGG pathways (*P* < 0.05) (Additional file 1: Table S3). In addition, the BP of ALD were mainly related to oxidative stress, such as “response to oxidative stress”, “cellular response to chemical stress”, “metabolic process of reactive oxygen species” and “cellular response to oxidative stress”. The MF might be related to “protein tyrosine kinase activity”, “phosphatase binding” and “signaling receptor activity”. The CC were principally associated with “membrane raft”, “membrane microdomain” and “membrane region” (Fig. 4F). KEGG pathway analysis also showed that these targets were significantly enriched in pathways related to inflammation and lipid metabolism, including “PI3K-Akt signaling pathway”, “proteoglycans in cancer”, “Ras signaling pathway”and “lipid and atherosclerosis” (Fig. 4G). In addition, the detailed network of KEGG signaling pathways revealed that many inflammation-related target genes were involved in the progression of ALD (Fig. 4H). In the animal experiments of this study, YZHG can significantly reduce the levels of IL-1β, IL-2 and IL-6 in the liver of ALD mice, presumably by affecting the PI3K-Akt signaling pathway, MAPK signaling pathway and other signaling pathways to influence the secretion of inflammatory cytokines, thereby improving the inflammatory response of the liver (Fig. 5).

### Molecular docking verification

**Fig. 6:**
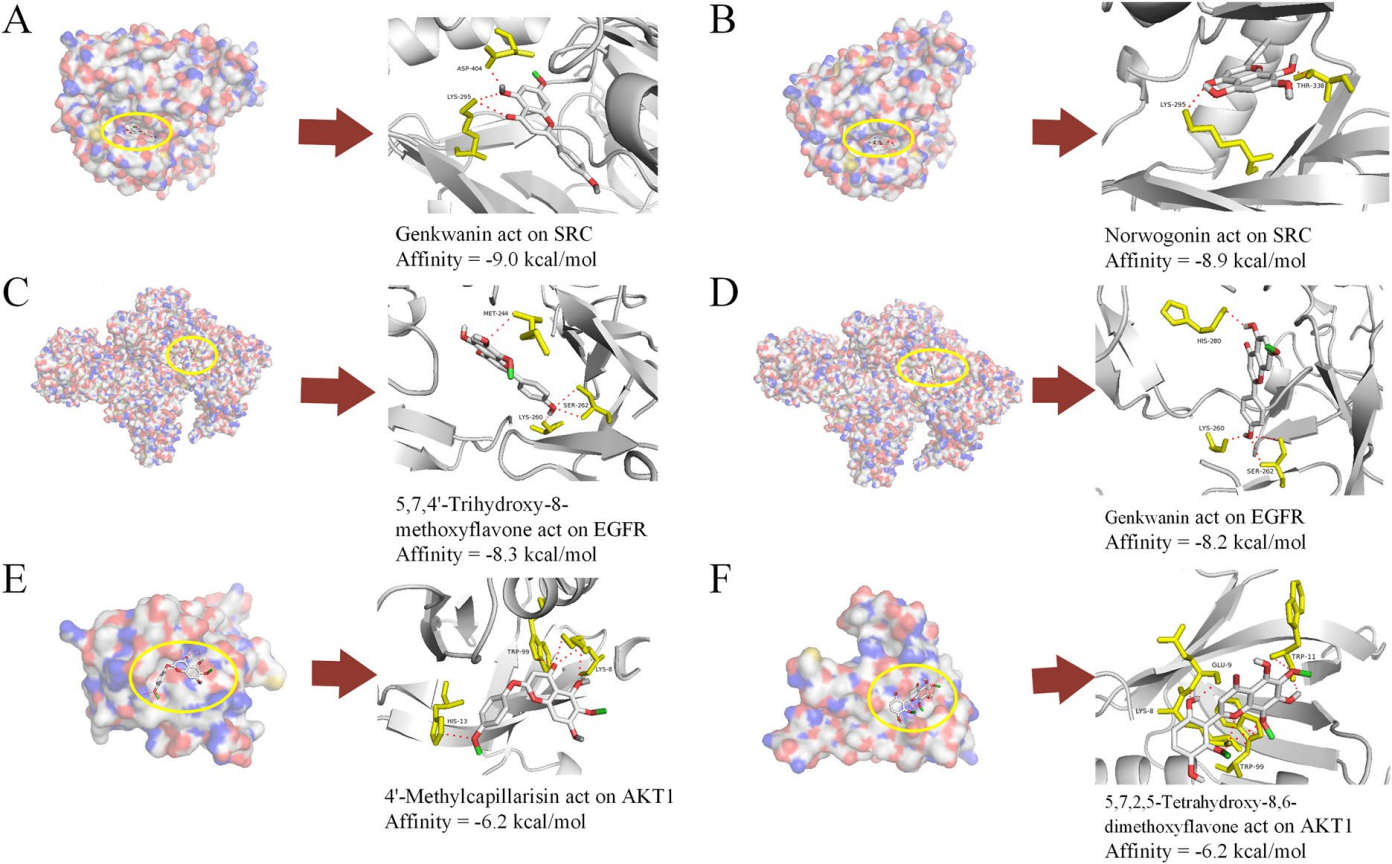
Visualization of molecular docking results. **A** Genkwanin formed three hydrogen bonds with residues of ASP-404 and LYS-295 on SRC protein. **B** Norwogonin formed two hydrogen bonds with residues of THR-338 and LYS-295 on SRC protein. **C** 5,7,4'-Trihydroxy-8-methoxyflavone formed four hydrogen bonds with residues of MET-244, SER-262 and LYS-260 on EGFR protein. **D** Genkwanin formed four hydrogen bonds with residues of HIS-280, SER-262 and LYS-260 on EGFR protein. **E** 4'-Methylcapillarisin formed five hydrogen bonds with residues of HIS-13, TRP-99 and LYS-8 on AKT1 protein. **F** 5,7,2,5-Tetrahydroxy-8,6-dimethoxyflavone formed eight hydrogen bonds with residues of GLU-9, TRP-99, TRP-11 and LYS-8 on AKT1 protein

In order to further explore the mechanism of YZHG in the treatment of ALD, the key targets and the corresponding key active compounds were selected for molecular docking, as shown in Table 1. PyMol was used to visualize some of the results, as shown in Fig. 6. The results showed that the binding energy between the active ingredients and the targets was less than −5.0 kcal/mol [24], indicating that the ingredients and the targets were tightly bound, and the active compounds could act on the key targets to achieve the therapeutic effect.

**Table 1:** Molecular docking results

NO	Proteins	PDB ID	Test compounds	Affinity (kcal/mol)
1	SRC	2H8H	Genkwanin	−9.0
2	SRC	2H8H	Norwogonin	−8.9
3	SRC	2H8H	Moslosooflavone	−8.6
4	SRC	2H8H	5,7,4'-Trihydroxy-8-methoxyflavone	−8.6
5	SRC	2H8H	3-Methylkempferol	−8.6
6	SRC	2H8H	5,2'-Dihydroxy-6,7,8-trimethoxyflavone	−8.4
7	EGFR	5WB7	5,7,4'-Trihydroxy-8-methoxyflavone	−8.3
8	EGFR	5WB7	Genkwanin	−8.2
9	EGFR	5WB7	Norwogonin	−8.0
10	SRC	2H8H	5,7,2,5-Tetrahydroxy-8,6-dimethoxyflavone	−7.8
11	EGFR	5WB7	Moslosooflavone	−7.8
12	EGFR	5WB7	4'-Methylcapillarisin	−7.8
13	EGFR	5WB7	5,7,2,5-Tetrahydroxy-8,6-dimethoxyflavone	−7.5
14	EGFR	5WB7	3-Methylkempferol	−7.5
15	EGFR	5WB7	5,2'-Dihydroxy-6,7,8-trimethoxyflavone	−7.1
16	AKT1	1UNQ	4'-Methylcapillarisin	−6.2
17	AKT1	1UNQ	5,7,2,5-Tetrahydroxy-8,6-dimethoxyflavone	−6.2
18	AKT1	1UNQ	3-Methylkempferol	−5.8

## Discussion

YZHG are derived from the classical prescription “Yinchenhao Decoction” for the treatment of liver disease. Modern pharmacological studies have shown that Yinzhihuang can reduce enzymes, protect liver, promote liver cell regeneration and antibacterial [38–40]. Long term heavy drinking can lead to alcoholic liver damage, and ethanol and its metabolites consume a large amount of antioxidant factors in the body [6]. When a large amount of oxides accumulate in the body and liver cells cannot be cleared in a timely manner, it can lead to lipid peroxidation reactions in the liver cell membrane, causing liver cell inflammation, fatty degeneration, inflammatory cell infiltration, and even liver cell necrosis [41]. When liver cells were damaged, AST and ALT levels in the body will increase [42]. This study showed that YZHG can reduce AST and ALT levels in ALD mice, indicating that YZHG has a certain protective effect on liver damage. In addition, oxidative stress is one of the important pathogenesis of ALD [43]. As the main end product of lipid peroxidation, the content of MDA can directly reflect the degree of lipid peroxidation damage in the body [44]. This study found that YZHG can significantly reduce the content of MDA in liver, suggesting that YZHG can have a protective effect on hepatocyte injury in ALD by enhancing the anti-lipid peroxidation ability in vivo. In addition, clinical studies have shown that Yinzhihuang can significantly reduce the inflammatory response of patients, improve the liver enzyme indexes of patients, and can be used clinically for the treatment of ALD; this is consistent with the results of this animal experimental study. Silibinin has the function of protecting the liver enzyme system, scavenging free radicals and resisting lipid peroxidation [45, 46]. Clinically, the combination of silibinin and YZHG in the treatment of ALD can improve liver function and liver fibrosis, and reduce the incidence of adverse reactions, which is worthy of clinical recommendation [19, 20].

A total of 25 compounds in YZHG were identified by UPLC-MS/MS, and most of them had good liver protection effect. Geniposide and 4-hydroxyacetophenone have direct anti-liver injury effects, while chlorogenic acid may play an indirect role [47]. Wogonoside can reduce liver fibrosis by triggering hepatic stellate cell iron death through SOCS1/P53/SLC7A11 pathway [48], and has a protective effect on lipopolysaccharide/D-galactosamine-induced acute liver injury in mice [49]. Scoparone has a wide range of pharmacological activities including anti-inflammatory, antioxidant, antiapoptotic, antifibrotic, and lipid-lowering properties and is expected to be a potential candidate for various liver diseases, such as acute liver injury, alcoholic liver disease, and fibrosis [50]. Quercetin attenuates chronic ethanol-induced liver mitochondrial injury by enhancing mitophagy [51]. Luteolin attenuates chronic and alcohol-induced alcoholic liver disease in mice [52]. Caffeic acid protects against oxidative damage by interfering with PDIA3-dependent activation of NADPH oxidase, thereby alleviating liver injury in rats after transplantation [53]. The treatment of diseases with traditional Chinese medicine is a complex process that involves the interaction between multiple components. In this study, it is necessary to further study the transformation and interaction process of YZHG active ingredients in vivo.

In this study, network pharmacology was used to predict the potential active compounds and targets of YZHG for ALD treatment, and molecular docking technology was used to verify the binding ability of key compounds between key targets. The PPI network showed that the degree values of SRC, HSP90AA1, STAT3, EGFR and AKT1 were higher, indicating that the above targets may play an important role in the treatment of ALD. And the molecular docking technology was used to verify, the results show that the key components and key targets have good binding ability. Non-receptor tyrosine kinase (SRC) can alleviate liver fibrosis by inhibiting hepatic stellate cell activation and reducing connective tissue growth factor [54]. In addition, SRC inhibitors can treat liver fibrosis and liver cancer. Studies have shown that drugs can exert anti-inflammatory effects by inhibiting SRC phosphorylation [55]. Inhibition of heat shock protein 90 alpha family class A member 1 (HSP90AA1) can decrease NLRP3 inflammasome activity of macrophages and reduce secretion of cytokines IL-1β and IL-18 in ALD [56]. Signal transducer and activator of transcription 3 (STAT3) is activated by phosphorylation in response to various cytokines and growth factors and is an important transcription factor involved in immune responses and inflammation [57]. Studies have shown that drugs can reduce the levels of IL-6 and IL-1β by inhibiting STAT3 expression, thereby ameliorating liver inflammation [58]; targeting the STAT3 signaling pathway is considered an attractive therapeutic strategy for the development of anti-inflammatory drugs [59, 60]. Epidermal growth factor receptor (EGFR) plays a key role in hepatocyte proliferation and liver regeneration. It is also involved in hepatocyte injury, oxidative stress, inflammation, and fibrosis in models of chronic liver injury, and has also been implicated in hepatocellular carcinogenesis [61]. It also is considered a promising target for the treatment of ALD [62]. AKT serine/threonine kinase 1 (AKT1) can regulate fibrogenesis and proliferation of hepatocytes and hepatic stellate cells and plays a key role in the development of ALD-related hepatic inflammation [63]. These key targets play an important role in the process of inflammatory response and the experimental results of this study showed that YZHG can reduce liver inflammation in ALD mice, presumably through these key targets to exert an anti-inflammatory effect. In order to better reveal the role of these key targets in ALD, in-depth research is needed through cell experiments and animal experiments.

Many studies have shown that liver inflammation plays a key role in the pathogenesis of ALD [64]. Alcohol consumption often causes inflammation in the liver and body, which further contributes to ALD [65]. The inflammatory response is mainly mediated by the NF-kappa B signaling pathway, MAPK signaling pathway, JAK-STAT signaling pathway, PI3K-Akt signaling pathway and other signaling pathways [66, 67]. The KEGG results of this study showed that the signaling pathways interfered with by YZHG are mainly classical inflammatory pathways, including PI3K-Akt signaling pathway, Ras signaling pathway, MAPK signaling pathway, etc., suggesting that YZHG mainly treats ALD by regulating the inflammatory response. Inflammation and oxidative stress are key drivers of alcoholic liver injury [68]. Alcohol metabolism leads to oxidative stress and liver cell death. Liver oxidative stress promotes tissue damage, and then stimulates liver inflammation, forming a pathological cycle that promotes the progression of ALD [5]. Damaged liver cells release endogenous damage-associated molecular patterns, which in turn activate cellular pattern recognition receptors, leading to inflammation and production of pro-inflammatory cytokines (e.g., IL-1β, IL-2, etc.) [69]. In this study, the expression levels of pro-inflammatory cytokines (IL-1β, IL-2 and IL-6) and MDA in the liver of alcohol-fed model mice were increased, which was consistent with the results reported in ALD patients and animal experiments [70, 71]. However, YZHG can significantly reduce the expression levels of IL-1β, IL-2, IL-6 and MDA in liver, suggesting that YZHG plays a protective role by inhibiting liver inflammation and oxidative stress response.

## Conclusion

In conclusion, this study found that YZHG may be involved in the inflammatory response through multi-component, multi-target and multi-pathway to treat ALD. At the same time, animal experiments found that YZHG can reduce liver lipids, hepatic inflammatory response and oxidative stress response in ALD mice to exert a curative effect, which provides the experimental basis for clinical application.

## Supplementary Information

**Additional file 1: Table S1.** Basic information pertaining to the 82 active ingredients of YZHG. **Table S2.** GO enrichment analysis results. **Table S3.** KEGG enrichment analysis results.

## Data Availability

The relevant raw materials in this study can be directly contacted with the corresponding authors.

## Abbreviations

YZHG: Yinzhihuang granules
ALD: Alcoholic liver disease
UPLC-MS/MS: Ultra performance liquid chromatography tandem mass spectrometry
TCMSP: Traditional chinese medicine systems pharmacology database and analysis platform
OB: Oral bioavailability
DL: Drug-likeness
PPI: Protein–protein interaction
GO: Gene ontology
KEGG: Kyoto encyclopedia of genes and genomes
**C**: Control group
**M**: Model group
**P**: Positive group
**YL**: Yinzhihuang granules low dose group
**YM**: Yinzhihuang granules medium dose group
**YH**: Yinzhihuang granules high dose group
HE: Hematoxylin–eosin
TG: Triglycerides
ALT: Alanine aminotransferase
AST: Aspartate aminotransferase
SOD: Superoxide dismutase
MDA: Malondialdehyde
ELISA: Enzyme linked immunosorbent assay
IL-1β: Interleukin-1β
IL-2: Interleukin-2
IL-6: Interleukin-6
ESI: Electrospray ionization
BP: Biological process
MF: Molecular function
CC: Cellular components
SRC: Non-receptor tyrosine kinase
HSP90AA1: Heat shock protein 90 alpha family class A member 1
STAT3: Signal transducer and activator of transcription 3
EGFR: Epidermal growth factor receptor
AKT1: AKT serine/threonine kinase 1
