# Correction: Exploring the effect of Yinzhihuang granules on alcoholic liver disease based on pharmacodynamics, network pharmacology and molecular docking

**DOI:** 10.1186/s13020-023-00792-y

**Published:** 2023-07-10

**Authors:** Yingying Tan, Fanqin Zhang, Xiaotian Fan, Shan Lu, Yingying Liu, Zhishan Wu, Zhihong Huang, Chao Wu, Guoliang Cheng, Bing Li, Jiaqi Huang, Antony Stalin, Wei Zhou, Jiarui Wu

**Affiliations:** 1 Department of Clinical Chinese Pharmacy, School of Chinese Materia Medica, Beijing University of Chinese Medicine, Beijing, China; 2 State Key Laboratory of Generic Manufacture Technology of Chinese Traditional Medicine, Linyi, China; 3 Institute of Fundamental and Frontier Sciences, University of Electronic Science and Technology of China, Chengdu, China; 4 Department of Pharmacy, Friendship Hospital, Beijing, 100029 China

**Correction: Chinese Medicine (2023) 18:52** 10.1186/s13020-023-00759-z

Following publication of the original article [1], the authors reported an error in the Experimental animal model and drug interventions section.

The original content is as follows:

After 1 week of adaptive feeding, mice were randomly divided into 6 groups (n = 6), namely control group (C), model group (M), **3.5 mg/kg** of positive group (P), 3.69 g/kg of YZHG low dose group (YL), 7.38 g/kg of YZHG medium dose group (YM) and 14.76 g/kg of YZHG high dose group (YH). The doses of the positive drug (silibinin capsule) and YZHG were converted into corresponding doses based on the body surface area of humans and animals.

The revised content is as follows:

After 1 week of adaptive feeding, mice were randomly divided into 6 groups (n = 6), namely control group (C), model group (M), **43.05 mg/kg** of positive group (P), 3.69 g/kg of YZHG low dose group (YL), 7.38 g/kg of YZHG medium dose group (YM) and 14.76 g/kg of YZHG high dose group (YH). The doses of the positive drug (silibinin capsule) and YZHG were converted into corresponding doses based on the body surface area of humans and animals.

The original article [1] has been corrected.
