# Supplementary material for: Exploring the effect of Yinzhihuang granules on alcoholic liver disease based on pharmacodynamics, network pharmacology and molecular docking
Source: Chin Med. 2023 May 11;18:52. doi: 10.1186/s13020-023-00759-z (PMC10173499; doi:10.1186/s13020-023-00759-z)
Supplement: Supplementary file 1 — Additional file 1: Table S1. Basic information pertaining to the 82 active ingredients of YZHG. Table S2. GO enrichment analysis results. Table S3. KEGG enrichment analysis results. [file 13020_2023_759_MOESM1_ESM.docx]

| Table S1: Basic information pertaining to the 82 active ingredients of YZHG. | | | | |
| --- | --- | --- | --- | --- |
| **Ingredients** | **Mol ID** | **Code name** | **Herb** | **Source** |
| Isorhamnetin | MOL000354 | YC1 | Artemisiae scopariae herba | TCMSP |
| Areapillin | MOL004609 | YC2 | Artemisiae scopariae herba | TCMSP |
| Genkwanin | MOL005573 | YC3 | Artemisiae scopariae herba | TCMSP |
| Skrofulein | MOL007274 | YC4 | Artemisiae scopariae herba | TCMSP |
| Isoarcapillin | MOL008039 | YC5 | Artemisiae scopariae herba | TCMSP |
| Eupalitin | MOL008040 | YC6 | Artemisiae scopariae herba | TCMSP |
| Eupatolitin | MOL008041 | YC7 | Artemisiae scopariae herba | TCMSP |
| Capillarisin | MOL008043 | YC8 | Artemisiae scopariae herba | TCMSP |
| 4'-Methylcapillarisin | MOL008045 | YC9 | Artemisiae scopariae herba | TCMSP |
| Demethoxycapillarisin | MOL008046 | YC10 | Artemisiae scopariae herba | TCMSP |
| Artepillin A | MOL008047 | YC11 | Artemisiae scopariae herba | TCMSP |
| p-Hydroxyacetophenone | / | YC12 | Artemisiae scopariae herba | PubMed |
| Scoparone | / | YC13 | Artemisiae scopariae herba | PubMed |
| Ethyl oleate (NF) | MOL002883 | ZZ1 | Gardeniae fructus | TCMSP |
| Ammidin | MOL001941 | ZZ2 | Gardeniae fructus | TCMSP |
| Isoimperatorin | MOL001942 | ZZ3 | Gardeniae fructus | TCMSP |
| Crocetin | MOL001406 | ZZ4 | Gardeniae fructus | TCMSP |
| 3-Methylkempferol | MOL007245 | ZZ5 | Gardeniae fructus | TCMSP |
| Sudan III | MOL004561 | ZZ6 | Gardeniae fructus | TCMSP |
| (4aS,6aR,6aS,6bR,8aR,10R,12aR,14bS)-10-Hydroxy-2,2,6a,6b,9,9,12a-heptamethyl-1,3,4,5,6,6a,7,8,8a,10,11,12,13,14b-tetradecahydropicene-4a-carboxylic acid | MOL001663 | ZZ7 | Gardeniae fructus | TCMSP |
| GBGB | MOL009038 | ZZ8 | Gardeniae fructus | TCMSP |
| Shanzhiside | / | ZZ9 | Gardeniae fructus | PubMed |
| Geniposide | / | ZZ10 | Gardeniae fructus | PubMed |
| Geniposidic acid | / | ZZ11 | Gardeniae fructus | PubMed |
| Acacetin | MOL001689 | HQ1 | Scutellariae radix | TCMSP |
| Wogonin | MOL000173 | HQ2 | Scutellariae radix | TCMSP |
| (2R)-7-Hydroxy-5-methoxy-2-phenylchroman-4-one | MOL000228 | HQ3 | Scutellariae radix | TCMSP |
| Baicalein | MOL002714 | HQ4 | Scutellariae radix | TCMSP |
| 5,8,2'-Trihydroxy-7-methoxyflavone | MOL002908 | HQ5 | Scutellariae radix | TCMSP |
| 5,7,2,5-Tetrahydroxy-8,6-dimethoxyflavone | MOL002909 | HQ6 | Scutellariae radix | TCMSP |
| Carthamidin | MOL002910 | HQ7 | Scutellariae radix | TCMSP |
| Dihydrobaicalin_qt | MOL002913 | HQ8 | Scutellariae radix | TCMSP |
| Salvigenin | MOL002915 | HQ9 | Scutellariae radix | TCMSP |
| 5,2',6'-Trihydroxy-7,8-dimethoxyflavone | MOL002917 | HQ10 | Scutellariae radix | TCMSP |
| 5,7,2',6'-Tetrahydroxyflavone | MOL002925 | HQ11 | Scutellariae radix | TCMSP |
| Dihydrooroxylin A | MOL002926 | HQ12 | Scutellariae radix | TCMSP |
| Skullcapflavone II | MOL002927 | HQ13 | Scutellariae radix | TCMSP |
| Oroxylin A | MOL002928 | HQ14 | Scutellariae radix | TCMSP |
| Panicolin | MOL002932 | HQ15 | Scutellariae radix | TCMSP |
| 5,7,4'-Trihydroxy-8-methoxyflavone | MOL002933 | HQ16 | Scutellariae radix | TCMSP |
| DIHYDROOROXYLIN | MOL002937 | HQ17 | Scutellariae radix | TCMSP |
| Norwogonin | MOL000525 | HQ18 | Scutellariae radix | TCMSP |
| 5,2'-Dihydroxy-6,7,8-trimethoxyflavone | MOL000552 | HQ19 | Scutellariae radix | TCMSP |
| Coptisine | MOL001458 | HQ20 | Scutellariae radix | TCMSP |
| Bis[(2S)-2-ethylhexyl] benzene-1,2-dicarboxylate | MOL001490 | HQ21 | Scutellariae radix | TCMSP |
| Diop | MOL002879 | HQ22 | Scutellariae radix | TCMSP |
| Epiberberine | MOL002897 | HQ23 | Scutellariae radix | TCMSP |
| Moslosooflavone | MOL008206 | HQ24 | Scutellariae radix | TCMSP |
| 11,13-Eicosadienoic acid, methyl ester | MOL010415 | HQ25 | Scutellariae radix | TCMSP |
| 5,7,4'-Trihydroxy-6-methoxyflavanone | MOL012245 | HQ26 | Scutellariae radix | TCMSP |
| 5,7,4'-Trihydroxy-8-methoxyflavanone | MOL012246 | HQ27 | Scutellariae radix | TCMSP |
| Rivularin | MOL012266 | HQ28 | Scutellariae radix | TCMSP |
| Scutellarin | / | HQ29 | Scutellariae radix | PubMed |
| Baicalin | / | HQ30 | Scutellariae radix | PubMed |
| Wogonoside | / | HQ31 | Scutellariae radix | PubMed |
| Ethyl linolenate | MOL001495 | JYH1 | Lonicerae japonicae flos | TCMSP |
| Beta-carotene | MOL002773 | JYH2 | Lonicerae japonicae flos | TCMSP |
| ZINC03978781 | MOL003036 | JYH3 | Lonicerae japonicae flos | TCMSP |
| Chryseriol | MOL003044 | JYH4 | Lonicerae japonicae flos | TCMSP |
| 4,5'-Retro-.beta.,.beta.-Carotene-3,3'-dione, 4',5'-didehydro- | MOL003062 | JYH5 | Lonicerae japonicae flos | TCMSP |
| 7-epi-Vogeloside | MOL003101 | JYH6 | Lonicerae japonicae flos | TCMSP |
| Centauroside_qt | MOL003111 | JYH7 | Lonicerae japonicae flos | TCMSP |
| XYLOSTOSIDINE | MOL003124 | JYH8 | Lonicerae japonicae flos | TCMSP |
| Luteolin | MOL000006 | JYH9 | Lonicerae japonicae flos | TCMSP |
| Hyperoside | / | JYH10 | Lonicerae japonicae flos | PubMed |
| Caffeic acid | / | JYH11 | Lonicerae japonicae flos | PubMed |
| Luteoloside | / | JYH12 | Lonicerae japonicae flos | PubMed |
| Beta-sitosterol | MOL000358 | C1 | Artemisiae scopariae herba/Gardeniae fructus/Scutellariae radix/Lonicerae japonicae flos | TCMSP |
| Quercetin | MOL000098 | C2 | Artemisiae scopariae herba/Gardeniae fructus/Lonicerae japonicae flos | TCMSP |
| Mandenol | MOL001494 | C3 | Gardeniae fructus/Lonicerae japonicae flos | TCMSP |
| Kaempferol | MOL000422 | C4 | Gardeniae fructus/Lonicerae japonicae flos | TCMSP |
| 5-hydroxy-7-methoxy-2-(3,4,5-trimethoxyphenyl)chromone | MOL003095 | C5 | Gardeniae fructus/Lonicerae japonicae flos | TCMSP |
| Supraene | MOL001506 | C6 | Gardeniae fructus/Scutellariae radix | TCMSP |
| Stigmasterol | MOL000449 | C7 | Gardeniae fructus/Scutellariae radix/Lonicerae japonicae flos | TCMSP |
| Eriodyctiol (flavanone) | MOL002914 | C8 | Scutellariae radix/Lonicerae japonicae flos | TCMSP |
| Neochlorogenic acid（5-caffeoylquinic acid） | / | C9 | Artemisiae scopariae herba/Lonicerae japonicae flos | PubMed |
| Chlorogenic acid | / | C10 | Artemisiae scopariae herba/Lonicerae japonicae flos | PubMed |
| Cryptochlorogenic acid（4-Dicaffeoylquinic Acid） | / | C11 | Artemisiae scopariae herba/Lonicerae japonicae flos | PubMed |
| Isochlorogenic acid B | / | C12 | Artemisiae scopariae herba/Lonicerae japonicae flos | PubMed |
| Isochlorogenic acid A | / | C13 | Artemisiae scopariae herba/Lonicerae japonicae flos | PubMed |
| Isochlorogenic acid C | / | C14 | Artemisiae scopariae herba/Lonicerae japonicae flos | PubMed |
| 1,3-Dicaffeoylquinic acid | / | C15 | Artemisiae scopariae herba/Lonicerae japonicae flos | PubMed |

| Table S2. GO enrichment analysis results (P<0.05). | | | | |
| --- | --- | --- | --- | --- |
| ONTOLOGY | ID | Description | p-value | Count |
| BP | GO:0014065 | phosphatidylinositol 3-kinase signaling | 1.15E-28 | 21 |
| BP | GO:0018108 | peptidyl-tyrosine phosphorylation | 1.07E-27 | 26 |
| BP | GO:0018212 | peptidyl-tyrosine modification | 1.33E-27 | 26 |
| BP | GO:0048015 | phosphatidylinositol-mediated signaling | 9.70E-27 | 21 |
| BP | GO:0014066 | regulation of phosphatidylinositol 3-kinase signaling | 1.33E-26 | 19 |
| BP | GO:0048017 | inositol lipid-mediated signaling | 1.39E-26 | 21 |
| BP | GO:0014068 | positive regulation of phosphatidylinositol 3-kinase signaling | 8.18E-24 | 16 |
| BP | GO:0006979 | response to oxidative stress | 8.20E-24 | 25 |
| BP | GO:0062197 | cellular response to chemical stress | 1.54E-23 | 23 |
| BP | GO:0043491 | protein kinase B signaling | 4.87E-23 | 21 |
| BP | GO:2000379 | positive regulation of reactive oxygen species metabolic process | 1.26E-22 | 16 |
| BP | GO:0071902 | positive regulation of protein serine/threonine kinase activity | 1.61E-22 | 22 |
| BP | GO:0051896 | regulation of protein kinase B signaling | 2.30E-22 | 20 |
| BP | GO:0000302 | response to reactive oxygen species | 3.04E-21 | 19 |
| BP | GO:0032147 | activation of protein kinase activity | 4.34E-21 | 21 |
| BP | GO:2000377 | regulation of reactive oxygen species metabolic process | 4.36E-21 | 18 |
| BP | GO:0072593 | reactive oxygen species metabolic process | 4.88E-21 | 20 |
| BP | GO:0043405 | regulation of MAP kinase activity | 5.57E-21 | 21 |
| BP | GO:0070997 | neuron death | 1.09E-20 | 21 |
| BP | GO:0034599 | cellular response to oxidative stress | 1.67E-20 | 20 |
| BP | GO:0043406 | positive regulation of MAP kinase activity | 2.32E-20 | 19 |
| BP | GO:0051897 | positive regulation of protein kinase B signaling | 2.81E-20 | 17 |
| BP | GO:0048608 | reproductive structure development | 4.14E-20 | 22 |
| BP | GO:0061458 | reproductive system development | 4.81E-20 | 22 |
| BP | GO:0046777 | protein autophosphorylation | 1.31E-19 | 18 |
| BP | GO:0034614 | cellular response to reactive oxygen species | 5.18E-19 | 16 |
| BP | GO:0010001 | glial cell differentiation | 1.13E-18 | 17 |
| BP | GO:0070371 | ERK1 and ERK2 cascade | 1.15E-18 | 19 |
| BP | GO:0051222 | positive regulation of protein transport | 1.38E-18 | 21 |
| BP | GO:1904951 | positive regulation of establishment of protein localization | 2.86E-18 | 21 |
| BP | GO:0018105 | peptidyl-serine phosphorylation | 9.87E-18 | 18 |
| BP | GO:0070372 | regulation of ERK1 and ERK2 cascade | 1.05E-17 | 18 |
| BP | GO:1901214 | regulation of neuron death | 2.22E-17 | 18 |
| BP | GO:0070374 | positive regulation of ERK1 and ERK2 cascade | 2.81E-17 | 16 |
| BP | GO:0007568 | aging | 3.48E-17 | 18 |
| BP | GO:0018209 | peptidyl-serine modification | 3.68E-17 | 18 |
| BP | GO:0032496 | response to lipopolysaccharide | 5.67E-17 | 18 |
| BP | GO:0001819 | positive regulation of cytokine production | 7.63E-17 | 20 |
| BP | GO:0048708 | astrocyte differentiation | 7.76E-17 | 12 |
| BP | GO:0007596 | blood coagulation | 7.80E-17 | 18 |
| BP | GO:0007599 | hemostasis | 1.01E-16 | 18 |
| BP | GO:0050817 | coagulation | 1.07E-16 | 18 |
| BP | GO:0002237 | response to molecule of bacterial origin | 1.12E-16 | 18 |
| BP | GO:0042063 | gliogenesis | 1.42E-16 | 17 |
| BP | GO:0030168 | platelet activation | 1.81E-16 | 14 |
| BP | GO:0050727 | regulation of inflammatory response | 5.12E-16 | 18 |
| BP | GO:0052547 | regulation of peptidase activity | 8.25E-16 | 19 |
| BP | GO:0010506 | regulation of autophagy | 1.10E-15 | 17 |
| BP | GO:0022407 | regulation of cell-cell adhesion | 1.80E-15 | 18 |
| BP | GO:0030099 | myeloid cell differentiation | 3.27E-15 | 18 |
| BP | GO:0033002 | muscle cell proliferation | 3.93E-15 | 15 |
| BP | GO:0010038 | response to metal ion | 5.65E-15 | 17 |
| BP | GO:0051090 | regulation of DNA-binding transcription factor activity | 6.29E-15 | 18 |
| BP | GO:0050673 | epithelial cell proliferation | 6.81E-15 | 18 |
| BP | GO:0050730 | regulation of peptidyl-tyrosine phosphorylation | 1.09E-14 | 15 |
| BP | GO:0009314 | response to radiation | 1.18E-14 | 18 |
| BP | GO:1903409 | reactive oxygen species biosynthetic process | 1.28E-14 | 12 |
| BP | GO:0009612 | response to mechanical stimulus | 1.55E-14 | 14 |
| BP | GO:1903829 | positive regulation of cellular protein localization | 1.83E-14 | 16 |
| BP | GO:0046677 | response to antibiotic | 2.11E-14 | 16 |
| BP | GO:0042110 | T cell activation | 2.15E-14 | 18 |
| BP | GO:0071496 | cellular response to external stimulus | 3.69E-14 | 16 |
| BP | GO:2001233 | regulation of apoptotic signaling pathway | 3.70E-14 | 17 |
| BP | GO:0048661 | positive regulation of smooth muscle cell proliferation | 5.57E-14 | 11 |
| BP | GO:0006914 | autophagy | 6.75E-14 | 18 |
| BP | GO:0061919 | process utilizing autophagic mechanism | 6.75E-14 | 18 |
| BP | GO:0062013 | positive regulation of small molecule metabolic process | 7.44E-14 | 12 |
| BP | GO:0050900 | leukocyte migration | 7.49E-14 | 18 |
| BP | GO:0052548 | regulation of endopeptidase activity | 7.80E-14 | 17 |
| BP | GO:0071216 | cellular response to biotic stimulus | 7.81E-14 | 14 |
| BP | GO:0048511 | rhythmic process | 8.72E-14 | 15 |
| BP | GO:0009266 | response to temperature stimulus | 1.17E-13 | 14 |
| BP | GO:0043434 | response to peptide hormone | 1.18E-13 | 17 |
| BP | GO:0006809 | nitric oxide biosynthetic process | 1.36E-13 | 10 |
| BP | GO:0048872 | homeostasis of number of cells | 1.38E-13 | 14 |
| BP | GO:0046209 | nitric oxide metabolic process | 2.62E-13 | 10 |
| BP | GO:1901653 | cellular response to peptide | 2.62E-13 | 16 |
| BP | GO:2001057 | reactive nitrogen species metabolic process | 3.80E-13 | 10 |
| BP | GO:0071214 | cellular response to abiotic stimulus | 4.65E-13 | 15 |
| BP | GO:0104004 | cellular response to environmental stimulus | 4.65E-13 | 15 |
| BP | GO:0043281 | regulation of cysteine-type endopeptidase activity involved in apoptotic process | 5.20E-13 | 13 |
| BP | GO:0048660 | regulation of smooth muscle cell proliferation | 6.58E-13 | 12 |
| BP | GO:0019216 | regulation of lipid metabolic process | 6.87E-13 | 16 |
| BP | GO:0048659 | smooth muscle cell proliferation | 7.57E-13 | 12 |
| BP | GO:0001101 | response to acid chemical | 7.77E-13 | 15 |
| BP | GO:0097191 | extrinsic apoptotic signaling pathway | 8.79E-13 | 13 |
| BP | GO:2001234 | negative regulation of apoptotic signaling pathway | 1.23E-12 | 13 |
| BP | GO:0035690 | cellular response to drug | 1.57E-12 | 16 |
| BP | GO:0050804 | modulation of chemical synaptic transmission | 1.75E-12 | 16 |
| BP | GO:0099177 | regulation of trans-synaptic signaling | 1.81E-12 | 16 |
| BP | GO:2000116 | regulation of cysteine-type endopeptidase activity | 2.01E-12 | 13 |
| BP | GO:0045429 | positive regulation of nitric oxide biosynthetic process | 2.07E-12 | 8 |
| BP | GO:1903426 | regulation of reactive oxygen species biosynthetic process | 2.23E-12 | 10 |
| BP | GO:0032386 | regulation of intracellular transport | 2.31E-12 | 15 |
| BP | GO:0038127 | ERBB signaling pathway | 2.50E-12 | 11 |
| BP | GO:1904407 | positive regulation of nitric oxide metabolic process | 2.52E-12 | 8 |
| BP | GO:0010517 | regulation of phospholipase activity | 2.68E-12 | 9 |
| BP | GO:0050731 | positive regulation of peptidyl-tyrosine phosphorylation | 3.00E-12 | 12 |
| BP | GO:1900274 | regulation of phospholipase C activity | 3.06E-12 | 8 |
| BP | GO:0033138 | positive regulation of peptidyl-serine phosphorylation | 3.32E-12 | 10 |
| BP | GO:0045834 | positive regulation of lipid metabolic process | 3.40E-12 | 11 |
| BP | GO:0034612 | response to tumor necrosis factor | 3.51E-12 | 14 |
| BP | GO:0042136 | neurotransmitter biosynthetic process | 3.65E-12 | 10 |
| BP | GO:0031349 | positive regulation of defense response | 3.93E-12 | 15 |
| BP | GO:0010952 | positive regulation of peptidase activity | 4.07E-12 | 12 |
| BP | GO:0150076 | neuroinflammatory response | 5.11E-12 | 9 |
| BP | GO:0000187 | activation of MAPK activity | 5.29E-12 | 11 |
| BP | GO:0002573 | myeloid leukocyte differentiation | 6.15E-12 | 12 |
| BP | GO:0071222 | cellular response to lipopolysaccharide | 6.51E-12 | 12 |
| BP | GO:0071219 | cellular response to molecule of bacterial origin | 9.66E-12 | 12 |
| BP | GO:0010631 | epithelial cell migration | 1.77E-11 | 14 |
| BP | GO:0062012 | regulation of small molecule metabolic process | 1.90E-11 | 15 |
| BP | GO:0016241 | regulation of macroautophagy | 1.91E-11 | 11 |
| BP | GO:0051403 | stress-activated MAPK cascade | 1.93E-11 | 13 |
| BP | GO:0090132 | epithelium migration | 1.98E-11 | 14 |
| BP | GO:0007162 | negative regulation of cell adhesion | 2.20E-11 | 13 |
| BP | GO:0097193 | intrinsic apoptotic signaling pathway | 2.20E-11 | 13 |
| BP | GO:0045862 | positive regulation of proteolysis | 2.22E-11 | 14 |
| BP | GO:0048732 | gland development | 2.23E-11 | 15 |
| BP | GO:1903428 | positive regulation of reactive oxygen species biosynthetic process | 2.27E-11 | 8 |
| BP | GO:0032388 | positive regulation of intracellular transport | 2.39E-11 | 12 |
| BP | GO:0090130 | tissue migration | 2.48E-11 | 14 |
| BP | GO:0009408 | response to heat | 2.62E-11 | 11 |
| BP | GO:0090316 | positive regulation of intracellular protein transport | 2.62E-11 | 11 |
| BP | GO:1903034 | regulation of response to wounding | 3.14E-11 | 11 |
| BP | GO:0006909 | phagocytosis | 3.32E-11 | 14 |
| BP | GO:0051402 | neuron apoptotic process | 3.93E-11 | 12 |
| BP | GO:1903037 | regulation of leukocyte cell-cell adhesion | 4.13E-11 | 13 |
| BP | GO:0038093 | Fc receptor signaling pathway | 4.33E-11 | 12 |
| BP | GO:0042593 | glucose homeostasis | 4.33E-11 | 12 |
| BP | GO:0033500 | carbohydrate homeostasis | 4.54E-11 | 12 |
| BP | GO:0042113 | B cell activation | 5.27E-11 | 13 |
| BP | GO:0002433 | immune response-regulating cell surface receptor signaling pathway involved in phagocytosis | 5.58E-11 | 10 |
| BP | GO:0033135 | regulation of peptidyl-serine phosphorylation | 5.58E-11 | 10 |
| BP | GO:0038096 | Fc-gamma receptor signaling pathway involved in phagocytosis | 5.58E-11 | 10 |
| BP | GO:0060759 | regulation of response to cytokine stimulus | 5.98E-11 | 11 |
| BP | GO:0060191 | regulation of lipase activity | 6.01E-11 | 9 |
| BP | GO:0031098 | stress-activated protein kinase signaling cascade | 6.43E-11 | 13 |
| BP | GO:0007409 | axonogenesis | 6.45E-11 | 15 |
| BP | GO:0033157 | regulation of intracellular protein transport | 6.63E-11 | 12 |
| BP | GO:0038094 | Fc-gamma receptor signaling pathway | 6.90E-11 | 10 |
| BP | GO:0002429 | immune response-activating cell surface receptor signaling pathway | 7.49E-11 | 15 |
| BP | GO:0002757 | immune response-activating signal transduction | 7.49E-11 | 15 |
| BP | GO:0045428 | regulation of nitric oxide biosynthetic process | 7.69E-11 | 8 |
| BP | GO:0070482 | response to oxygen levels | 7.90E-11 | 14 |
| BP | GO:1903706 | regulation of hemopoiesis | 7.94E-11 | 15 |
| BP | GO:0071375 | cellular response to peptide hormone stimulus | 8.13E-11 | 13 |
| BP | GO:0002431 | Fc receptor mediated stimulatory signaling pathway | 8.50E-11 | 10 |
| BP | GO:0061041 | regulation of wound healing | 1.04E-10 | 10 |
| BP | GO:0045785 | positive regulation of cell adhesion | 1.06E-10 | 14 |
| BP | GO:0051091 | positive regulation of DNA-binding transcription factor activity | 1.09E-10 | 12 |
| BP | GO:0001890 | placenta development | 1.36E-10 | 10 |
| BP | GO:0042133 | neurotransmitter metabolic process | 1.45E-10 | 10 |
| BP | GO:0010863 | positive regulation of phospholipase C activity | 1.45E-10 | 7 |
| BP | GO:0007159 | leukocyte cell-cell adhesion | 1.48E-10 | 13 |
| BP | GO:0031667 | response to nutrient levels | 1.58E-10 | 15 |
| BP | GO:1901215 | negative regulation of neuron death | 1.58E-10 | 11 |
| BP | GO:0007548 | sex differentiation | 1.61E-10 | 12 |
| BP | GO:0032868 | response to insulin | 1.76E-10 | 12 |
| BP | GO:0043254 | regulation of protein-containing complex assembly | 2.42E-10 | 14 |
| BP | GO:0008406 | gonad development | 2.49E-10 | 11 |
| BP | GO:0043393 | regulation of protein binding | 2.49E-10 | 11 |
| BP | GO:0007569 | cell aging | 2.77E-10 | 9 |
| BP | GO:0060324 | face development | 3.27E-10 | 7 |
| BP | GO:0045137 | development of primary sexual characteristics | 3.33E-10 | 11 |
| BP | GO:0030193 | regulation of blood coagulation | 3.37E-10 | 8 |
| BP | GO:0071260 | cellular response to mechanical stimulus | 3.37E-10 | 8 |
| BP | GO:0031331 | positive regulation of cellular catabolic process | 3.58E-10 | 13 |
| BP | GO:1900046 | regulation of hemostasis | 3.73E-10 | 8 |
| BP | GO:0071356 | cellular response to tumor necrosis factor | 3.81E-10 | 12 |
| BP | GO:0010634 | positive regulation of epithelial cell migration | 4.32E-10 | 10 |
| BP | GO:0050890 | cognition | 4.63E-10 | 12 |
| BP | GO:0051098 | regulation of binding | 5.16E-10 | 13 |
| BP | GO:0050818 | regulation of coagulation | 5.55E-10 | 8 |
| BP | GO:0001959 | regulation of cytokine-mediated signaling pathway | 6.06E-10 | 10 |
| BP | GO:0050678 | regulation of epithelial cell proliferation | 6.07E-10 | 13 |
| BP | GO:0060326 | cell chemotaxis | 6.28E-10 | 12 |
| BP | GO:0001667 | ameboidal-type cell migration | 6.34E-10 | 14 |
| BP | GO:0032872 | regulation of stress-activated MAPK cascade | 6.36E-10 | 11 |
| BP | GO:0051346 | negative regulation of hydrolase activity | 6.52E-10 | 14 |
| BP | GO:0030258 | lipid modification | 6.65E-10 | 11 |
| BP | GO:0070302 | regulation of stress-activated protein kinase signaling cascade | 6.95E-10 | 11 |
| BP | GO:0048545 | response to steroid hormone | 7.12E-10 | 13 |
| BP | GO:0009895 | negative regulation of catabolic process | 7.29E-10 | 12 |
| BP | GO:0042326 | negative regulation of phosphorylation | 7.50E-10 | 14 |
| BP | GO:0009416 | response to light stimulus | 9.08E-10 | 12 |
| BP | GO:1903708 | positive regulation of hemopoiesis | 9.33E-10 | 10 |
| BP | GO:0051235 | maintenance of location | 9.42E-10 | 12 |
| BP | GO:0010543 | regulation of platelet activation | 1.06E-09 | 6 |
| BP | GO:0071248 | cellular response to metal ion | 1.09E-09 | 10 |
| BP | GO:0034605 | cellular response to heat | 1.23E-09 | 9 |
| BP | GO:0001935 | endothelial cell proliferation | 1.27E-09 | 10 |
| BP | GO:0051054 | positive regulation of DNA metabolic process | 1.27E-09 | 10 |
| BP | GO:0031663 | lipopolysaccharide-mediated signaling pathway | 1.30E-09 | 7 |
| BP | GO:0061900 | glial cell activation | 1.30E-09 | 7 |
| BP | GO:1903532 | positive regulation of secretion by cell | 1.32E-09 | 13 |
| BP | GO:1901216 | positive regulation of neuron death | 1.38E-09 | 8 |
| BP | GO:0007611 | learning or memory | 1.43E-09 | 11 |
| BP | GO:0010518 | positive regulation of phospholipase activity | 1.47E-09 | 7 |
| BP | GO:0042116 | macrophage activation | 1.50E-09 | 8 |
| BP | GO:0008585 | female gonad development | 1.63E-09 | 8 |
| BP | GO:0034341 | response to interferon-gamma | 1.90E-09 | 10 |
| BP | GO:0046824 | positive regulation of nucleocytoplasmic transport | 2.10E-09 | 7 |
| BP | GO:0002262 | myeloid cell homeostasis | 2.30E-09 | 9 |
| BP | GO:1901342 | regulation of vasculature development | 2.31E-09 | 13 |
| BP | GO:0031668 | cellular response to extracellular stimulus | 2.32E-09 | 11 |
| BP | GO:0050714 | positive regulation of protein secretion | 2.32E-09 | 11 |
| BP | GO:0046686 | response to cadmium ion | 2.35E-09 | 7 |
| BP | GO:0046545 | development of primary female sexual characteristics | 2.45E-09 | 8 |
| BP | GO:0009896 | positive regulation of catabolic process | 2.51E-09 | 13 |
| BP | GO:0060135 | maternal process involved in female pregnancy | 2.64E-09 | 7 |
| BP | GO:0050679 | positive regulation of epithelial cell proliferation | 2.65E-09 | 10 |
| BP | GO:1902105 | regulation of leukocyte differentiation | 2.71E-09 | 11 |
| BP | GO:0051047 | positive regulation of secretion | 2.73E-09 | 13 |
| BP | GO:0007623 | circadian rhythm | 2.91E-09 | 10 |
| BP | GO:0032768 | regulation of monooxygenase activity | 2.95E-09 | 7 |
| BP | GO:0071229 | cellular response to acid chemical | 3.05E-09 | 10 |
| BP | GO:0046822 | regulation of nucleocytoplasmic transport | 3.10E-09 | 8 |
| BP | GO:2001237 | negative regulation of extrinsic apoptotic signaling pathway | 3.10E-09 | 8 |
| BP | GO:0051052 | regulation of DNA metabolic process | 3.20E-09 | 12 |
| BP | GO:0050729 | positive regulation of inflammatory response | 3.27E-09 | 9 |
| BP | GO:0038083 | peptidyl-tyrosine autophosphorylation | 3.30E-09 | 6 |
| BP | GO:0071276 | cellular response to cadmium ion | 3.30E-09 | 6 |
| BP | GO:0030098 | lymphocyte differentiation | 3.41E-09 | 12 |
| BP | GO:0042108 | positive regulation of cytokine biosynthetic process | 3.66E-09 | 7 |
| BP | GO:2001236 | regulation of extrinsic apoptotic signaling pathway | 3.67E-09 | 9 |
| BP | GO:1905952 | regulation of lipid localization | 3.88E-09 | 9 |
| BP | GO:0051341 | regulation of oxidoreductase activity | 3.89E-09 | 8 |
| BP | GO:0071241 | cellular response to inorganic substance | 4.00E-09 | 10 |
| BP | GO:0043467 | regulation of generation of precursor metabolites and energy | 4.11E-09 | 9 |
| BP | GO:0001666 | response to hypoxia | 4.12E-09 | 12 |
| BP | GO:0042698 | ovulation cycle | 4.52E-09 | 7 |
| BP | GO:1903039 | positive regulation of leukocyte cell-cell adhesion | 4.57E-09 | 10 |
| BP | GO:0002793 | positive regulation of peptide secretion | 4.92E-09 | 11 |
| BP | GO:1904019 | epithelial cell apoptotic process | 5.22E-09 | 8 |
| BP | GO:0010632 | regulation of epithelial cell migration | 5.48E-09 | 11 |
| BP | GO:0036293 | response to decreased oxygen levels | 5.78E-09 | 12 |
| BP | GO:0043200 | response to amino acid | 6.01E-09 | 8 |
| BP | GO:0060193 | positive regulation of lipase activity | 6.11E-09 | 7 |
| BP | GO:0016236 | macroautophagy | 6.32E-09 | 11 |
| BP | GO:0006801 | superoxide metabolic process | 6.74E-09 | 7 |
| BP | GO:0046660 | female sex differentiation | 6.91E-09 | 8 |
| BP | GO:0070661 | leukocyte proliferation | 7.02E-09 | 11 |
| BP | GO:1900182 | positive regulation of protein localization to nucleus | 7.43E-09 | 7 |
| BP | GO:0050806 | positive regulation of synaptic transmission | 7.44E-09 | 9 |
| BP | GO:1904018 | positive regulation of vasculature development | 7.64E-09 | 10 |
| BP | GO:0045765 | regulation of angiogenesis | 8.49E-09 | 12 |
| BP | GO:0050708 | regulation of protein secretion | 8.82E-09 | 13 |
| BP | GO:0007173 | epidermal growth factor receptor signaling pathway | 9.06E-09 | 8 |
| BP | GO:0031669 | cellular response to nutrient levels | 1.02E-08 | 10 |
| BP | GO:1905477 | positive regulation of protein localization to membrane | 1.10E-08 | 8 |
| BP | GO:0001936 | regulation of endothelial cell proliferation | 1.12E-08 | 9 |
| BP | GO:0030217 | T cell differentiation | 1.15E-08 | 10 |
| BP | GO:0043536 | positive regulation of blood vessel endothelial cell migration | 1.18E-08 | 7 |
| BP | GO:0016049 | cell growth | 1.19E-08 | 13 |
| BP | GO:0031281 | positive regulation of cyclase activity | 1.19E-08 | 5 |
| BP | GO:0050863 | regulation of T cell activation | 1.21E-08 | 11 |
| BP | GO:0010950 | positive regulation of endopeptidase activity | 1.23E-08 | 9 |
| BP | GO:0048871 | multicellular organismal homeostasis | 1.25E-08 | 13 |
| BP | GO:0022602 | ovulation cycle process | 1.30E-08 | 6 |
| BP | GO:0071354 | cellular response to interleukin-6 | 1.30E-08 | 6 |
| BP | GO:0022408 | negative regulation of cell-cell adhesion | 1.36E-08 | 9 |
| BP | GO:0071346 | cellular response to interferon-gamma | 1.36E-08 | 9 |
| BP | GO:0018107 | peptidyl-threonine phosphorylation | 1.42E-08 | 8 |
| BP | GO:0070849 | response to epidermal growth factor | 1.48E-08 | 6 |
| BP | GO:0097237 | cellular response to toxic substance | 1.51E-08 | 10 |
| BP | GO:0010595 | positive regulation of endothelial cell migration | 1.61E-08 | 8 |
| BP | GO:0001774 | microglial cell activation | 1.69E-08 | 6 |
| BP | GO:0002269 | leukocyte activation involved in inflammatory response | 1.69E-08 | 6 |
| BP | GO:0002791 | regulation of peptide secretion | 1.75E-08 | 13 |
| BP | GO:0048167 | regulation of synaptic plasticity | 1.89E-08 | 9 |
| BP | GO:1905475 | regulation of protein localization to membrane | 1.89E-08 | 9 |
| BP | GO:1990090 | cellular response to nerve growth factor stimulus | 1.92E-08 | 6 |
| BP | GO:0022409 | positive regulation of cell-cell adhesion | 2.04E-08 | 10 |
| BP | GO:0043280 | positive regulation of cysteine-type endopeptidase activity involved in apoptotic process | 2.05E-08 | 8 |
| BP | GO:0001776 | leukocyte homeostasis | 2.15E-08 | 7 |
| BP | GO:0008625 | extrinsic apoptotic signaling pathway via death domain receptors | 2.15E-08 | 7 |
| BP | GO:0032760 | positive regulation of tumor necrosis factor production | 2.15E-08 | 7 |
| BP | GO:0070741 | response to interleukin-6 | 2.18E-08 | 6 |
| BP | GO:0018210 | peptidyl-threonine modification | 2.31E-08 | 8 |
| BP | GO:0032355 | response to estradiol | 2.31E-08 | 8 |
| BP | GO:0007565 | female pregnancy | 2.38E-08 | 9 |
| BP | GO:0017038 | protein import | 2.38E-08 | 9 |
| BP | GO:0050999 | regulation of nitric-oxide synthase activity | 2.46E-08 | 6 |
| BP | GO:1903557 | positive regulation of tumor necrosis factor superfamily cytokine production | 2.52E-08 | 7 |
| BP | GO:1904035 | regulation of epithelial cell apoptotic process | 2.52E-08 | 7 |
| BP | GO:0034504 | protein localization to nucleus | 2.64E-08 | 10 |
| BP | GO:1990089 | response to nerve growth factor | 2.77E-08 | 6 |
| BP | GO:0032800 | receptor biosynthetic process | 2.94E-08 | 5 |
| BP | GO:0006913 | nucleocytoplasmic transport | 2.99E-08 | 11 |
| BP | GO:0000186 | activation of MAPKK activity | 3.12E-08 | 6 |
| BP | GO:0030195 | negative regulation of blood coagulation | 3.12E-08 | 6 |
| BP | GO:0051169 | nuclear transport | 3.27E-08 | 11 |
| BP | GO:0048638 | regulation of developmental growth | 3.37E-08 | 11 |
| BP | GO:0009411 | response to UV | 3.44E-08 | 8 |
| BP | GO:0050994 | regulation of lipid catabolic process | 3.50E-08 | 6 |
| BP | GO:1900047 | negative regulation of hemostasis | 3.50E-08 | 6 |
| BP | GO:1904645 | response to amyloid-beta | 3.50E-08 | 6 |
| BP | GO:0050852 | T cell receptor signaling pathway | 3.69E-08 | 9 |
| BP | GO:0036473 | cell death in response to oxidative stress | 3.71E-08 | 7 |
| BP | GO:1901655 | cellular response to ketone | 3.71E-08 | 7 |
| BP | GO:0046651 | lymphocyte proliferation | 3.76E-08 | 10 |
| BP | GO:0006606 | protein import into nucleus | 3.84E-08 | 8 |
| BP | GO:0035265 | organ growth | 4.02E-08 | 9 |
| BP | GO:0045766 | positive regulation of angiogenesis | 4.02E-08 | 9 |
| BP | GO:0032943 | mononuclear cell proliferation | 4.03E-08 | 10 |
| BP | GO:0043542 | endothelial cell migration | 4.03E-08 | 10 |
| BP | GO:0001505 | regulation of neurotransmitter levels | 4.25E-08 | 11 |
| BP | GO:0007411 | axon guidance | 4.31E-08 | 10 |
| BP | GO:0097485 | neuron projection guidance | 4.46E-08 | 10 |
| BP | GO:0042542 | response to hydrogen peroxide | 4.52E-08 | 8 |
| BP | GO:0070106 | interleukin-27-mediated signaling pathway | 4.61E-08 | 4 |
| BP | GO:0070757 | interleukin-35-mediated signaling pathway | 4.61E-08 | 4 |
| BP | GO:0030522 | intracellular receptor signaling pathway | 4.61E-08 | 10 |
| BP | GO:0019217 | regulation of fatty acid metabolic process | 4.63E-08 | 7 |
| BP | GO:0048010 | vascular endothelial growth factor receptor signaling pathway | 4.63E-08 | 7 |
| BP | GO:0010212 | response to ionizing radiation | 4.76E-08 | 8 |
| BP | GO:0048008 | platelet-derived growth factor receptor signaling pathway | 4.87E-08 | 6 |
| BP | GO:0050819 | negative regulation of coagulation | 4.87E-08 | 6 |
| BP | GO:0043523 | regulation of neuron apoptotic process | 5.16E-08 | 9 |
| BP | GO:0070102 | interleukin-6-mediated signaling pathway | 5.26E-08 | 5 |
| BP | GO:2001056 | positive regulation of cysteine-type endopeptidase activity | 5.29E-08 | 8 |
| BP | GO:0007254 | JNK cascade | 6.07E-08 | 9 |
| BP | GO:0006874 | cellular calcium ion homeostasis | 6.10E-08 | 12 |
| BP | GO:0001678 | cellular glucose homeostasis | 6.18E-08 | 8 |
| BP | GO:0034250 | positive regulation of cellular amide metabolic process | 6.18E-08 | 8 |
| BP | GO:0032869 | cellular response to insulin stimulus | 6.57E-08 | 9 |
| BP | GO:0019722 | calcium-mediated signaling | 7.11E-08 | 9 |
| BP | GO:0090303 | positive regulation of wound healing | 7.38E-08 | 6 |
| BP | GO:0032091 | negative regulation of protein binding | 7.55E-08 | 7 |
| BP | GO:0043535 | regulation of blood vessel endothelial cell migration | 7.56E-08 | 8 |
| BP | GO:0071887 | leukocyte apoptotic process | 8.07E-08 | 7 |
| BP | GO:0055074 | calcium ion homeostasis | 8.27E-08 | 12 |
| BP | GO:0044706 | multi-multicellular organism process | 8.31E-08 | 9 |
| BP | GO:1903522 | regulation of blood circulation | 8.57E-08 | 10 |
| BP | GO:0007259 | receptor signaling pathway via JAK-STAT | 8.76E-08 | 8 |
| BP | GO:0038128 | ERBB2 signaling pathway | 8.85E-08 | 5 |
| BP | GO:0006631 | fatty acid metabolic process | 9.17E-08 | 11 |
| BP | GO:0034248 | regulation of cellular amide metabolic process | 9.28E-08 | 12 |
| BP | GO:0048011 | neurotrophin TRK receptor signaling pathway | 1.04E-07 | 5 |
| BP | GO:0002526 | acute inflammatory response | 1.05E-07 | 7 |
| BP | GO:0051170 | import into nucleus | 1.06E-07 | 8 |
| BP | GO:0010594 | regulation of endothelial cell migration | 1.08E-07 | 9 |
| BP | GO:0045088 | regulation of innate immune response | 1.10E-07 | 10 |
| BP | GO:0009743 | response to carbohydrate | 1.12E-07 | 9 |
| BP | GO:0051249 | regulation of lymphocyte activation | 1.14E-07 | 12 |
| BP | GO:0050867 | positive regulation of cell activation | 1.22E-07 | 11 |
| BP | GO:0043279 | response to alkaloid | 1.35E-07 | 7 |
| BP | GO:0072503 | cellular divalent inorganic cation homeostasis | 1.36E-07 | 12 |
| BP | GO:0036092 | phosphatidylinositol-3-phosphate biosynthetic process | 1.39E-07 | 4 |
| BP | GO:0051100 | negative regulation of binding | 1.40E-07 | 8 |
| BP | GO:0097696 | receptor signaling pathway via STAT | 1.40E-07 | 8 |
| BP | GO:0002831 | regulation of response to biotic stimulus | 1.42E-07 | 11 |
| BP | GO:0071230 | cellular response to amino acid stimulus | 1.43E-07 | 6 |
| BP | GO:0030218 | erythrocyte differentiation | 1.52E-07 | 7 |
| BP | GO:0042035 | regulation of cytokine biosynthetic process | 1.52E-07 | 7 |
| BP | GO:0050851 | antigen receptor-mediated signaling pathway | 1.53E-07 | 10 |
| BP | GO:0030324 | lung development | 1.61E-07 | 8 |
| BP | GO:1900542 | regulation of purine nucleotide metabolic process | 1.61E-07 | 7 |
| BP | GO:0090322 | regulation of superoxide metabolic process | 1.64E-07 | 5 |
| BP | GO:0007204 | positive regulation of cytosolic calcium ion concentration | 1.67E-07 | 10 |
| BP | GO:0021782 | glial cell development | 1.71E-07 | 7 |
| BP | GO:1900180 | regulation of protein localization to nucleus | 1.71E-07 | 7 |
| BP | GO:0006140 | regulation of nucleotide metabolic process | 1.82E-07 | 7 |
| BP | GO:0031334 | positive regulation of protein-containing complex assembly | 1.86E-07 | 9 |
| BP | GO:0045410 | positive regulation of interleukin-6 biosynthetic process | 1.89E-07 | 4 |
| BP | GO:0051770 | positive regulation of nitric-oxide synthase biosynthetic process | 1.89E-07 | 4 |
| BP | GO:0042307 | positive regulation of protein import into nucleus | 1.89E-07 | 5 |
| BP | GO:0030323 | respiratory tube development | 1.92E-07 | 8 |
| BP | GO:0002532 | production of molecular mediator involved in inflammatory response | 2.01E-07 | 6 |
| BP | GO:0061180 | mammary gland epithelium development | 2.01E-07 | 6 |
| BP | GO:0001558 | regulation of cell growth | 2.10E-07 | 11 |
| BP | GO:0071383 | cellular response to steroid hormone stimulus | 2.13E-07 | 9 |
| BP | GO:0048771 | tissue remodeling | 2.18E-07 | 8 |
| BP | GO:0043534 | blood vessel endothelial cell migration | 2.28E-07 | 8 |
| BP | GO:0010951 | negative regulation of endopeptidase activity | 2.28E-07 | 9 |
| BP | GO:0045637 | regulation of myeloid cell differentiation | 2.36E-07 | 9 |
| BP | GO:1903036 | positive regulation of response to wounding | 2.37E-07 | 6 |
| BP | GO:0034101 | erythrocyte homeostasis | 2.42E-07 | 7 |
| BP | GO:0038179 | neurotrophin signaling pathway | 2.48E-07 | 5 |
| BP | GO:1902042 | negative regulation of extrinsic apoptotic signaling pathway via death domain receptors | 2.48E-07 | 5 |
| BP | GO:1904591 | positive regulation of protein import | 2.48E-07 | 5 |
| BP | GO:0090335 | regulation of brown fat cell differentiation | 2.51E-07 | 4 |
| BP | GO:0042089 | cytokine biosynthetic process | 2.56E-07 | 7 |
| BP | GO:0051924 | regulation of calcium ion transport | 2.61E-07 | 9 |
| BP | GO:0042098 | T cell proliferation | 2.69E-07 | 8 |
| BP | GO:0042107 | cytokine metabolic process | 2.71E-07 | 7 |
| BP | GO:0061045 | negative regulation of wound healing | 2.79E-07 | 6 |
| BP | GO:0048568 | embryonic organ development | 2.79E-07 | 11 |
| BP | GO:0046328 | regulation of JNK cascade | 2.81E-07 | 8 |
| BP | GO:0001933 | negative regulation of protein phosphorylation | 2.86E-07 | 11 |
| BP | GO:0048863 | stem cell differentiation | 2.88E-07 | 9 |
| BP | GO:0090398 | cellular senescence | 3.26E-07 | 6 |
| BP | GO:0010466 | negative regulation of peptidase activity | 3.39E-07 | 9 |
| BP | GO:0045913 | positive regulation of carbohydrate metabolic process | 3.51E-07 | 6 |
| BP | GO:1900034 | regulation of cellular response to heat | 3.51E-07 | 6 |
| BP | GO:0019932 | second-messenger-mediated signaling | 3.60E-07 | 11 |
| BP | GO:1901888 | regulation of cell junction assembly | 3.73E-07 | 8 |
| BP | GO:0008631 | intrinsic apoptotic signaling pathway in response to oxidative stress | 4.11E-07 | 5 |
| BP | GO:0031279 | regulation of cyclase activity | 4.11E-07 | 5 |
| BP | GO:0045598 | regulation of fat cell differentiation | 4.14E-07 | 7 |
| BP | GO:0045927 | positive regulation of growth | 4.36E-07 | 9 |
| BP | GO:0032637 | interleukin-8 production | 4.39E-07 | 6 |
| BP | GO:0014002 | astrocyte development | 4.62E-07 | 5 |
| BP | GO:0035307 | positive regulation of protein dephosphorylation | 4.62E-07 | 5 |
| BP | GO:0051480 | regulation of cytosolic calcium ion concentration | 4.70E-07 | 10 |
| BP | GO:0060541 | respiratory system development | 4.71E-07 | 8 |
| BP | GO:0045861 | negative regulation of proteolysis | 4.82E-07 | 10 |
| BP | GO:0006109 | regulation of carbohydrate metabolic process | 4.90E-07 | 8 |
| BP | GO:0050866 | negative regulation of cell activation | 4.90E-07 | 8 |
| BP | GO:0010507 | negative regulation of autophagy | 5.07E-07 | 6 |
| BP | GO:0043154 | negative regulation of cysteine-type endopeptidase activity involved in apoptotic process | 5.07E-07 | 6 |
| BP | GO:0048538 | thymus development | 5.18E-07 | 5 |
| BP | GO:0010544 | negative regulation of platelet activation | 5.30E-07 | 4 |
| BP | GO:0032930 | positive regulation of superoxide anion generation | 5.30E-07 | 4 |
| BP | GO:0046425 | regulation of receptor signaling pathway via JAK-STAT | 5.33E-07 | 7 |
| BP | GO:1904705 | regulation of vascular smooth muscle cell proliferation | 5.44E-07 | 6 |
| BP | GO:1990874 | vascular smooth muscle cell proliferation | 5.44E-07 | 6 |
| BP | GO:0009746 | response to hexose | 5.48E-07 | 8 |
| BP | GO:0050870 | positive regulation of T cell activation | 5.48E-07 | 8 |
| BP | GO:0008584 | male gonad development | 5.60E-07 | 7 |
| BP | GO:0046631 | alpha-beta T cell activation | 5.60E-07 | 7 |
| BP | GO:0010883 | regulation of lipid storage | 5.80E-07 | 5 |
| BP | GO:0045981 | positive regulation of nucleotide metabolic process | 5.80E-07 | 5 |
| BP | GO:1900544 | positive regulation of purine nucleotide metabolic process | 5.80E-07 | 5 |
| BP | GO:1904036 | negative regulation of epithelial cell apoptotic process | 5.80E-07 | 5 |
| BP | GO:0006919 | activation of cysteine-type endopeptidase activity involved in apoptotic process | 5.83E-07 | 6 |
| BP | GO:0046546 | development of primary male sexual characteristics | 5.88E-07 | 7 |
| BP | GO:0050715 | positive regulation of cytokine secretion | 5.88E-07 | 7 |
| BP | GO:0006006 | glucose metabolic process | 5.91E-07 | 8 |
| BP | GO:0002697 | regulation of immune effector process | 5.96E-07 | 11 |
| BP | GO:0030198 | extracellular matrix organization | 6.20E-07 | 10 |
| BP | GO:0001892 | embryonic placenta development | 6.24E-07 | 6 |
| BP | GO:0043062 | extracellular structure organization | 6.35E-07 | 10 |
| BP | GO:0051258 | protein polymerization | 6.47E-07 | 9 |
| BP | GO:0045058 | T cell selection | 6.47E-07 | 5 |
| BP | GO:0061614 | pri-miRNA transcription by RNA polymerase II | 6.47E-07 | 5 |
| BP | GO:0008286 | insulin receptor signaling pathway | 6.48E-07 | 7 |
| BP | GO:0034284 | response to monosaccharide | 6.60E-07 | 8 |
| BP | GO:0042535 | positive regulation of tumor necrosis factor biosynthetic process | 6.61E-07 | 4 |
| BP | GO:0051767 | nitric-oxide synthase biosynthetic process | 6.61E-07 | 4 |
| BP | GO:0051769 | regulation of nitric-oxide synthase biosynthetic process | 6.61E-07 | 4 |
| BP | GO:0031647 | regulation of protein stability | 6.66E-07 | 9 |
| BP | GO:1900407 | regulation of cellular response to oxidative stress | 6.68E-07 | 6 |
| BP | GO:0051348 | negative regulation of transferase activity | 6.86E-07 | 9 |
| BP | GO:0001701 | in utero embryonic development | 7.01E-07 | 10 |
| BP | GO:0046427 | positive regulation of receptor signaling pathway via JAK-STAT | 7.15E-07 | 6 |
| BP | GO:1903035 | negative regulation of response to wounding | 7.63E-07 | 6 |
| BP | GO:0050769 | positive regulation of neurogenesis | 7.67E-07 | 11 |
| BP | GO:0035094 | response to nicotine | 8.00E-07 | 5 |
| BP | GO:0043124 | negative regulation of I-kappaB kinase/NF-kappaB signaling | 8.00E-07 | 5 |
| BP | GO:1904707 | positive regulation of vascular smooth muscle cell proliferation | 8.00E-07 | 5 |
| BP | GO:0032516 | positive regulation of phosphoprotein phosphatase activity | 8.15E-07 | 4 |
| BP | GO:0045639 | positive regulation of myeloid cell differentiation | 8.15E-07 | 6 |
| BP | GO:0060333 | interferon-gamma-mediated signaling pathway | 8.15E-07 | 6 |
| BP | GO:1904892 | regulation of receptor signaling pathway via STAT | 8.20E-07 | 7 |
| BP | GO:0009410 | response to xenobiotic stimulus | 8.39E-07 | 9 |
| BP | GO:0043524 | negative regulation of neuron apoptotic process | 8.58E-07 | 7 |
| BP | GO:1904894 | positive regulation of receptor signaling pathway via STAT | 8.70E-07 | 6 |
| BP | GO:2000117 | negative regulation of cysteine-type endopeptidase activity | 8.70E-07 | 6 |
| BP | GO:0046854 | phosphatidylinositol phosphorylation | 8.87E-07 | 5 |
| BP | GO:0042692 | muscle cell differentiation | 9.34E-07 | 10 |
| BP | GO:0051092 | positive regulation of NF-kappaB transcription factor activity | 9.40E-07 | 7 |
| BP | GO:0032271 | regulation of protein polymerization | 9.76E-07 | 8 |
| BP | GO:0010869 | regulation of receptor biosynthetic process | 9.93E-07 | 4 |
| BP | GO:0030878 | thyroid gland development | 9.93E-07 | 4 |
| BP | GO:0032928 | regulation of superoxide anion generation | 9.93E-07 | 4 |
| BP | GO:0002699 | positive regulation of immune effector process | 1.01E-06 | 8 |
| BP | GO:0042100 | B cell proliferation | 1.05E-06 | 6 |
| BP | GO:0032755 | positive regulation of interleukin-6 production | 1.12E-06 | 6 |
| BP | GO:0010959 | regulation of metal ion transport | 1.15E-06 | 10 |
| BP | GO:1902882 | regulation of response to oxidative stress | 1.19E-06 | 6 |
| BP | GO:0001503 | ossification | 1.26E-06 | 10 |
| BP | GO:0050848 | regulation of calcium-mediated signaling | 1.26E-06 | 6 |
| BP | GO:2001243 | negative regulation of intrinsic apoptotic signaling pathway | 1.26E-06 | 6 |
| BP | GO:0051353 | positive regulation of oxidoreductase activity | 1.31E-06 | 5 |
| BP | GO:0032102 | negative regulation of response to external stimulus | 1.32E-06 | 10 |
| BP | GO:0043271 | negative regulation of ion transport | 1.34E-06 | 7 |
| BP | GO:0042306 | regulation of protein import into nucleus | 1.44E-06 | 5 |
| BP | GO:0046632 | alpha-beta T cell differentiation | 1.51E-06 | 6 |
| BP | GO:0032680 | regulation of tumor necrosis factor production | 1.52E-06 | 7 |
| BP | GO:0046661 | male sex differentiation | 1.52E-06 | 7 |
| BP | GO:0050808 | synapse organization | 1.58E-06 | 10 |
| BP | GO:0032635 | interleukin-6 production | 1.58E-06 | 7 |
| BP | GO:0002367 | cytokine production involved in immune response | 1.60E-06 | 6 |
| BP | GO:0009755 | hormone-mediated signaling pathway | 1.61E-06 | 8 |
| BP | GO:0071453 | cellular response to oxygen levels | 1.66E-06 | 8 |
| BP | GO:0060330 | regulation of response to interferon-gamma | 1.70E-06 | 4 |
| BP | GO:0060334 | regulation of interferon-gamma-mediated signaling pathway | 1.70E-06 | 4 |
| BP | GO:0032640 | tumor necrosis factor production | 1.72E-06 | 7 |
| BP | GO:0045931 | positive regulation of mitotic cell cycle | 1.72E-06 | 7 |
| BP | GO:1903555 | regulation of tumor necrosis factor superfamily cytokine production | 1.72E-06 | 7 |
| BP | GO:0043122 | regulation of I-kappaB kinase/NF-kappaB signaling | 1.83E-06 | 8 |
| BP | GO:0010876 | lipid localization | 1.84E-06 | 10 |
| BP | GO:0032722 | positive regulation of chemokine production | 1.88E-06 | 5 |
| BP | GO:0035306 | positive regulation of dephosphorylation | 1.88E-06 | 5 |
| BP | GO:1902041 | regulation of extrinsic apoptotic signaling pathway via death domain receptors | 1.88E-06 | 5 |
| BP | GO:1904589 | regulation of protein import | 1.88E-06 | 5 |
| BP | GO:0050663 | cytokine secretion | 2.00E-06 | 8 |
| BP | GO:0030194 | positive regulation of blood coagulation | 2.01E-06 | 4 |
| BP | GO:0048169 | regulation of long-term neuronal synaptic plasticity | 2.01E-06 | 4 |
| BP | GO:1900048 | positive regulation of hemostasis | 2.01E-06 | 4 |
| BP | GO:0035051 | cardiocyte differentiation | 2.02E-06 | 7 |
| BP | GO:0070527 | platelet aggregation | 2.04E-06 | 5 |
| BP | GO:2000351 | regulation of endothelial cell apoptotic process | 2.04E-06 | 5 |
| BP | GO:0071706 | tumor necrosis factor superfamily cytokine production | 2.10E-06 | 7 |
| BP | GO:0030307 | positive regulation of cell growth | 2.19E-06 | 7 |
| BP | GO:0038095 | Fc-epsilon receptor signaling pathway | 2.19E-06 | 7 |
| BP | GO:0046324 | regulation of glucose import | 2.22E-06 | 5 |
| BP | GO:0019318 | hexose metabolic process | 2.27E-06 | 8 |
| BP | GO:0048143 | astrocyte activation | 2.35E-06 | 4 |
| BP | GO:0050820 | positive regulation of coagulation | 2.35E-06 | 4 |
| BP | GO:0071868 | cellular response to monoamine stimulus | 2.36E-06 | 6 |
| BP | GO:0071870 | cellular response to catecholamine stimulus | 2.36E-06 | 6 |
| BP | GO:1905953 | negative regulation of lipid localization | 2.42E-06 | 5 |
| BP | GO:0006644 | phospholipid metabolic process | 2.52E-06 | 10 |
| BP | GO:0051251 | positive regulation of lymphocyte activation | 2.54E-06 | 9 |
| BP | GO:0001659 | temperature homeostasis | 2.55E-06 | 7 |
| BP | GO:0072676 | lymphocyte migration | 2.62E-06 | 6 |
| BP | GO:0002260 | lymphocyte homeostasis | 2.62E-06 | 5 |
| BP | GO:0045408 | regulation of interleukin-6 biosynthetic process | 2.74E-06 | 4 |
| BP | GO:0006816 | calcium ion transport | 2.74E-06 | 10 |
| BP | GO:0001938 | positive regulation of endothelial cell proliferation | 2.76E-06 | 6 |
| BP | GO:0071867 | response to monoamine | 2.91E-06 | 6 |
| BP | GO:0071869 | response to catecholamine | 2.91E-06 | 6 |
| BP | GO:0031330 | negative regulation of cellular catabolic process | 2.97E-06 | 8 |
| BP | GO:0060249 | anatomical structure homeostasis | 3.03E-06 | 10 |
| BP | GO:0043550 | regulation of lipid kinase activity | 3.07E-06 | 5 |
| BP | GO:0046834 | lipid phosphorylation | 3.07E-06 | 5 |
| BP | GO:0032956 | regulation of actin cytoskeleton organization | 3.16E-06 | 9 |
| BP | GO:0042226 | interleukin-6 biosynthetic process | 3.16E-06 | 4 |
| BP | GO:0010821 | regulation of mitochondrion organization | 3.20E-06 | 7 |
| BP | GO:2001235 | positive regulation of apoptotic signaling pathway | 3.20E-06 | 7 |
| BP | GO:0032963 | collagen metabolic process | 3.22E-06 | 6 |
| BP | GO:1904375 | regulation of protein localization to cell periphery | 3.22E-06 | 6 |
| BP | GO:0032729 | positive regulation of interferon-gamma production | 3.32E-06 | 5 |
| BP | GO:0072577 | endothelial cell apoptotic process | 3.32E-06 | 5 |
| BP | GO:0072678 | T cell migration | 3.32E-06 | 5 |
| BP | GO:0010565 | regulation of cellular ketone metabolic process | 3.32E-06 | 7 |
| BP | GO:0033673 | negative regulation of kinase activity | 3.33E-06 | 8 |
| BP | GO:0002285 | lymphocyte activation involved in immune response | 3.45E-06 | 7 |
| BP | GO:0043401 | steroid hormone mediated signaling pathway | 3.57E-06 | 7 |
| BP | GO:0046320 | regulation of fatty acid oxidation | 3.64E-06 | 4 |
| BP | GO:1902903 | regulation of supramolecular fiber organization | 4.00E-06 | 9 |
| BP | GO:0019915 | lipid storage | 4.15E-06 | 5 |
| BP | GO:0033627 | cell adhesion mediated by integrin | 4.15E-06 | 5 |
| BP | GO:0035924 | cellular response to vascular endothelial growth factor stimulus | 4.15E-06 | 5 |
| BP | GO:0046323 | glucose import | 4.15E-06 | 5 |
| BP | GO:0043457 | regulation of cellular respiration | 4.17E-06 | 4 |
| BP | GO:0045648 | positive regulation of erythrocyte differentiation | 4.17E-06 | 4 |
| BP | GO:0007249 | I-kappaB kinase/NF-kappaB signaling | 4.67E-06 | 8 |
| BP | GO:0045907 | positive regulation of vasoconstriction | 4.75E-06 | 4 |
| BP | GO:0055094 | response to lipoprotein particle | 4.75E-06 | 4 |
| BP | GO:0002460 | adaptive immune response based on somatic recombination of immune receptors built from immunoglobulin superfamily domains | 4.79E-06 | 9 |
| BP | GO:0042594 | response to starvation | 4.91E-06 | 7 |
| BP | GO:0071478 | cellular response to radiation | 4.91E-06 | 7 |
| BP | GO:0032070 | regulation of deoxyribonuclease activity | 4.97E-06 | 3 |
| BP | GO:0045348 | positive regulation of MHC class II biosynthetic process | 4.97E-06 | 3 |
| BP | GO:0051918 | negative regulation of fibrinolysis | 4.97E-06 | 3 |
| BP | GO:0071104 | response to interleukin-9 | 4.97E-06 | 3 |
| BP | GO:0043112 | receptor metabolic process | 5.09E-06 | 7 |
| BP | GO:0042531 | positive regulation of tyrosine phosphorylation of STAT protein | 5.15E-06 | 5 |
| BP | GO:0071479 | cellular response to ionizing radiation | 5.15E-06 | 5 |
| BP | GO:0032368 | regulation of lipid transport | 5.22E-06 | 6 |
| BP | GO:0007219 | Notch signaling pathway | 5.26E-06 | 7 |
| BP | GO:1901654 | response to ketone | 5.26E-06 | 7 |
| BP | GO:2000352 | negative regulation of endothelial cell apoptotic process | 5.40E-06 | 4 |
| BP | GO:0035270 | endocrine system development | 5.72E-06 | 6 |
| BP | GO:0002685 | regulation of leukocyte migration | 5.82E-06 | 7 |
| BP | GO:0043627 | response to estrogen | 5.90E-06 | 5 |
| BP | GO:0002687 | positive regulation of leukocyte migration | 5.99E-06 | 6 |
| BP | GO:0009749 | response to glucose | 6.02E-06 | 7 |
| BP | GO:0010922 | positive regulation of phosphatase activity | 6.10E-06 | 4 |
| BP | GO:0042533 | tumor necrosis factor biosynthetic process | 6.10E-06 | 4 |
| BP | GO:0042534 | regulation of tumor necrosis factor biosynthetic process | 6.10E-06 | 4 |
| BP | GO:0043276 | anoikis | 6.10E-06 | 4 |
| BP | GO:0043368 | positive T cell selection | 6.10E-06 | 4 |
| BP | GO:0071402 | cellular response to lipoprotein particle stimulus | 6.10E-06 | 4 |
| BP | GO:0060485 | mesenchyme development | 6.10E-06 | 8 |
| BP | GO:0046890 | regulation of lipid biosynthetic process | 6.23E-06 | 7 |
| BP | GO:0032677 | regulation of interleukin-8 production | 6.31E-06 | 5 |
| BP | GO:0070838 | divalent metal ion transport | 6.55E-06 | 10 |
| BP | GO:0060047 | heart contraction | 6.60E-06 | 8 |
| BP | GO:1903201 | regulation of oxidative stress-induced cell death | 6.75E-06 | 5 |
| BP | GO:0060439 | trachea morphogenesis | 6.82E-06 | 3 |
| BP | GO:0030183 | B cell differentiation | 6.84E-06 | 6 |
| BP | GO:0001893 | maternal placenta development | 6.87E-06 | 4 |
| BP | GO:0002696 | positive regulation of leukocyte activation | 7.25E-06 | 9 |
| BP | GO:0006869 | lipid transport | 7.25E-06 | 9 |
| BP | GO:0002440 | production of molecular mediator of immune response | 7.31E-06 | 8 |
| BP | GO:0072511 | divalent inorganic cation transport | 7.44E-06 | 10 |
| BP | GO:0071333 | cellular response to glucose stimulus | 7.47E-06 | 6 |
| BP | GO:0005996 | monosaccharide metabolic process | 7.50E-06 | 8 |
| BP | GO:0034764 | positive regulation of transmembrane transport | 7.57E-06 | 7 |
| BP | GO:0010742 | macrophage derived foam cell differentiation | 7.70E-06 | 4 |
| BP | GO:0042554 | superoxide anion generation | 7.70E-06 | 4 |
| BP | GO:0090077 | foam cell differentiation | 7.70E-06 | 4 |
| BP | GO:0090218 | positive regulation of lipid kinase activity | 7.70E-06 | 4 |
| BP | GO:0031346 | positive regulation of cell projection organization | 7.72E-06 | 9 |
| BP | GO:0002703 | regulation of leukocyte mediated immunity | 7.82E-06 | 7 |
| BP | GO:0050921 | positive regulation of chemotaxis | 8.14E-06 | 6 |
| BP | GO:0071331 | cellular response to hexose stimulus | 8.14E-06 | 6 |
| BP | GO:0010827 | regulation of glucose transmembrane transport | 8.19E-06 | 5 |
| BP | GO:0002064 | epithelial cell development | 8.33E-06 | 7 |
| BP | GO:0071456 | cellular response to hypoxia | 8.33E-06 | 7 |
| BP | GO:0071326 | cellular response to monosaccharide stimulus | 8.49E-06 | 6 |
| BP | GO:0003015 | heart process | 8.51E-06 | 8 |
| BP | GO:0032970 | regulation of actin filament-based process | 8.57E-06 | 9 |
| BP | GO:0050670 | regulation of lymphocyte proliferation | 8.59E-06 | 7 |
| BP | GO:0021587 | cerebellum morphogenesis | 8.62E-06 | 4 |
| BP | GO:0034405 | response to fluid shear stress | 8.62E-06 | 4 |
| BP | GO:0010675 | regulation of cellular carbohydrate metabolic process | 8.85E-06 | 6 |
| BP | GO:0032944 | regulation of mononuclear cell proliferation | 8.87E-06 | 7 |
| BP | GO:0038110 | interleukin-2-mediated signaling pathway | 9.07E-06 | 3 |
| BP | GO:0045416 | positive regulation of interleukin-8 biosynthetic process | 9.07E-06 | 3 |
| BP | GO:0050707 | regulation of cytokine secretion | 9.15E-06 | 7 |
| BP | GO:0097529 | myeloid leukocyte migration | 9.15E-06 | 7 |
| BP | GO:0010975 | regulation of neuron projection development | 9.37E-06 | 10 |
| BP | GO:0046326 | positive regulation of glucose import | 9.60E-06 | 4 |
| BP | GO:0048009 | insulin-like growth factor receptor signaling pathway | 9.60E-06 | 4 |
| BP | GO:0060416 | response to growth hormone | 9.60E-06 | 4 |
| BP | GO:0002700 | regulation of production of molecular mediator of immune response | 9.62E-06 | 6 |
| BP | GO:0035304 | regulation of protein dephosphorylation | 9.62E-06 | 6 |
| BP | GO:0045580 | regulation of T cell differentiation | 9.62E-06 | 6 |
| BP | GO:0060968 | regulation of gene silencing | 9.62E-06 | 6 |
| BP | GO:0034109 | homotypic cell-cell adhesion | 9.85E-06 | 5 |
| BP | GO:0032642 | regulation of chemokine production | 1.05E-05 | 5 |
| BP | GO:0010810 | regulation of cell-substrate adhesion | 1.07E-05 | 7 |
| BP | GO:0042509 | regulation of tyrosine phosphorylation of STAT protein | 1.11E-05 | 5 |
| BP | GO:0048145 | regulation of fibroblast proliferation | 1.11E-05 | 5 |
| BP | GO:2000106 | regulation of leukocyte apoptotic process | 1.11E-05 | 5 |
| BP | GO:0030879 | mammary gland development | 1.13E-05 | 6 |
| BP | GO:0036294 | cellular response to decreased oxygen levels | 1.13E-05 | 7 |
| BP | GO:0050920 | regulation of chemotaxis | 1.13E-05 | 7 |
| BP | GO:0050807 | regulation of synapse organization | 1.17E-05 | 7 |
| BP | GO:0071352 | cellular response to interleukin-2 | 1.18E-05 | 3 |
| BP | GO:0071322 | cellular response to carbohydrate stimulus | 1.18E-05 | 6 |
| BP | GO:1902107 | positive regulation of leukocyte differentiation | 1.18E-05 | 6 |
| BP | GO:0046889 | positive regulation of lipid biosynthetic process | 1.18E-05 | 5 |
| BP | GO:0048144 | fibroblast proliferation | 1.18E-05 | 5 |
| BP | GO:1900371 | regulation of purine nucleotide biosynthetic process | 1.18E-05 | 4 |
| BP | GO:0051651 | maintenance of location in cell | 1.20E-05 | 7 |
| BP | GO:0007612 | learning | 1.22E-05 | 6 |
| BP | GO:0035264 | multicellular organism growth | 1.27E-05 | 6 |
| BP | GO:0051384 | response to glucocorticoid | 1.27E-05 | 6 |
| BP | GO:0021575 | hindbrain morphogenesis | 1.31E-05 | 4 |
| BP | GO:0030808 | regulation of nucleotide biosynthetic process | 1.31E-05 | 4 |
| BP | GO:0050873 | brown fat cell differentiation | 1.31E-05 | 4 |
| BP | GO:0150077 | regulation of neuroinflammatory response | 1.31E-05 | 4 |
| BP | GO:1902893 | regulation of pri-miRNA transcription by RNA polymerase II | 1.31E-05 | 4 |
| BP | GO:0070663 | regulation of leukocyte proliferation | 1.31E-05 | 7 |
| BP | GO:0007260 | tyrosine phosphorylation of STAT protein | 1.32E-05 | 5 |
| BP | GO:0048013 | ephrin receptor signaling pathway | 1.32E-05 | 5 |
| BP | GO:0060291 | long-term synaptic potentiation | 1.32E-05 | 5 |
| BP | GO:0071236 | cellular response to antibiotic | 1.32E-05 | 6 |
| BP | GO:0045444 | fat cell differentiation | 1.35E-05 | 7 |
| BP | GO:0030595 | leukocyte chemotaxis | 1.39E-05 | 7 |
| BP | GO:0009267 | cellular response to starvation | 1.43E-05 | 6 |
| BP | GO:0014074 | response to purine-containing compound | 1.43E-05 | 6 |
| BP | GO:0046486 | glycerolipid metabolic process | 1.44E-05 | 9 |
| BP | GO:1903725 | regulation of phospholipid metabolic process | 1.48E-05 | 5 |
| BP | GO:0045346 | regulation of MHC class II biosynthetic process | 1.49E-05 | 3 |
| BP | GO:0048308 | organelle inheritance | 1.49E-05 | 3 |
| BP | GO:0048313 | Golgi inheritance | 1.49E-05 | 3 |
| BP | GO:0051712 | positive regulation of killing of cells of other organism | 1.49E-05 | 3 |
| BP | GO:0051917 | regulation of fibrinolysis | 1.49E-05 | 3 |
| BP | GO:0070669 | response to interleukin-2 | 1.49E-05 | 3 |
| BP | GO:1905050 | positive regulation of metallopeptidase activity | 1.49E-05 | 3 |
| BP | GO:0050803 | regulation of synapse structure or activity | 1.52E-05 | 7 |
| BP | GO:0032602 | chemokine production | 1.56E-05 | 5 |
| BP | GO:0034198 | cellular response to amino acid starvation | 1.58E-05 | 4 |
| BP | GO:0071364 | cellular response to epidermal growth factor stimulus | 1.58E-05 | 4 |
| BP | GO:1904646 | cellular response to amyloid-beta | 1.58E-05 | 4 |
| BP | GO:0032675 | regulation of interleukin-6 production | 1.60E-05 | 6 |
| BP | GO:0043255 | regulation of carbohydrate biosynthetic process | 1.65E-05 | 5 |
| BP | GO:0060562 | epithelial tube morphogenesis | 1.73E-05 | 8 |
| BP | GO:0006417 | regulation of translation | 1.74E-05 | 9 |
| BP | GO:0010828 | positive regulation of glucose transmembrane transport | 1.74E-05 | 4 |
| BP | GO:0030225 | macrophage differentiation | 1.74E-05 | 4 |
| BP | GO:0042129 | regulation of T cell proliferation | 1.86E-05 | 6 |
| BP | GO:0030730 | sequestering of triglyceride | 1.86E-05 | 3 |
| BP | GO:0045342 | MHC class II biosynthetic process | 1.86E-05 | 3 |
| BP | GO:1903358 | regulation of Golgi organization | 1.86E-05 | 3 |
| BP | GO:0006469 | negative regulation of protein kinase activity | 1.90E-05 | 7 |
| BP | GO:1901184 | regulation of ERBB signaling pathway | 1.93E-05 | 5 |
| BP | GO:0046578 | regulation of Ras protein signal transduction | 2.06E-05 | 7 |
| BP | GO:0002673 | regulation of acute inflammatory response | 2.08E-05 | 4 |
| BP | GO:0033628 | regulation of cell adhesion mediated by integrin | 2.08E-05 | 4 |
| BP | GO:0060711 | labyrinthine layer development | 2.08E-05 | 4 |
| BP | GO:0071675 | regulation of mononuclear cell migration | 2.08E-05 | 4 |
| BP | GO:1900015 | regulation of cytokine production involved in inflammatory response | 2.08E-05 | 4 |
| BP | GO:1990928 | response to amino acid starvation | 2.08E-05 | 4 |
| BP | GO:0008202 | steroid metabolic process | 2.11E-05 | 8 |
| BP | GO:0002683 | negative regulation of immune system process | 2.13E-05 | 9 |
| BP | GO:0002702 | positive regulation of production of molecular mediator of immune response | 2.15E-05 | 5 |
| BP | GO:0044070 | regulation of anion transport | 2.15E-05 | 5 |
| BP | GO:1903076 | regulation of protein localization to plasma membrane | 2.15E-05 | 5 |
| BP | GO:0016042 | lipid catabolic process | 2.20E-05 | 8 |
| BP | GO:0006953 | acute-phase response | 2.27E-05 | 4 |
| BP | GO:0043303 | mast cell degranulation | 2.27E-05 | 4 |
| BP | GO:0045646 | regulation of erythrocyte differentiation | 2.27E-05 | 4 |
| BP | GO:0048806 | genitalia development | 2.27E-05 | 4 |
| BP | GO:0002739 | regulation of cytokine secretion involved in immune response | 2.29E-05 | 3 |
| BP | GO:0010885 | regulation of cholesterol storage | 2.29E-05 | 3 |
| BP | GO:1902004 | positive regulation of amyloid-beta formation | 2.29E-05 | 3 |
| BP | GO:1990000 | amyloid fibril formation | 2.29E-05 | 3 |
| BP | GO:0031960 | response to corticosteroid | 2.30E-05 | 6 |
| BP | GO:0015908 | fatty acid transport | 2.37E-05 | 5 |
| BP | GO:0030316 | osteoclast differentiation | 2.37E-05 | 5 |
| BP | GO:0002279 | mast cell activation involved in immune response | 2.47E-05 | 4 |
| BP | GO:0008542 | visual learning | 2.47E-05 | 4 |
| BP | GO:0051972 | regulation of telomerase activity | 2.47E-05 | 4 |
| BP | GO:1903727 | positive regulation of phospholipid metabolic process | 2.47E-05 | 4 |
| BP | GO:0031341 | regulation of cell killing | 2.49E-05 | 5 |
| BP | GO:0042180 | cellular ketone metabolic process | 2.54E-05 | 7 |
| BP | GO:2001242 | regulation of intrinsic apoptotic signaling pathway | 2.55E-05 | 6 |
| BP | GO:0019395 | fatty acid oxidation | 2.62E-05 | 5 |
| BP | GO:0070301 | cellular response to hydrogen peroxide | 2.62E-05 | 5 |
| BP | GO:0002448 | mast cell mediated immunity | 2.68E-05 | 4 |
| BP | GO:0002534 | cytokine production involved in inflammatory response | 2.68E-05 | 4 |
| BP | GO:0032757 | positive regulation of interleukin-8 production | 2.68E-05 | 4 |
| BP | GO:0051709 | regulation of killing of cells of other organism | 2.77E-05 | 3 |
| BP | GO:0061298 | retina vasculature development in camera-type eye | 2.77E-05 | 3 |
| BP | GO:2000641 | regulation of early endosome to late endosome transport | 2.77E-05 | 3 |
| BP | GO:2001267 | regulation of cysteine-type endopeptidase activity involved in apoptotic signaling pathway | 2.77E-05 | 3 |
| BP | GO:0030072 | peptide hormone secretion | 2.82E-05 | 7 |
| BP | GO:0001906 | cell killing | 2.82E-05 | 6 |
| BP | GO:0032649 | regulation of interferon-gamma production | 2.88E-05 | 5 |
| BP | GO:0034440 | lipid oxidation | 2.88E-05 | 5 |
| BP | GO:0031113 | regulation of microtubule polymerization | 2.90E-05 | 4 |
| BP | GO:0060425 | lung morphogenesis | 2.90E-05 | 4 |
| BP | GO:0045619 | regulation of lymphocyte differentiation | 2.92E-05 | 6 |
| BP | GO:0050728 | negative regulation of inflammatory response | 2.92E-05 | 6 |
| BP | GO:0048146 | positive regulation of fibroblast proliferation | 3.14E-05 | 4 |
| BP | GO:0010878 | cholesterol storage | 3.31E-05 | 3 |
| BP | GO:0031998 | regulation of fatty acid beta-oxidation | 3.31E-05 | 3 |
| BP | GO:0060749 | mammary gland alveolus development | 3.31E-05 | 3 |
| BP | GO:0061377 | mammary gland lobule development | 3.31E-05 | 3 |
| BP | GO:0019233 | sensory perception of pain | 3.32E-05 | 5 |
| BP | GO:0032731 | positive regulation of interleukin-1 beta production | 3.39E-05 | 4 |
| BP | GO:0048168 | regulation of neuronal synaptic plasticity | 3.39E-05 | 4 |
| BP | GO:0046488 | phosphatidylinositol metabolic process | 3.44E-05 | 6 |
| BP | GO:0008593 | regulation of Notch signaling pathway | 3.48E-05 | 5 |
| BP | GO:0090257 | regulation of muscle system process | 3.53E-05 | 7 |
| BP | GO:0007632 | visual behavior | 3.66E-05 | 4 |
| BP | GO:0010888 | negative regulation of lipid storage | 3.93E-05 | 3 |
| BP | GO:0045414 | regulation of interleukin-8 biosynthetic process | 3.93E-05 | 3 |
| BP | GO:0046641 | positive regulation of alpha-beta T cell proliferation | 3.93E-05 | 3 |
| BP | GO:0060438 | trachea development | 3.93E-05 | 3 |
| BP | GO:1902176 | negative regulation of oxidative stress-induced intrinsic apoptotic signaling pathway | 3.93E-05 | 3 |
| BP | GO:1903798 | regulation of production of miRNAs involved in gene silencing by miRNA | 3.93E-05 | 3 |
| BP | GO:1904659 | glucose transmembrane transport | 3.98E-05 | 5 |
| BP | GO:2000278 | regulation of DNA biosynthetic process | 3.98E-05 | 5 |
| BP | GO:0051099 | positive regulation of binding | 4.03E-05 | 6 |
| BP | GO:1905330 | regulation of morphogenesis of an epithelium | 4.16E-05 | 6 |
| BP | GO:0051149 | positive regulation of muscle cell differentiation | 4.16E-05 | 5 |
| BP | GO:0042220 | response to cocaine | 4.24E-05 | 4 |
| BP | GO:0043551 | regulation of phosphatidylinositol 3-kinase activity | 4.24E-05 | 4 |
| BP | GO:0046456 | icosanoid biosynthetic process | 4.24E-05 | 4 |
| BP | GO:0051147 | regulation of muscle cell differentiation | 4.29E-05 | 6 |
| BP | GO:0008217 | regulation of blood pressure | 4.42E-05 | 6 |
| BP | GO:0010332 | response to gamma radiation | 4.56E-05 | 4 |
| BP | GO:0031295 | T cell costimulation | 4.56E-05 | 4 |
| BP | GO:0032615 | interleukin-12 production | 4.56E-05 | 4 |
| BP | GO:0060688 | regulation of morphogenesis of a branching structure | 4.56E-05 | 4 |
| BP | GO:1903078 | positive regulation of protein localization to plasma membrane | 4.56E-05 | 4 |
| BP | GO:1903202 | negative regulation of oxidative stress-induced cell death | 4.56E-05 | 4 |
| BP | GO:0002374 | cytokine secretion involved in immune response | 4.61E-05 | 3 |
| BP | GO:0010042 | response to manganese ion | 4.61E-05 | 3 |
| BP | GO:0042228 | interleukin-8 biosynthetic process | 4.61E-05 | 3 |
| BP | GO:1902993 | positive regulation of amyloid precursor protein catabolic process | 4.61E-05 | 3 |
| BP | GO:0043409 | negative regulation of MAPK cascade | 4.70E-05 | 6 |
| BP | GO:0008645 | hexose transmembrane transport | 4.74E-05 | 5 |
| BP | GO:0034765 | regulation of ion transmembrane transport | 4.82E-05 | 9 |
| BP | GO:0031294 | lymphocyte costimulation | 4.89E-05 | 4 |
| BP | GO:1900408 | negative regulation of cellular response to oxidative stress | 4.89E-05 | 4 |
| BP | GO:0002223 | stimulatory C-type lectin receptor signaling pathway | 4.95E-05 | 5 |
| BP | GO:0006096 | glycolytic process | 4.95E-05 | 5 |
| BP | GO:0032609 | interferon-gamma production | 4.95E-05 | 5 |
| BP | GO:0046620 | regulation of organ growth | 4.95E-05 | 5 |
| BP | GO:0060964 | regulation of gene silencing by miRNA | 4.95E-05 | 5 |
| BP | GO:0006690 | icosanoid metabolic process | 5.16E-05 | 5 |
| BP | GO:0006757 | ATP generation from ADP | 5.16E-05 | 5 |
| BP | GO:0015749 | monosaccharide transmembrane transport | 5.16E-05 | 5 |
| BP | GO:0010676 | positive regulation of cellular carbohydrate metabolic process | 5.23E-05 | 4 |
| BP | GO:0019229 | regulation of vasoconstriction | 5.23E-05 | 4 |
| BP | GO:0030728 | ovulation | 5.36E-05 | 3 |
| BP | GO:0035162 | embryonic hemopoiesis | 5.36E-05 | 3 |
| BP | GO:0070920 | regulation of production of small RNA involved in gene silencing by RNA | 5.36E-05 | 3 |
| BP | GO:0032732 | positive regulation of interleukin-1 production | 5.60E-05 | 4 |
| BP | GO:1902883 | negative regulation of response to oxidative stress | 5.60E-05 | 4 |
| BP | GO:0002220 | innate immune response activating cell surface receptor signaling pathway | 5.61E-05 | 5 |
| BP | GO:0006661 | phosphatidylinositol biosynthetic process | 5.61E-05 | 5 |
| BP | GO:0010822 | positive regulation of mitochondrion organization | 5.61E-05 | 5 |
| BP | GO:0034219 | carbohydrate transmembrane transport | 5.61E-05 | 5 |
| BP | GO:0042176 | regulation of protein catabolic process | 5.71E-05 | 8 |
| BP | GO:0002758 | innate immune response-activating signal transduction | 5.84E-05 | 5 |
| BP | GO:0002761 | regulation of myeloid leukocyte differentiation | 5.84E-05 | 5 |
| BP | GO:0043666 | regulation of phosphoprotein phosphatase activity | 5.84E-05 | 5 |
| BP | GO:0060147 | regulation of posttranscriptional gene silencing | 5.84E-05 | 5 |
| BP | GO:0060966 | regulation of gene silencing by RNA | 5.84E-05 | 5 |
| BP | GO:0010976 | positive regulation of neuron projection development | 5.92E-05 | 7 |
| BP | GO:0045576 | mast cell activation | 5.98E-05 | 4 |
| BP | GO:0006925 | inflammatory cell apoptotic process | 6.19E-05 | 3 |
| BP | GO:0032069 | regulation of nuclease activity | 6.19E-05 | 3 |
| BP | GO:0035584 | calcium-mediated signaling using intracellular calcium source | 6.19E-05 | 3 |
| BP | GO:0051000 | positive regulation of nitric-oxide synthase activity | 6.19E-05 | 3 |
| BP | GO:0071379 | cellular response to prostaglandin stimulus | 6.19E-05 | 3 |
| BP | GO:0001952 | regulation of cell-matrix adhesion | 6.33E-05 | 5 |
| BP | GO:0030888 | regulation of B cell proliferation | 6.39E-05 | 4 |
| BP | GO:0045123 | cellular extravasation | 6.39E-05 | 4 |
| BP | GO:0010508 | positive regulation of autophagy | 6.59E-05 | 5 |
| BP | GO:0045787 | positive regulation of cell cycle | 6.61E-05 | 8 |
| BP | GO:0071897 | DNA biosynthetic process | 6.67E-05 | 6 |
| BP | GO:0031640 | killing of cells of other organism | 6.81E-05 | 4 |
| BP | GO:0051205 | protein insertion into membrane | 6.81E-05 | 4 |
| BP | GO:1903578 | regulation of ATP metabolic process | 6.86E-05 | 5 |
| BP | GO:0030810 | positive regulation of nucleotide biosynthetic process | 7.10E-05 | 3 |
| BP | GO:0050995 | negative regulation of lipid catabolic process | 7.10E-05 | 3 |
| BP | GO:1900373 | positive regulation of purine nucleotide biosynthetic process | 7.10E-05 | 3 |
| BP | GO:2000637 | positive regulation of gene silencing by miRNA | 7.10E-05 | 3 |
| BP | GO:0046031 | ADP metabolic process | 7.13E-05 | 5 |
| BP | GO:0046622 | positive regulation of organ growth | 7.25E-05 | 4 |
| BP | GO:1904377 | positive regulation of protein localization to cell periphery | 7.25E-05 | 4 |
| BP | GO:0031032 | actomyosin structure organization | 7.26E-05 | 6 |
| BP | GO:0071621 | granulocyte chemotaxis | 7.41E-05 | 5 |
| BP | GO:0051146 | striated muscle cell differentiation | 7.70E-05 | 7 |
| BP | GO:0051209 | release of sequestered calcium ion into cytosol | 7.70E-05 | 5 |
| BP | GO:0045600 | positive regulation of fat cell differentiation | 7.72E-05 | 4 |
| BP | GO:0045670 | regulation of osteoclast differentiation | 7.72E-05 | 4 |
| BP | GO:0046579 | positive regulation of Ras protein signal transduction | 7.72E-05 | 4 |
| BP | GO:0048645 | animal organ formation | 7.72E-05 | 4 |
| BP | GO:1903672 | positive regulation of sprouting angiogenesis | 7.72E-05 | 4 |
| BP | GO:0031929 | TOR signaling | 8.00E-05 | 5 |
| BP | GO:0010288 | response to lead ion | 8.10E-05 | 3 |
| BP | GO:0046697 | decidualization | 8.10E-05 | 3 |
| BP | GO:0060148 | positive regulation of posttranscriptional gene silencing | 8.10E-05 | 3 |
| BP | GO:0060396 | growth hormone receptor signaling pathway | 8.10E-05 | 3 |
| BP | GO:1903649 | regulation of cytoplasmic transport | 8.10E-05 | 3 |
| BP | GO:0030278 | regulation of ossification | 8.10E-05 | 6 |
| BP | GO:0051283 | negative regulation of sequestering of calcium ion | 8.31E-05 | 5 |
| BP | GO:1902905 | positive regulation of supramolecular fiber organization | 8.33E-05 | 6 |
| BP | GO:0030100 | regulation of endocytosis | 8.55E-05 | 6 |
| BP | GO:0045727 | positive regulation of translation | 8.63E-05 | 5 |
| BP | GO:0046626 | regulation of insulin receptor signaling pathway | 8.71E-05 | 4 |
| BP | GO:0051282 | regulation of sequestering of calcium ion | 8.95E-05 | 5 |
| BP | GO:0030073 | insulin secretion | 9.02E-05 | 6 |
| BP | GO:0060571 | morphogenesis of an epithelial fold | 9.18E-05 | 3 |
| BP | GO:0071378 | cellular response to growth hormone stimulus | 9.18E-05 | 3 |
| BP | GO:2000573 | positive regulation of DNA biosynthetic process | 9.23E-05 | 4 |
| BP | GO:0016051 | carbohydrate biosynthetic process | 9.27E-05 | 6 |
| BP | GO:0090276 | regulation of peptide hormone secretion | 9.27E-05 | 6 |
| BP | GO:0050853 | B cell receptor signaling pathway | 9.29E-05 | 5 |
| BP | GO:1903038 | negative regulation of leukocyte cell-cell adhesion | 9.29E-05 | 5 |
| BP | GO:0034329 | cell junction assembly | 9.37E-05 | 8 |
| BP | GO:0035303 | regulation of dephosphorylation | 9.51E-05 | 6 |
| BP | GO:0006638 | neutral lipid metabolic process | 9.64E-05 | 5 |
| BP | GO:0030518 | intracellular steroid hormone receptor signaling pathway | 9.64E-05 | 5 |
| BP | GO:0050671 | positive regulation of lymphocyte proliferation | 9.64E-05 | 5 |
| BP | GO:0016239 | positive regulation of macroautophagy | 9.79E-05 | 4 |
| BP | GO:0031343 | positive regulation of cell killing | 9.79E-05 | 4 |
| BP | GO:0032946 | positive regulation of mononuclear cell proliferation | 9.99E-05 | 5 |
| BP | GO:0051208 | sequestering of calcium ion | 9.99E-05 | 5 |
| BP | GO:1903579 | negative regulation of ATP metabolic process | 0.000103506 | 3 |
| BP | GO:1905048 | regulation of metallopeptidase activity | 0.000103506 | 3 |
| BP | GO:0006165 | nucleoside diphosphate phosphorylation | 0.000103561 | 5 |
| BP | GO:0046879 | hormone secretion | 0.000105146 | 7 |
| BP | GO:2001020 | regulation of response to DNA damage stimulus | 0.000108323 | 6 |
| BP | GO:0051966 | regulation of synaptic transmission, glutamatergic | 0.000109588 | 4 |
| BP | GO:0007006 | mitochondrial membrane organization | 0.000111172 | 5 |
| BP | GO:0009135 | purine nucleoside diphosphate metabolic process | 0.000111172 | 5 |
| BP | GO:0009179 | purine ribonucleoside diphosphate metabolic process | 0.000111172 | 5 |
| BP | GO:0032273 | positive regulation of protein polymerization | 0.000111172 | 5 |
| BP | GO:0046683 | response to organophosphorus | 0.000111172 | 5 |
| BP | GO:0046939 | nucleotide phosphorylation | 0.000111172 | 5 |
| BP | GO:0048565 | digestive tract development | 0.000111172 | 5 |
| BP | GO:0051057 | positive regulation of small GTPase mediated signal transduction | 0.000115823 | 4 |
| BP | GO:1902175 | regulation of oxidative stress-induced intrinsic apoptotic signaling pathway | 0.000116151 | 3 |
| BP | GO:0044242 | cellular lipid catabolic process | 0.000116907 | 6 |
| BP | GO:0002705 | positive regulation of leukocyte mediated immunity | 0.000119205 | 5 |
| BP | GO:0009185 | ribonucleoside diphosphate metabolic process | 0.000119205 | 5 |
| BP | GO:0038034 | signal transduction in absence of ligand | 0.000122312 | 4 |
| BP | GO:0051881 | regulation of mitochondrial membrane potential | 0.000122312 | 4 |
| BP | GO:0097192 | extrinsic apoptotic signaling pathway in absence of ligand | 0.000122312 | 4 |
| BP | GO:0007584 | response to nutrient | 0.000122924 | 6 |
| BP | GO:0072655 | establishment of protein localization to mitochondrion | 0.000123385 | 5 |
| BP | GO:0009914 | hormone transport | 0.000125714 | 7 |
| BP | GO:0002312 | B cell activation involved in immune response | 0.00012906 | 4 |
| BP | GO:1903524 | positive regulation of blood circulation | 0.00012906 | 4 |
| BP | GO:0001516 | prostaglandin biosynthetic process | 0.000129761 | 3 |
| BP | GO:0042730 | fibrinolysis | 0.000129761 | 3 |
| BP | GO:0046457 | prostanoid biosynthetic process | 0.000129761 | 3 |
| BP | GO:1902003 | regulation of amyloid-beta formation | 0.000129761 | 3 |
| BP | GO:2000108 | positive regulation of leukocyte apoptotic process | 0.000129761 | 3 |
| BP | GO:0070665 | positive regulation of leukocyte proliferation | 0.000132082 | 5 |
| BP | GO:0006470 | protein dephosphorylation | 0.000135849 | 7 |
| BP | GO:0032418 | lysosome localization | 0.000136074 | 4 |
| BP | GO:1900076 | regulation of cellular response to insulin stimulus | 0.000136074 | 4 |
| BP | GO:0046330 | positive regulation of JNK cascade | 0.000136603 | 5 |
| BP | GO:0097553 | calcium ion transmembrane import into cytosol | 0.000136603 | 5 |
| BP | GO:0006163 | purine nucleotide metabolic process | 0.000136674 | 8 |
| BP | GO:0070585 | protein localization to mitochondrion | 0.000141241 | 5 |
| BP | GO:0097530 | granulocyte migration | 0.000141241 | 5 |
| BP | GO:0007263 | nitric oxide mediated signal transduction | 0.000144368 | 3 |
| BP | GO:0033032 | regulation of myeloid cell apoptotic process | 0.000144368 | 3 |
| BP | GO:0045822 | negative regulation of heart contraction | 0.000144368 | 3 |
| BP | GO:0051495 | positive regulation of cytoskeleton organization | 0.000145948 | 6 |
| BP | GO:0002218 | activation of innate immune response | 0.000145999 | 5 |
| BP | GO:0035296 | regulation of tube diameter | 0.000150879 | 5 |
| BP | GO:0097746 | regulation of blood vessel diameter | 0.000150879 | 5 |
| BP | GO:0042310 | vasoconstriction | 0.000150925 | 4 |
| BP | GO:0046785 | microtubule polymerization | 0.000150925 | 4 |
| BP | GO:0035150 | regulation of tube size | 0.000155883 | 5 |
| BP | GO:0001894 | tissue homeostasis | 0.000156805 | 6 |
| BP | GO:0006110 | regulation of glycolytic process | 0.000158774 | 4 |
| BP | GO:0032481 | positive regulation of type I interferon production | 0.000158774 | 4 |
| BP | GO:1901224 | positive regulation of NIK/NF-kappaB signaling | 0.000158774 | 4 |
| BP | GO:0001782 | B cell homeostasis | 0.000160004 | 3 |
| BP | GO:0010743 | regulation of macrophage derived foam cell differentiation | 0.000160004 | 3 |
| BP | GO:0046640 | regulation of alpha-beta T cell proliferation | 0.000160004 | 3 |
| BP | GO:0061082 | myeloid leukocyte cytokine production | 0.000160004 | 3 |
| BP | GO:0071480 | cellular response to gamma radiation | 0.000160004 | 3 |
| BP | GO:0071549 | cellular response to dexamethasone stimulus | 0.000160004 | 3 |
| BP | GO:0071901 | negative regulation of protein serine/threonine kinase activity | 0.000161013 | 5 |
| BP | GO:0042742 | defense response to bacterium | 0.000161095 | 7 |
| BP | GO:0046942 | carboxylic acid transport | 0.00016412 | 7 |
| BP | GO:0055123 | digestive system development | 0.00016627 | 5 |
| BP | GO:0008306 | associative learning | 0.000166914 | 4 |
| BP | GO:0055021 | regulation of cardiac muscle tissue growth | 0.000166914 | 4 |
| BP | GO:0072347 | response to anesthetic | 0.000166914 | 4 |
| BP | GO:0090150 | establishment of protein localization to membrane | 0.000167192 | 7 |
| BP | GO:0071695 | anatomical structure maturation | 0.000168294 | 6 |
| BP | GO:0015849 | organic acid transport | 0.000170309 | 7 |
| BP | GO:0042445 | hormone metabolic process | 0.000172267 | 6 |
| BP | GO:0048738 | cardiac muscle tissue development | 0.000172267 | 6 |
| BP | GO:0097305 | response to alcohol | 0.000172267 | 6 |
| BP | GO:0007265 | Ras protein signal transduction | 0.000175292 | 8 |
| BP | GO:0031110 | regulation of microtubule polymerization or depolymerization | 0.000175351 | 4 |
| BP | GO:0048588 | developmental cell growth | 0.000176315 | 6 |
| BP | GO:0002675 | positive regulation of acute inflammatory response | 0.000176701 | 3 |
| BP | GO:0032770 | positive regulation of monooxygenase activity | 0.000176701 | 3 |
| BP | GO:0034694 | response to prostaglandin | 0.000176701 | 3 |
| BP | GO:1902895 | positive regulation of pri-miRNA transcription by RNA polymerase II | 0.000176701 | 3 |
| BP | GO:0008643 | carbohydrate transport | 0.000177179 | 5 |
| BP | GO:0051186 | cofactor metabolic process | 0.000177977 | 8 |
| BP | GO:0051056 | regulation of small GTPase mediated signal transduction | 0.000186614 | 7 |
| BP | GO:0006090 | pyruvate metabolic process | 0.000188627 | 5 |
| BP | GO:0016331 | morphogenesis of embryonic epithelium | 0.000188627 | 5 |
| BP | GO:0048754 | branching morphogenesis of an epithelial tube | 0.000188627 | 5 |
| BP | GO:0007050 | cell cycle arrest | 0.000188913 | 6 |
| BP | GO:0014855 | striated muscle cell proliferation | 0.000193143 | 4 |
| BP | GO:0043552 | positive regulation of phosphatidylinositol 3-kinase activity | 0.000194488 | 3 |
| BP | GO:0051968 | positive regulation of synaptic transmission, glutamatergic | 0.000194488 | 3 |
| BP | GO:0060325 | face morphogenesis | 0.000194488 | 3 |
| BP | GO:0060674 | placenta blood vessel development | 0.000194488 | 3 |
| BP | GO:0031348 | negative regulation of defense response | 0.000197699 | 6 |
| BP | GO:1904062 | regulation of cation transmembrane transport | 0.000200549 | 7 |
| BP | GO:1903169 | regulation of calcium ion transmembrane transport | 0.000200631 | 5 |
| BP | GO:0034644 | cellular response to UV | 0.000202511 | 4 |
| BP | GO:0051279 | regulation of release of sequestered calcium ion into cytosol | 0.000202511 | 4 |
| BP | GO:0006970 | response to osmotic stress | 0.000212203 | 4 |
| BP | GO:0045582 | positive regulation of T cell differentiation | 0.000212203 | 4 |
| BP | GO:0009132 | nucleoside diphosphate metabolic process | 0.000213209 | 5 |
| BP | GO:0010039 | response to iron ion | 0.000213397 | 3 |
| BP | GO:0016242 | negative regulation of macroautophagy | 0.000213397 | 3 |
| BP | GO:0033028 | myeloid cell apoptotic process | 0.000213397 | 3 |
| BP | GO:0046633 | alpha-beta T cell proliferation | 0.000213397 | 3 |
| BP | GO:0002718 | regulation of cytokine production involved in immune response | 0.000222224 | 4 |
| BP | GO:0051492 | regulation of stress fiber assembly | 0.000222224 | 4 |
| BP | GO:0060420 | regulation of heart growth | 0.000222224 | 4 |
| BP | GO:0097756 | negative regulation of blood vessel diameter | 0.000222224 | 4 |
| BP | GO:0072521 | purine-containing compound metabolic process | 0.0002225 | 8 |
| BP | GO:0048864 | stem cell development | 0.000232582 | 4 |
| BP | GO:0034205 | amyloid-beta formation | 0.000233459 | 3 |
| BP | GO:0060402 | calcium ion transport into cytosol | 0.000240162 | 5 |
| BP | GO:0030101 | natural killer cell activation | 0.000243283 | 4 |
| BP | GO:0042058 | regulation of epidermal growth factor receptor signaling pathway | 0.000243283 | 4 |
| BP | GO:0070542 | response to fatty acid | 0.000243283 | 4 |
| BP | GO:0090559 | regulation of membrane permeability | 0.000243283 | 4 |
| BP | GO:0002833 | positive regulation of response to biotic stimulus | 0.000246595 | 6 |
| BP | GO:0006352 | DNA-templated transcription, initiation | 0.000246595 | 6 |
| BP | GO:0031589 | cell-substrate adhesion | 0.000247484 | 7 |
| BP | GO:0003012 | muscle system process | 0.00024976 | 8 |
| BP | GO:0043470 | regulation of carbohydrate catabolic process | 0.000254334 | 4 |
| BP | GO:0032735 | positive regulation of interleukin-12 production | 0.000254702 | 3 |
| BP | GO:0071312 | cellular response to alkaloid | 0.000254702 | 3 |
| BP | GO:1902991 | regulation of amyloid precursor protein catabolic process | 0.000254702 | 3 |
| BP | GO:1905332 | positive regulation of morphogenesis of an epithelium | 0.000254702 | 3 |
| BP | GO:2001024 | negative regulation of response to drug | 0.000254702 | 3 |
| BP | GO:0008016 | regulation of heart contraction | 0.000257428 | 6 |
| BP | GO:0050829 | defense response to Gram-negative bacterium | 0.000265741 | 4 |
| BP | GO:0051973 | positive regulation of telomerase activity | 0.000277156 | 3 |
| BP | GO:0015718 | monocarboxylic acid transport | 0.000277414 | 5 |
| BP | GO:0055013 | cardiac muscle cell development | 0.000277511 | 4 |
| BP | GO:0032651 | regulation of interleukin-1 beta production | 0.000289652 | 4 |
| BP | GO:0071674 | mononuclear cell migration | 0.000289652 | 4 |
| BP | GO:0022604 | regulation of cell morphogenesis | 0.000295748 | 8 |
| BP | GO:0002714 | positive regulation of B cell mediated immunity | 0.000300851 | 3 |
| BP | GO:0002891 | positive regulation of immunoglobulin mediated immune response | 0.000300851 | 3 |
| BP | GO:0043029 | T cell homeostasis | 0.000300851 | 3 |
| BP | GO:0045923 | positive regulation of fatty acid metabolic process | 0.000300851 | 3 |
| BP | GO:0060323 | head morphogenesis | 0.000300851 | 3 |
| BP | GO:0090050 | positive regulation of cell migration involved in sprouting angiogenesis | 0.000300851 | 3 |
| BP | GO:2000249 | regulation of actin cytoskeleton reorganization | 0.000300851 | 3 |
| BP | GO:0002244 | hematopoietic progenitor cell differentiation | 0.000301779 | 5 |
| BP | GO:1901992 | positive regulation of mitotic cell cycle phase transition | 0.000302169 | 4 |
| BP | GO:0033209 | tumor necrosis factor-mediated signaling pathway | 0.00031025 | 5 |
| BP | GO:1901568 | fatty acid derivative metabolic process | 0.00031025 | 5 |
| BP | GO:0045833 | negative regulation of lipid metabolic process | 0.000315069 | 4 |
| BP | GO:0110053 | regulation of actin filament organization | 0.000317343 | 6 |
| BP | GO:0015850 | organic hydroxy compound transport | 0.00032389 | 6 |
| BP | GO:0046883 | regulation of hormone secretion | 0.00032389 | 6 |
| BP | GO:0010907 | positive regulation of glucose metabolic process | 0.000325815 | 3 |
| BP | GO:0016572 | histone phosphorylation | 0.000325815 | 3 |
| BP | GO:0032094 | response to food | 0.000325815 | 3 |
| BP | GO:0045022 | early endosome to late endosome transport | 0.000325815 | 3 |
| BP | GO:0046825 | regulation of protein export from nucleus | 0.000325815 | 3 |
| BP | GO:1903523 | negative regulation of blood circulation | 0.000325815 | 3 |
| BP | GO:0033273 | response to vitamin | 0.00032836 | 4 |
| BP | GO:0035249 | synaptic transmission, glutamatergic | 0.00032836 | 4 |
| BP | GO:0046634 | regulation of alpha-beta T cell activation | 0.00032836 | 4 |
| BP | GO:0045666 | positive regulation of neuron differentiation | 0.000328775 | 7 |
| BP | GO:0045621 | positive regulation of lymphocyte differentiation | 0.000342049 | 4 |
| BP | GO:0015711 | organic anion transport | 0.000343793 | 8 |
| BP | GO:0032874 | positive regulation of stress-activated MAPK cascade | 0.00035535 | 5 |
| BP | GO:0042102 | positive regulation of T cell proliferation | 0.000356141 | 4 |
| BP | GO:0055006 | cardiac cell development | 0.000356141 | 4 |
| BP | GO:0110020 | regulation of actomyosin structure organization | 0.000356141 | 4 |
| BP | GO:0002446 | neutrophil mediated immunity | 0.000362772 | 8 |
| BP | GO:0070304 | positive regulation of stress-activated protein kinase signaling cascade | 0.000364935 | 5 |
| BP | GO:0051281 | positive regulation of release of sequestered calcium ion into cytosol | 0.000379664 | 3 |
| BP | GO:0071548 | response to dexamethasone | 0.000379664 | 3 |
| BP | GO:0072210 | metanephric nephron development | 0.000379664 | 3 |
| BP | GO:0003018 | vascular process in circulatory system | 0.000384692 | 5 |
| BP | GO:0010921 | regulation of phosphatase activity | 0.000384692 | 5 |
| BP | GO:0120032 | regulation of plasma membrane bounded cell projection assembly | 0.000384692 | 5 |
| BP | GO:0014020 | primary neural tube formation | 0.000385565 | 4 |
| BP | GO:0032231 | regulation of actin filament bundle assembly | 0.000385565 | 4 |
| BP | GO:0048709 | oligodendrocyte differentiation | 0.000385565 | 4 |
| BP | GO:0051591 | response to cAMP | 0.000385565 | 4 |
| BP | GO:0050796 | regulation of insulin secretion | 0.000394869 | 5 |
| BP | GO:0060401 | cytosolic calcium ion transport | 0.000394869 | 5 |
| BP | GO:0002042 | cell migration involved in sprouting angiogenesis | 0.000400911 | 4 |
| BP | GO:0030038 | contractile actin filament bundle assembly | 0.000400911 | 4 |
| BP | GO:0043149 | stress fiber assembly | 0.000400911 | 4 |
| BP | GO:0045807 | positive regulation of endocytosis | 0.000400911 | 4 |
| BP | GO:0050764 | regulation of phagocytosis | 0.000400911 | 4 |
| BP | GO:0061448 | connective tissue development | 0.000403139 | 6 |
| BP | GO:0048469 | cell maturation | 0.000405248 | 5 |
| BP | GO:0060491 | regulation of cell projection assembly | 0.000405248 | 5 |
| BP | GO:0030890 | positive regulation of B cell proliferation | 0.000408606 | 3 |
| BP | GO:0050798 | activated T cell proliferation | 0.000408606 | 3 |
| BP | GO:0098927 | vesicle-mediated transport between endosomal compartments | 0.000408606 | 3 |
| BP | GO:0062014 | negative regulation of small molecule metabolic process | 0.000416689 | 4 |
| BP | GO:1901570 | fatty acid derivative biosynthetic process | 0.000416689 | 4 |
| BP | GO:0043270 | positive regulation of ion transport | 0.000419049 | 6 |
| BP | GO:0055024 | regulation of cardiac muscle tissue development | 0.000432905 | 4 |
| BP | GO:0046688 | response to copper ion | 0.000438929 | 3 |
| BP | GO:0009165 | nucleotide biosynthetic process | 0.000443831 | 6 |
| BP | GO:0007631 | feeding behavior | 0.000449567 | 4 |
| BP | GO:0031532 | actin cytoskeleton reorganization | 0.000449567 | 4 |
| BP | GO:0032611 | interleukin-1 beta production | 0.000449567 | 4 |
| BP | GO:1901890 | positive regulation of cell junction assembly | 0.000449567 | 4 |
| BP | GO:0061138 | morphogenesis of a branching epithelium | 0.000460271 | 5 |
| BP | GO:0010522 | regulation of calcium ion transport into cytosol | 0.000466682 | 4 |
| BP | GO:0021549 | cerebellum development | 0.000466682 | 4 |
| BP | GO:0006692 | prostanoid metabolic process | 0.00047066 | 3 |
| BP | GO:0006693 | prostaglandin metabolic process | 0.00047066 | 3 |
| BP | GO:0042088 | T-helper 1 type immune response | 0.00047066 | 3 |
| BP | GO:0050850 | positive regulation of calcium-mediated signaling | 0.00047066 | 3 |
| BP | GO:0002040 | sprouting angiogenesis | 0.00047192 | 5 |
| BP | GO:0038061 | NIK/NF-kappaB signaling | 0.00047192 | 5 |
| BP | GO:0043123 | positive regulation of I-kappaB kinase/NF-kappaB signaling | 0.00047192 | 5 |
| BP | GO:0044262 | cellular carbohydrate metabolic process | 0.000478639 | 6 |
| BP | GO:1901293 | nucleoside phosphate biosynthetic process | 0.000478639 | 6 |
| BP | GO:0048639 | positive regulation of developmental growth | 0.00048379 | 5 |
| BP | GO:0050864 | regulation of B cell activation | 0.00048379 | 5 |
| BP | GO:0032652 | regulation of interleukin-1 production | 0.000484257 | 4 |
| BP | GO:1902106 | negative regulation of leukocyte differentiation | 0.000484257 | 4 |
| BP | GO:0032409 | regulation of transporter activity | 0.000487665 | 6 |
| BP | GO:0021700 | developmental maturation | 0.000496824 | 6 |
| BP | GO:0008630 | intrinsic apoptotic signaling pathway in response to DNA damage | 0.000502298 | 4 |
| BP | GO:0032965 | regulation of collagen biosynthetic process | 0.000503826 | 3 |
| BP | GO:0034105 | positive regulation of tissue remodeling | 0.000503826 | 3 |
| BP | GO:0038084 | vascular endothelial growth factor signaling pathway | 0.000503826 | 3 |
| BP | GO:0042987 | amyloid precursor protein catabolic process | 0.000503826 | 3 |
| BP | GO:0070266 | necroptotic process | 0.000503826 | 3 |
| BP | GO:0015980 | energy derivation by oxidation of organic compounds | 0.000515544 | 6 |
| BP | GO:0007015 | actin filament organization | 0.000515996 | 7 |
| BP | GO:0001841 | neural tube formation | 0.000520813 | 4 |
| BP | GO:0002821 | positive regulation of adaptive immune response | 0.000520813 | 4 |
| BP | GO:0006641 | triglyceride metabolic process | 0.000520813 | 4 |
| BP | GO:0007009 | plasma membrane organization | 0.000520813 | 4 |
| BP | GO:0055017 | cardiac muscle tissue growth | 0.000520813 | 4 |
| BP | GO:0051656 | establishment of organelle localization | 0.000523712 | 7 |
| BP | GO:0006367 | transcription initiation from RNA polymerase II promoter | 0.000533533 | 5 |
| BP | GO:0051204 | protein insertion into mitochondrial membrane | 0.000538455 | 3 |
| BP | GO:0002286 | T cell activation involved in immune response | 0.000539809 | 4 |
| BP | GO:0051963 | regulation of synapse assembly | 0.000539809 | 4 |
| BP | GO:1901989 | positive regulation of cell cycle phase transition | 0.000539809 | 4 |
| BP | GO:0001660 | fever generation | 0.000543828 | 2 |
| BP | GO:0031284 | positive regulation of guanylate cyclase activity | 0.000543828 | 2 |
| BP | GO:0032025 | response to cobalt ion | 0.000543828 | 2 |
| BP | GO:0033210 | leptin-mediated signaling pathway | 0.000543828 | 2 |
| BP | GO:0045792 | negative regulation of cell size | 0.000543828 | 2 |
| BP | GO:0045945 | positive regulation of transcription by RNA polymerase III | 0.000543828 | 2 |
| BP | GO:0048304 | positive regulation of isotype switching to IgG isotypes | 0.000543828 | 2 |
| BP | GO:0070391 | response to lipoteichoic acid | 0.000543828 | 2 |
| BP | GO:0071223 | cellular response to lipoteichoic acid | 0.000543828 | 2 |
| BP | GO:0072203 | cell proliferation involved in metanephros development | 0.000543828 | 2 |
| BP | GO:0090154 | positive regulation of sphingolipid biosynthetic process | 0.000543828 | 2 |
| BP | GO:0090336 | positive regulation of brown fat cell differentiation | 0.000543828 | 2 |
| BP | GO:0140052 | cellular response to oxidised low-density lipoprotein particle stimulus | 0.000543828 | 2 |
| BP | GO:1903800 | positive regulation of production of miRNAs involved in gene silencing by miRNA | 0.000543828 | 2 |
| BP | GO:1990416 | cellular response to brain-derived neurotrophic factor stimulus | 0.000543828 | 2 |
| BP | GO:2000304 | positive regulation of ceramide biosynthetic process | 0.000543828 | 2 |
| BP | GO:0001909 | leukocyte mediated cytotoxicity | 0.000559293 | 4 |
| BP | GO:0031648 | protein destabilization | 0.000574571 | 3 |
| BP | GO:0035196 | production of miRNAs involved in gene silencing by miRNA | 0.000574571 | 3 |
| BP | GO:0045776 | negative regulation of blood pressure | 0.000574571 | 3 |
| BP | GO:0001676 | long-chain fatty acid metabolic process | 0.000599754 | 4 |
| BP | GO:0021695 | cerebellar cortex development | 0.000612202 | 3 |
| BP | GO:0050435 | amyloid-beta metabolic process | 0.000612202 | 3 |
| BP | GO:0090151 | establishment of protein localization to mitochondrial membrane | 0.000612202 | 3 |
| BP | GO:1903580 | positive regulation of ATP metabolic process | 0.000612202 | 3 |
| BP | GO:0033559 | unsaturated fatty acid metabolic process | 0.000620745 | 4 |
| BP | GO:0090068 | positive regulation of cell cycle process | 0.000639712 | 6 |
| BP | GO:0022037 | metencephalon development | 0.000642252 | 4 |
| BP | GO:0001763 | morphogenesis of a branching structure | 0.000644469 | 5 |
| BP | GO:0055023 | positive regulation of cardiac muscle tissue growth | 0.000651372 | 3 |
| BP | GO:0002923 | regulation of humoral immune response mediated by circulating immunoglobulin | 0.000663162 | 2 |
| BP | GO:0019371 | cyclooxygenase pathway | 0.000663162 | 2 |
| BP | GO:0031652 | positive regulation of heat generation | 0.000663162 | 2 |
| BP | GO:0033327 | Leydig cell differentiation | 0.000663162 | 2 |
| BP | GO:0033629 | negative regulation of cell adhesion mediated by integrin | 0.000663162 | 2 |
| BP | GO:0035747 | natural killer cell chemotaxis | 0.000663162 | 2 |
| BP | GO:0045060 | negative thymic T cell selection | 0.000663162 | 2 |
| BP | GO:0046886 | positive regulation of hormone biosynthetic process | 0.000663162 | 2 |
| BP | GO:0060736 | prostate gland growth | 0.000663162 | 2 |
| BP | GO:0061307 | cardiac neural crest cell differentiation involved in heart development | 0.000663162 | 2 |
| BP | GO:0061308 | cardiac neural crest cell development involved in heart development | 0.000663162 | 2 |
| BP | GO:0072584 | caveolin-mediated endocytosis | 0.000663162 | 2 |
| BP | GO:2001269 | positive regulation of cysteine-type endopeptidase activity involved in apoptotic signaling pathway | 0.000663162 | 2 |
| BP | GO:0010906 | regulation of glucose metabolic process | 0.000664283 | 4 |
| BP | GO:0031109 | microtubule polymerization or depolymerization | 0.000664283 | 4 |
| BP | GO:0060419 | heart growth | 0.000664283 | 4 |
| BP | GO:0016052 | carbohydrate catabolic process | 0.00067472 | 5 |
| BP | GO:0010712 | regulation of collagen metabolic process | 0.000692106 | 3 |
| BP | GO:0048546 | digestive tract morphogenesis | 0.000692106 | 3 |
| BP | GO:0097300 | programmed necrotic cell death | 0.000692106 | 3 |
| BP | GO:0046034 | ATP metabolic process | 0.000709923 | 6 |
| BP | GO:0042752 | regulation of circadian rhythm | 0.000709943 | 4 |
| BP | GO:0045446 | endothelial cell differentiation | 0.000709943 | 4 |
| BP | GO:0032612 | interleukin-1 production | 0.000733588 | 4 |
| BP | GO:0009409 | response to cold | 0.000734431 | 3 |
| BP | GO:0010171 | body morphogenesis | 0.000734431 | 3 |
| BP | GO:1900087 | positive regulation of G1/S transition of mitotic cell cycle | 0.000734431 | 3 |
| BP | GO:0051701 | interaction with host | 0.000738402 | 5 |
| BP | GO:0031396 | regulation of protein ubiquitination | 0.00077188 | 5 |
| BP | GO:0002712 | regulation of B cell mediated immunity | 0.00077837 | 3 |
| BP | GO:0002889 | regulation of immunoglobulin mediated immune response | 0.00077837 | 3 |
| BP | GO:0007566 | embryo implantation | 0.00077837 | 3 |
| BP | GO:0019369 | arachidonic acid metabolic process | 0.00077837 | 3 |
| BP | GO:0031050 | dsRNA processing | 0.00077837 | 3 |
| BP | GO:0070918 | production of small RNA involved in gene silencing by RNA | 0.00077837 | 3 |
| BP | GO:1901222 | regulation of NIK/NF-kappaB signaling | 0.00078254 | 4 |
| BP | GO:0002371 | dendritic cell cytokine production | 0.00079398 | 2 |
| BP | GO:0002551 | mast cell chemotaxis | 0.00079398 | 2 |
| BP | GO:0006527 | arginine catabolic process | 0.00079398 | 2 |
| BP | GO:0010623 | programmed cell death involved in cell development | 0.00079398 | 2 |
| BP | GO:0010889 | regulation of sequestering of triglyceride | 0.00079398 | 2 |
| BP | GO:0031282 | regulation of guanylate cyclase activity | 0.00079398 | 2 |
| BP | GO:0031392 | regulation of prostaglandin biosynthetic process | 0.00079398 | 2 |
| BP | GO:0042368 | vitamin D biosynthetic process | 0.00079398 | 2 |
| BP | GO:0043383 | negative T cell selection | 0.00079398 | 2 |
| BP | GO:0045080 | positive regulation of chemokine biosynthetic process | 0.00079398 | 2 |
| BP | GO:0045722 | positive regulation of gluconeogenesis | 0.00079398 | 2 |
| BP | GO:0048302 | regulation of isotype switching to IgG isotypes | 0.00079398 | 2 |
| BP | GO:0051549 | positive regulation of keratinocyte migration | 0.00079398 | 2 |
| BP | GO:0051873 | killing by host of symbiont cells | 0.00079398 | 2 |
| BP | GO:0070493 | thrombin-activated receptor signaling pathway | 0.00079398 | 2 |
| BP | GO:0097531 | mast cell migration | 0.00079398 | 2 |
| BP | GO:0003179 | heart valve morphogenesis | 0.000823947 | 3 |
| BP | GO:0009620 | response to fungus | 0.000823947 | 3 |
| BP | GO:0031529 | ruffle organization | 0.000823947 | 3 |
| BP | GO:0060421 | positive regulation of heart growth | 0.000823947 | 3 |
| BP | GO:0070555 | response to interleukin-1 | 0.000824209 | 5 |
| BP | GO:0042391 | regulation of membrane potential | 0.00083484 | 7 |
| BP | GO:0006898 | receptor-mediated endocytosis | 0.000840073 | 6 |
| BP | GO:0022612 | gland morphogenesis | 0.000860236 | 4 |
| BP | GO:0051961 | negative regulation of nervous system development | 0.000868181 | 6 |
| BP | GO:0032964 | collagen biosynthetic process | 0.000871187 | 3 |
| BP | GO:0035065 | regulation of histone acetylation | 0.000871187 | 3 |
| BP | GO:0071320 | cellular response to cAMP | 0.000871187 | 3 |
| BP | GO:0006650 | glycerophospholipid metabolic process | 0.000911701 | 6 |
| BP | GO:0070588 | calcium ion transmembrane transport | 0.000911701 | 6 |
| BP | GO:1990778 | protein localization to cell periphery | 0.000911701 | 6 |
| BP | GO:0002720 | positive regulation of cytokine production involved in immune response | 0.000920114 | 3 |
| BP | GO:0006636 | unsaturated fatty acid biosynthetic process | 0.000920114 | 3 |
| BP | GO:0010524 | positive regulation of calcium ion transport into cytosol | 0.000920114 | 3 |
| BP | GO:0032655 | regulation of interleukin-12 production | 0.000920114 | 3 |
| BP | GO:0045747 | positive regulation of Notch signaling pathway | 0.000920114 | 3 |
| BP | GO:0002679 | respiratory burst involved in defense response | 0.000936201 | 2 |
| BP | GO:0010745 | negative regulation of macrophage derived foam cell differentiation | 0.000936201 | 2 |
| BP | GO:0010870 | positive regulation of receptor biosynthetic process | 0.000936201 | 2 |
| BP | GO:0014745 | negative regulation of muscle adaptation | 0.000936201 | 2 |
| BP | GO:0014842 | regulation of skeletal muscle satellite cell proliferation | 0.000936201 | 2 |
| BP | GO:0019896 | axonal transport of mitochondrion | 0.000936201 | 2 |
| BP | GO:0031650 | regulation of heat generation | 0.000936201 | 2 |
| BP | GO:0035723 | interleukin-15-mediated signaling pathway | 0.000936201 | 2 |
| BP | GO:0045059 | positive thymic T cell selection | 0.000936201 | 2 |
| BP | GO:0045820 | negative regulation of glycolytic process | 0.000936201 | 2 |
| BP | GO:0048291 | isotype switching to IgG isotypes | 0.000936201 | 2 |
| BP | GO:0050722 | regulation of interleukin-1 beta biosynthetic process | 0.000936201 | 2 |
| BP | GO:0050872 | white fat cell differentiation | 0.000936201 | 2 |
| BP | GO:0071287 | cellular response to manganese ion | 0.000936201 | 2 |
| BP | GO:0071350 | cellular response to interleukin-15 | 0.000936201 | 2 |
| BP | GO:0071872 | cellular response to epinephrine stimulus | 0.000936201 | 2 |
| BP | GO:0072672 | neutrophil extravasation | 0.000936201 | 2 |
| BP | GO:1900272 | negative regulation of long-term synaptic potentiation | 0.000936201 | 2 |
| BP | GO:1903960 | negative regulation of anion transmembrane transport | 0.000936201 | 2 |
| BP | GO:1904251 | regulation of bile acid metabolic process | 0.000936201 | 2 |
| BP | GO:0051928 | positive regulation of calcium ion transport | 0.000943226 | 4 |
| BP | GO:0045089 | positive regulation of innate immune response | 0.000956588 | 5 |
| BP | GO:0010256 | endomembrane system organization | 0.000966062 | 7 |
| BP | GO:0001541 | ovarian follicle development | 0.00097075 | 3 |
| BP | GO:0032613 | interleukin-10 production | 0.00097075 | 3 |
| BP | GO:0043030 | regulation of macrophage activation | 0.00097075 | 3 |
| BP | GO:0001838 | embryonic epithelial tube formation | 0.000972099 | 4 |
| BP | GO:0008637 | apoptotic mitochondrial changes | 0.000972099 | 4 |
| BP | GO:0014013 | regulation of gliogenesis | 0.000972099 | 4 |
| BP | GO:0019218 | regulation of steroid metabolic process | 0.000972099 | 4 |
| BP | GO:0046717 | acid secretion | 0.000972099 | 4 |
| BP | GO:0051101 | regulation of DNA binding | 0.000972099 | 4 |
| BP | GO:0045471 | response to ethanol | 0.001001588 | 4 |
| BP | GO:0016485 | protein processing | 0.001017923 | 5 |
| BP | GO:0046474 | glycerophospholipid biosynthetic process | 0.001017923 | 5 |
| BP | GO:0090183 | regulation of kidney development | 0.00102312 | 3 |
| BP | GO:0032479 | regulation of type I interferon production | 0.001031703 | 4 |
| BP | GO:0032886 | regulation of microtubule-based process | 0.001039004 | 5 |
| BP | GO:0046887 | positive regulation of hormone secretion | 0.001062448 | 4 |
| BP | GO:0042743 | hydrogen peroxide metabolic process | 0.001077245 | 3 |
| BP | GO:0071398 | cellular response to fatty acid | 0.001077245 | 3 |
| BP | GO:0048762 | mesenchymal cell differentiation | 0.001082137 | 5 |
| BP | GO:0001655 | urogenital system development | 0.001085856 | 6 |
| BP | GO:0014841 | skeletal muscle satellite cell proliferation | 0.001089744 | 2 |
| BP | GO:0014857 | regulation of skeletal muscle cell proliferation | 0.001089744 | 2 |
| BP | GO:0035791 | platelet-derived growth factor receptor-beta signaling pathway | 0.001089744 | 2 |
| BP | GO:0046629 | gamma-delta T cell activation | 0.001089744 | 2 |
| BP | GO:0050720 | interleukin-1 beta biosynthetic process | 0.001089744 | 2 |
| BP | GO:0051547 | regulation of keratinocyte migration | 0.001089744 | 2 |
| BP | GO:0060009 | Sertoli cell development | 0.001089744 | 2 |
| BP | GO:0060397 | growth hormone receptor signaling pathway via JAK-STAT | 0.001089744 | 2 |
| BP | GO:0070672 | response to interleukin-15 | 0.001089744 | 2 |
| BP | GO:0071391 | cellular response to estrogen stimulus | 0.001089744 | 2 |
| BP | GO:2001279 | regulation of unsaturated fatty acid biosynthetic process | 0.001089744 | 2 |
| BP | GO:0002576 | platelet degranulation | 0.001093832 | 4 |
| BP | GO:0032606 | type I interferon production | 0.001093832 | 4 |
| BP | GO:0034763 | negative regulation of transmembrane transport | 0.001093832 | 4 |
| BP | GO:0006639 | acylglycerol metabolic process | 0.001125862 | 4 |
| BP | GO:0071482 | cellular response to light stimulus | 0.001125862 | 4 |
| BP | GO:0042093 | T-helper cell differentiation | 0.001133148 | 3 |
| BP | GO:0071385 | cellular response to glucocorticoid stimulus | 0.001133148 | 3 |
| BP | GO:0001885 | endothelial cell development | 0.001190852 | 3 |
| BP | GO:0032890 | regulation of organic acid transport | 0.001190852 | 3 |
| BP | GO:0043388 | positive regulation of DNA binding | 0.001190852 | 3 |
| BP | GO:2000756 | regulation of peptidyl-lysine acetylation | 0.001190852 | 3 |
| BP | GO:0007160 | cell-matrix adhesion | 0.001195787 | 5 |
| BP | GO:0003158 | endothelium development | 0.001225897 | 4 |
| BP | GO:0042552 | myelination | 0.001225897 | 4 |
| BP | GO:0055007 | cardiac muscle cell differentiation | 0.001225897 | 4 |
| BP | GO:0072175 | epithelial tube formation | 0.001225897 | 4 |
| BP | GO:0002294 | CD4-positive, alpha-beta T cell differentiation involved in immune response | 0.001250379 | 3 |
| BP | GO:1902808 | positive regulation of cell cycle G1/S phase transition | 0.001250379 | 3 |
| BP | GO:0010759 | positive regulation of macrophage chemotaxis | 0.001254532 | 2 |
| BP | GO:0014856 | skeletal muscle cell proliferation | 0.001254532 | 2 |
| BP | GO:0017014 | protein nitrosylation | 0.001254532 | 2 |
| BP | GO:0018119 | peptidyl-cysteine S-nitrosylation | 0.001254532 | 2 |
| BP | GO:0032352 | positive regulation of hormone metabolic process | 0.001254532 | 2 |
| BP | GO:0034349 | glial cell apoptotic process | 0.001254532 | 2 |
| BP | GO:0035067 | negative regulation of histone acetylation | 0.001254532 | 2 |
| BP | GO:0042362 | fat-soluble vitamin biosynthetic process | 0.001254532 | 2 |
| BP | GO:0043518 | negative regulation of DNA damage response, signal transduction by p53 class mediator | 0.001254532 | 2 |
| BP | GO:0045073 | regulation of chemokine biosynthetic process | 0.001254532 | 2 |
| BP | GO:0045360 | regulation of interleukin-1 biosynthetic process | 0.001254532 | 2 |
| BP | GO:0046321 | positive regulation of fatty acid oxidation | 0.001254532 | 2 |
| BP | GO:0051883 | killing of cells in other organism involved in symbiotic interaction | 0.001254532 | 2 |
| BP | GO:0072075 | metanephric mesenchyme development | 0.001254532 | 2 |
| BP | GO:1903729 | regulation of plasma membrane organization | 0.001254532 | 2 |
| BP | GO:2001028 | positive regulation of endothelial cell chemotaxis | 0.001254532 | 2 |
| BP | GO:0042770 | signal transduction in response to DNA damage | 0.001260582 | 4 |
| BP | GO:0007272 | ensheathment of neurons | 0.001295948 | 4 |
| BP | GO:0008366 | axon ensheathment | 0.001295948 | 4 |
| BP | GO:0033044 | regulation of chromosome organization | 0.001303916 | 6 |
| BP | GO:0002287 | alpha-beta T cell activation involved in immune response | 0.00131175 | 3 |
| BP | GO:0002293 | alpha-beta T cell differentiation involved in immune response | 0.00131175 | 3 |
| BP | GO:0003170 | heart valve development | 0.00131175 | 3 |
| BP | GO:0010803 | regulation of tumor necrosis factor-mediated signaling pathway | 0.00131175 | 3 |
| BP | GO:0071384 | cellular response to corticosteroid stimulus | 0.00131175 | 3 |
| BP | GO:0010721 | negative regulation of cell development | 0.001343301 | 6 |
| BP | GO:1903320 | regulation of protein modification by small protein conjugation or removal | 0.001343583 | 5 |
| BP | GO:0046635 | positive regulation of alpha-beta T cell activation | 0.001374986 | 3 |
| BP | GO:0055025 | positive regulation of cardiac muscle tissue development | 0.001374986 | 3 |
| BP | GO:0070265 | necrotic cell death | 0.001374986 | 3 |
| BP | GO:1903670 | regulation of sprouting angiogenesis | 0.00140621 | 4 |
| BP | GO:0001780 | neutrophil homeostasis | 0.001430485 | 2 |
| BP | GO:0002295 | T-helper cell lineage commitment | 0.001430485 | 2 |
| BP | GO:0006206 | pyrimidine nucleobase metabolic process | 0.001430485 | 2 |
| BP | GO:0006837 | serotonin transport | 0.001430485 | 2 |
| BP | GO:0010934 | macrophage cytokine production | 0.001430485 | 2 |
| BP | GO:0031958 | corticosteroid receptor signaling pathway | 0.001430485 | 2 |
| BP | GO:0032225 | regulation of synaptic transmission, dopaminergic | 0.001430485 | 2 |
| BP | GO:0042033 | chemokine biosynthetic process | 0.001430485 | 2 |
| BP | GO:0042222 | interleukin-1 biosynthetic process | 0.001430485 | 2 |
| BP | GO:0050755 | chemokine metabolic process | 0.001430485 | 2 |
| BP | GO:0060965 | negative regulation of gene silencing by miRNA | 0.001430485 | 2 |
| BP | GO:0061684 | chaperone-mediated autophagy | 0.001430485 | 2 |
| BP | GO:0071380 | cellular response to prostaglandin E stimulus | 0.001430485 | 2 |
| BP | GO:0071871 | response to epinephrine | 0.001430485 | 2 |
| BP | GO:0072224 | metanephric glomerulus development | 0.001430485 | 2 |
| BP | GO:0090153 | regulation of sphingolipid biosynthetic process | 0.001430485 | 2 |
| BP | GO:1905038 | regulation of membrane lipid metabolic process | 0.001430485 | 2 |
| BP | GO:2000303 | regulation of ceramide biosynthetic process | 0.001430485 | 2 |
| BP | GO:0002637 | regulation of immunoglobulin production | 0.001440109 | 3 |
| BP | GO:0042982 | amyloid precursor protein metabolic process | 0.001440109 | 3 |
| BP | GO:0046637 | regulation of alpha-beta T cell differentiation | 0.001440109 | 3 |
| BP | GO:0060560 | developmental growth involved in morphogenesis | 0.001449347 | 5 |
| BP | GO:0016311 | dephosphorylation | 0.001459732 | 7 |
| BP | GO:0006360 | transcription by RNA polymerase I | 0.001507139 | 3 |
| BP | GO:0035023 | regulation of Rho protein signal transduction | 0.001522865 | 4 |
| BP | GO:0060048 | cardiac muscle contraction | 0.001522865 | 4 |
| BP | GO:0043312 | neutrophil degranulation | 0.001586143 | 7 |
| BP | GO:0050871 | positive regulation of B cell activation | 0.001604283 | 4 |
| BP | GO:0031649 | heat generation | 0.001617523 | 2 |
| BP | GO:0035855 | megakaryocyte development | 0.001617523 | 2 |
| BP | GO:0042953 | lipoprotein transport | 0.001617523 | 2 |
| BP | GO:0043373 | CD4-positive, alpha-beta T cell lineage commitment | 0.001617523 | 2 |
| BP | GO:0044872 | lipoprotein localization | 0.001617523 | 2 |
| BP | GO:0060644 | mammary gland epithelial cell differentiation | 0.001617523 | 2 |
| BP | GO:0090185 | negative regulation of kidney development | 0.001617523 | 2 |
| BP | GO:1904355 | positive regulation of telomere capping | 0.001617523 | 2 |
| BP | GO:1904996 | positive regulation of leukocyte adhesion to vascular endothelial cell | 0.001617523 | 2 |
| BP | GO:1905331 | negative regulation of morphogenesis of an epithelium | 0.001617523 | 2 |
| BP | GO:2000811 | negative regulation of anoikis | 0.001617523 | 2 |
| BP | GO:0002283 | neutrophil activation involved in immune response | 0.001642871 | 7 |
| BP | GO:0031056 | regulation of histone modification | 0.001646108 | 4 |
| BP | GO:0044344 | cellular response to fibroblast growth factor stimulus | 0.001646108 | 4 |
| BP | GO:0106106 | cold-induced thermogenesis | 0.001646108 | 4 |
| BP | GO:0120161 | regulation of cold-induced thermogenesis | 0.001646108 | 4 |
| BP | GO:0040014 | regulation of multicellular organism growth | 0.001647004 | 3 |
| BP | GO:0051926 | negative regulation of calcium ion transport | 0.001647004 | 3 |
| BP | GO:0070613 | regulation of protein processing | 0.001647004 | 3 |
| BP | GO:1905207 | regulation of cardiocyte differentiation | 0.001647004 | 3 |
| BP | GO:0001654 | eye development | 0.001740167 | 6 |
| BP | GO:0006936 | muscle contraction | 0.00176457 | 6 |
| BP | GO:0002224 | toll-like receptor signaling pathway | 0.00177613 | 4 |
| BP | GO:0002292 | T cell differentiation involved in immune response | 0.001794742 | 3 |
| BP | GO:0010812 | negative regulation of cell-substrate adhesion | 0.001794742 | 3 |
| BP | GO:1903317 | regulation of protein maturation | 0.001794742 | 3 |
| BP | GO:0007252 | I-kappaB phosphorylation | 0.00181557 | 2 |
| BP | GO:0035994 | response to muscle stretch | 0.00181557 | 2 |
| BP | GO:0044320 | cellular response to leptin stimulus | 0.00181557 | 2 |
| BP | GO:0045780 | positive regulation of bone resorption | 0.00181557 | 2 |
| BP | GO:0046852 | positive regulation of bone remodeling | 0.00181557 | 2 |
| BP | GO:0051023 | regulation of immunoglobulin secretion | 0.00181557 | 2 |
| BP | GO:0090330 | regulation of platelet aggregation | 0.00181557 | 2 |
| BP | GO:1900543 | negative regulation of purine nucleotide metabolic process | 0.00181557 | 2 |
| BP | GO:2000757 | negative regulation of peptidyl-lysine acetylation | 0.00181557 | 2 |
| BP | GO:0150063 | visual system development | 0.001839344 | 6 |
| BP | GO:0042119 | neutrophil activation | 0.001843497 | 7 |
| BP | GO:0035148 | tube formation | 0.001866672 | 4 |
| BP | GO:0008088 | axo-dendritic transport | 0.001871612 | 3 |
| BP | GO:0015909 | long-chain fatty acid transport | 0.001871612 | 3 |
| BP | GO:0050766 | positive regulation of phagocytosis | 0.001871612 | 3 |
| BP | GO:0070227 | lymphocyte apoptotic process | 0.001871612 | 3 |
| BP | GO:0060041 | retina development in camera-type eye | 0.001913123 | 4 |
| BP | GO:0045017 | glycerolipid biosynthetic process | 0.001934734 | 5 |
| BP | GO:0032535 | regulation of cellular component size | 0.001942763 | 6 |
| BP | GO:0007004 | telomere maintenance via telomerase | 0.001950509 | 3 |
| BP | GO:0033077 | T cell differentiation in thymus | 0.001950509 | 3 |
| BP | GO:0061515 | myeloid cell development | 0.001950509 | 3 |
| BP | GO:0008360 | regulation of cell shape | 0.001960369 | 4 |
| BP | GO:0050777 | negative regulation of immune response | 0.001960369 | 4 |
| BP | GO:0071774 | response to fibroblast growth factor | 0.001960369 | 4 |
| BP | GO:0048880 | sensory system development | 0.001969295 | 6 |
| BP | GO:2000027 | regulation of animal organ morphogenesis | 0.002002848 | 5 |
| BP | GO:0002363 | alpha-beta T cell lineage commitment | 0.002024547 | 2 |
| BP | GO:0002922 | positive regulation of humoral immune response | 0.002024547 | 2 |
| BP | GO:0032026 | response to magnesium ion | 0.002024547 | 2 |
| BP | GO:0032095 | regulation of response to food | 0.002024547 | 2 |
| BP | GO:0042789 | mRNA transcription by RNA polymerase II | 0.002024547 | 2 |
| BP | GO:0045980 | negative regulation of nucleotide metabolic process | 0.002024547 | 2 |
| BP | GO:0051546 | keratinocyte migration | 0.002024547 | 2 |
| BP | GO:0060149 | negative regulation of posttranscriptional gene silencing | 0.002024547 | 2 |
| BP | GO:0060252 | positive regulation of glial cell proliferation | 0.002024547 | 2 |
| BP | GO:0060716 | labyrinthine layer blood vessel development | 0.002024547 | 2 |
| BP | GO:0060967 | negative regulation of gene silencing by RNA | 0.002024547 | 2 |
| BP | GO:0072074 | kidney mesenchyme development | 0.002024547 | 2 |
| BP | GO:1900409 | positive regulation of cellular response to oxidative stress | 0.002024547 | 2 |
| BP | GO:2000010 | positive regulation of protein localization to cell surface | 0.002024547 | 2 |
| BP | GO:2000251 | positive regulation of actin cytoskeleton reorganization | 0.002024547 | 2 |
| BP | GO:2000696 | regulation of epithelial cell differentiation involved in kidney development | 0.002024547 | 2 |
| BP | GO:1904427 | positive regulation of calcium ion transmembrane transport | 0.002031451 | 3 |
| BP | GO:0016202 | regulation of striated muscle tissue development | 0.002057278 | 4 |
| BP | GO:0030902 | hindbrain development | 0.002057278 | 4 |
| BP | GO:0051017 | actin filament bundle assembly | 0.002106954 | 4 |
| BP | GO:0090596 | sensory organ morphogenesis | 0.002108292 | 5 |
| BP | GO:0006635 | fatty acid beta-oxidation | 0.002114457 | 3 |
| BP | GO:0035019 | somatic stem cell population maintenance | 0.002114457 | 3 |
| BP | GO:0050805 | negative regulation of synaptic transmission | 0.002114457 | 3 |
| BP | GO:1903747 | regulation of establishment of protein localization to mitochondrion | 0.002114457 | 3 |
| BP | GO:0010770 | positive regulation of cell morphogenesis involved in differentiation | 0.002157453 | 4 |
| BP | GO:1990845 | adaptive thermogenesis | 0.002157453 | 4 |
| BP | GO:0032945 | negative regulation of mononuclear cell proliferation | 0.002199545 | 3 |
| BP | GO:0045685 | regulation of glial cell differentiation | 0.002199545 | 3 |
| BP | GO:0050672 | negative regulation of lymphocyte proliferation | 0.002199545 | 3 |
| BP | GO:1901983 | regulation of protein acetylation | 0.002199545 | 3 |
| BP | GO:1901861 | regulation of muscle tissue development | 0.002208784 | 4 |
| BP | GO:1903707 | negative regulation of hemopoiesis | 0.002208784 | 4 |
| BP | GO:0006525 | arginine metabolic process | 0.002244377 | 2 |
| BP | GO:0030220 | platelet formation | 0.002244377 | 2 |
| BP | GO:0033194 | response to hydroperoxide | 0.002244377 | 2 |
| BP | GO:0043369 | CD4-positive or CD8-positive, alpha-beta T cell lineage commitment | 0.002244377 | 2 |
| BP | GO:0043586 | tongue development | 0.002244377 | 2 |
| BP | GO:0060008 | Sertoli cell differentiation | 0.002244377 | 2 |
| BP | GO:1905523 | positive regulation of macrophage migration | 0.002244377 | 2 |
| BP | GO:0008654 | phospholipid biosynthetic process | 0.002255131 | 5 |
| BP | GO:0072659 | protein localization to plasma membrane | 0.002255131 | 5 |
| BP | GO:0030856 | regulation of epithelial cell differentiation | 0.002260953 | 4 |
| BP | GO:0048634 | regulation of muscle organ development | 0.002260953 | 4 |
| BP | GO:0014015 | positive regulation of gliogenesis | 0.002286734 | 3 |
| BP | GO:0031670 | cellular response to nutrient | 0.002286734 | 3 |
| BP | GO:0043367 | CD4-positive, alpha-beta T cell differentiation | 0.002286734 | 3 |
| BP | GO:0070373 | negative regulation of ERK1 and ERK2 cascade | 0.002286734 | 3 |
| BP | GO:0061572 | actin filament bundle organization | 0.002313966 | 4 |
| BP | GO:0007422 | peripheral nervous system development | 0.002376041 | 3 |
| BP | GO:0033143 | regulation of intracellular steroid hormone receptor signaling pathway | 0.002376041 | 3 |
| BP | GO:0001937 | negative regulation of endothelial cell proliferation | 0.002467484 | 3 |
| BP | GO:0046902 | regulation of mitochondrial membrane permeability | 0.002467484 | 3 |
| BP | GO:0001779 | natural killer cell differentiation | 0.002474983 | 2 |
| BP | GO:0010310 | regulation of hydrogen peroxide metabolic process | 0.002474983 | 2 |
| BP | GO:0021697 | cerebellar cortex formation | 0.002474983 | 2 |
| BP | GO:0035357 | peroxisome proliferator activated receptor signaling pathway | 0.002474983 | 2 |
| BP | GO:0036344 | platelet morphogenesis | 0.002474983 | 2 |
| BP | GO:0045061 | thymic T cell selection | 0.002474983 | 2 |
| BP | GO:0046827 | positive regulation of protein export from nucleus | 0.002474983 | 2 |
| BP | GO:0055093 | response to hyperoxia | 0.002474983 | 2 |
| BP | GO:0072111 | cell proliferation involved in kidney development | 0.002474983 | 2 |
| BP | GO:1902884 | positive regulation of response to oxidative stress | 0.002474983 | 2 |
| BP | GO:2000269 | regulation of fibroblast apoptotic process | 0.002474983 | 2 |
| BP | GO:2000810 | regulation of bicellular tight junction assembly | 0.002474983 | 2 |
| BP | GO:0002819 | regulation of adaptive immune response | 0.002478144 | 4 |
| BP | GO:0021915 | neural tube development | 0.002478144 | 4 |
| BP | GO:1901617 | organic hydroxy compound biosynthetic process | 0.002489207 | 5 |
| BP | GO:0014706 | striated muscle tissue development | 0.002527612 | 6 |
| BP | GO:0006278 | RNA-dependent DNA biosynthetic process | 0.002561081 | 3 |
| BP | GO:0031397 | negative regulation of protein ubiquitination | 0.002561081 | 3 |
| BP | GO:0048678 | response to axon injury | 0.002561081 | 3 |
| BP | GO:0051702 | interaction with symbiont | 0.002561081 | 3 |
| BP | GO:0010639 | negative regulation of organelle organization | 0.002625658 | 6 |
| BP | GO:0031214 | biomineral tissue development | 0.002650174 | 4 |
| BP | GO:0110148 | biomineralization | 0.002650174 | 4 |
| BP | GO:0001910 | regulation of leukocyte mediated cytotoxicity | 0.002656847 | 3 |
| BP | GO:0021954 | central nervous system neuron development | 0.002656847 | 3 |
| BP | GO:0070664 | negative regulation of leukocyte proliferation | 0.002656847 | 3 |
| BP | GO:0045165 | cell fate commitment | 0.002697395 | 5 |
| BP | GO:0006633 | fatty acid biosynthetic process | 0.002709294 | 4 |
| BP | GO:0032727 | positive regulation of interferon-alpha production | 0.002716288 | 2 |
| BP | GO:0034643 | establishment of mitochondrion localization, microtubule-mediated | 0.002716288 | 2 |
| BP | GO:0042359 | vitamin D metabolic process | 0.002716288 | 2 |
| BP | GO:0044321 | response to leptin | 0.002716288 | 2 |
| BP | GO:0045943 | positive regulation of transcription by RNA polymerase I | 0.002716288 | 2 |
| BP | GO:0046628 | positive regulation of insulin receptor signaling pathway | 0.002716288 | 2 |
| BP | GO:0047497 | mitochondrion transport along microtubule | 0.002716288 | 2 |
| BP | GO:0048305 | immunoglobulin secretion | 0.002716288 | 2 |
| BP | GO:0071404 | cellular response to low-density lipoprotein particle stimulus | 0.002716288 | 2 |
| BP | GO:1901984 | negative regulation of protein acetylation | 0.002716288 | 2 |
| BP | GO:2000773 | negative regulation of cellular senescence | 0.002716288 | 2 |
| BP | GO:0001570 | vasculogenesis | 0.002754801 | 3 |
| BP | GO:0050680 | negative regulation of epithelial cell proliferation | 0.002769314 | 4 |
| BP | GO:0050954 | sensory perception of mechanical stimulus | 0.002769314 | 4 |
| BP | GO:0006475 | internal protein amino acid acetylation | 0.002830241 | 4 |
| BP | GO:0009150 | purine ribonucleotide metabolic process | 0.002830319 | 6 |
| BP | GO:0016054 | organic acid catabolic process | 0.002872835 | 5 |
| BP | GO:0046395 | carboxylic acid catabolic process | 0.002872835 | 5 |
| BP | GO:0014032 | neural crest cell development | 0.002957336 | 3 |
| BP | GO:0022617 | extracellular matrix disassembly | 0.002957336 | 3 |
| BP | GO:1902930 | regulation of alcohol biosynthetic process | 0.002957336 | 3 |
| BP | GO:0006359 | regulation of transcription by RNA polymerase III | 0.002968217 | 2 |
| BP | GO:0030539 | male genitalia development | 0.002968217 | 2 |
| BP | GO:0034695 | response to prostaglandin E | 0.002968217 | 2 |
| BP | GO:0045821 | positive regulation of glycolytic process | 0.002968217 | 2 |
| BP | GO:0048714 | positive regulation of oligodendrocyte differentiation | 0.002968217 | 2 |
| BP | GO:0051152 | positive regulation of smooth muscle cell differentiation | 0.002968217 | 2 |
| BP | GO:0051349 | positive regulation of lyase activity | 0.002968217 | 2 |
| BP | GO:0051882 | mitochondrial depolarization | 0.002968217 | 2 |
| BP | GO:0060055 | angiogenesis involved in wound healing | 0.002968217 | 2 |
| BP | GO:0072215 | regulation of metanephros development | 0.002968217 | 2 |
| BP | GO:0090140 | regulation of mitochondrial fission | 0.002968217 | 2 |
| BP | GO:1902254 | negative regulation of intrinsic apoptotic signaling pathway by p53 class mediator | 0.002968217 | 2 |
| BP | GO:0021543 | pallium development | 0.003018529 | 4 |
| BP | GO:0006094 | gluconeogenesis | 0.00306195 | 3 |
| BP | GO:0010833 | telomere maintenance via telomere lengthening | 0.00306195 | 3 |
| BP | GO:0090049 | regulation of cell migration involved in sprouting angiogenesis | 0.00306195 | 3 |
| BP | GO:1905954 | positive regulation of lipid localization | 0.00306195 | 3 |
| BP | GO:0006937 | regulation of muscle contraction | 0.003148712 | 4 |
| BP | GO:0060537 | muscle tissue development | 0.003159514 | 6 |
| BP | GO:0009110 | vitamin biosynthetic process | 0.003230692 | 2 |
| BP | GO:0044346 | fibroblast apoptotic process | 0.003230692 | 2 |
| BP | GO:0050996 | positive regulation of lipid catabolic process | 0.003230692 | 2 |
| BP | GO:0071677 | positive regulation of mononuclear cell migration | 0.003230692 | 2 |
| BP | GO:1900078 | positive regulation of cellular response to insulin stimulus | 0.003230692 | 2 |
| BP | GO:2000209 | regulation of anoikis | 0.003230692 | 2 |
| BP | GO:2001026 | regulation of endothelial cell chemotaxis | 0.003230692 | 2 |
| BP | GO:0014910 | regulation of smooth muscle cell migration | 0.003277948 | 3 |
| BP | GO:1905897 | regulation of response to endoplasmic reticulum stress | 0.003277948 | 3 |
| BP | GO:0055002 | striated muscle cell development | 0.003282683 | 4 |
| BP | GO:0051604 | protein maturation | 0.003347491 | 5 |
| BP | GO:0014031 | mesenchymal cell development | 0.003389364 | 3 |
| BP | GO:0019319 | hexose biosynthetic process | 0.003389364 | 3 |
| BP | GO:0050772 | positive regulation of axonogenesis | 0.003389364 | 3 |
| BP | GO:0009259 | ribonucleotide metabolic process | 0.003394389 | 6 |
| BP | GO:0002695 | negative regulation of leukocyte activation | 0.003420496 | 4 |
| BP | GO:0046165 | alcohol biosynthetic process | 0.003420496 | 4 |
| BP | GO:0042446 | hormone biosynthetic process | 0.003503078 | 3 |
| BP | GO:0009299 | mRNA transcription | 0.00350364 | 2 |
| BP | GO:0019430 | removal of superoxide radicals | 0.00350364 | 2 |
| BP | GO:0032104 | regulation of response to extracellular stimulus | 0.00350364 | 2 |
| BP | GO:0032107 | regulation of response to nutrient levels | 0.00350364 | 2 |
| BP | GO:0034110 | regulation of homotypic cell-cell adhesion | 0.00350364 | 2 |
| BP | GO:0044068 | modulation by symbiont of host cellular process | 0.00350364 | 2 |
| BP | GO:0045830 | positive regulation of isotype switching | 0.00350364 | 2 |
| BP | GO:0051654 | establishment of mitochondrion localization | 0.00350364 | 2 |
| BP | GO:0051894 | positive regulation of focal adhesion assembly | 0.00350364 | 2 |
| BP | GO:0060444 | branching involved in mammary gland duct morphogenesis | 0.00350364 | 2 |
| BP | GO:0061050 | regulation of cell growth involved in cardiac muscle cell development | 0.00350364 | 2 |
| BP | GO:0072012 | glomerulus vasculature development | 0.00350364 | 2 |
| BP | GO:0072273 | metanephric nephron morphogenesis | 0.00350364 | 2 |
| BP | GO:1900101 | regulation of endoplasmic reticulum unfolded protein response | 0.00350364 | 2 |
| BP | GO:0006941 | striated muscle contraction | 0.003562203 | 4 |
| BP | GO:0007416 | synapse assembly | 0.003562203 | 4 |
| BP | GO:0043488 | regulation of mRNA stability | 0.003562203 | 4 |
| BP | GO:0002690 | positive regulation of leukocyte chemotaxis | 0.003619105 | 3 |
| BP | GO:0045844 | positive regulation of striated muscle tissue development | 0.003619105 | 3 |
| BP | GO:0048636 | positive regulation of muscle organ development | 0.003619105 | 3 |
| BP | GO:0050821 | protein stabilization | 0.003634533 | 4 |
| BP | GO:0008361 | regulation of cell size | 0.003707857 | 4 |
| BP | GO:0009791 | post-embryonic development | 0.003737459 | 3 |
| BP | GO:0034103 | regulation of tissue remodeling | 0.003737459 | 3 |
| BP | GO:0061097 | regulation of protein tyrosine kinase activity | 0.003737459 | 3 |
| BP | GO:0097194 | execution phase of apoptosis | 0.003737459 | 3 |
| BP | GO:1901863 | positive regulation of muscle tissue development | 0.003737459 | 3 |
| BP | GO:1903321 | negative regulation of protein modification by small protein conjugation or removal | 0.003737459 | 3 |
| BP | GO:0071466 | cellular response to xenobiotic stimulus | 0.00378218 | 4 |
| BP | GO:1901796 | regulation of signal transduction by p53 class mediator | 0.00378218 | 4 |
| BP | GO:0002360 | T cell lineage commitment | 0.003786985 | 2 |
| BP | GO:0010758 | regulation of macrophage chemotaxis | 0.003786985 | 2 |
| BP | GO:0022011 | myelination in peripheral nervous system | 0.003786985 | 2 |
| BP | GO:0032292 | peripheral nervous system axon ensheathment | 0.003786985 | 2 |
| BP | GO:0032967 | positive regulation of collagen biosynthetic process | 0.003786985 | 2 |
| BP | GO:0055003 | cardiac myofibril assembly | 0.003786985 | 2 |
| BP | GO:0060706 | cell differentiation involved in embryonic placenta development | 0.003786985 | 2 |
| BP | GO:0060740 | prostate gland epithelium morphogenesis | 0.003786985 | 2 |
| BP | GO:0072539 | T-helper 17 cell differentiation | 0.003786985 | 2 |
| BP | GO:0150117 | positive regulation of cell-substrate junction organization | 0.003786985 | 2 |
| BP | GO:1900739 | regulation of protein insertion into mitochondrial membrane involved in apoptotic signaling pathway | 0.003786985 | 2 |
| BP | GO:1900740 | positive regulation of protein insertion into mitochondrial membrane involved in apoptotic signaling pathway | 0.003786985 | 2 |
| BP | GO:1902473 | regulation of protein localization to synapse | 0.003786985 | 2 |
| BP | GO:1903203 | regulation of oxidative stress-induced neuron death | 0.003786985 | 2 |
| BP | GO:1903959 | regulation of anion transmembrane transport | 0.003786985 | 2 |
| BP | GO:1904353 | regulation of telomere capping | 0.003786985 | 2 |
| BP | GO:0019693 | ribose phosphate metabolic process | 0.003814549 | 6 |
| BP | GO:0050768 | negative regulation of neurogenesis | 0.003876331 | 5 |
| BP | GO:0030041 | actin filament polymerization | 0.003933852 | 4 |
| BP | GO:0001656 | metanephros development | 0.003981206 | 3 |
| BP | GO:0001843 | neural tube closure | 0.003981206 | 3 |
| BP | GO:0014033 | neural crest cell differentiation | 0.003981206 | 3 |
| BP | GO:0042475 | odontogenesis of dentin-containing tooth | 0.003981206 | 3 |
| BP | GO:0043487 | regulation of RNA stability | 0.004011214 | 4 |
| BP | GO:0050770 | regulation of axonogenesis | 0.004011214 | 4 |
| BP | GO:0010714 | positive regulation of collagen metabolic process | 0.004080651 | 2 |
| BP | GO:0035902 | response to immobilization stress | 0.004080651 | 2 |
| BP | GO:0036475 | neuron death in response to oxidative stress | 0.004080651 | 2 |
| BP | GO:0045671 | negative regulation of osteoclast differentiation | 0.004080651 | 2 |
| BP | GO:0045932 | negative regulation of muscle contraction | 0.004080651 | 2 |
| BP | GO:0046885 | regulation of hormone biosynthetic process | 0.004080651 | 2 |
| BP | GO:0048668 | collateral sprouting | 0.004080651 | 2 |
| BP | GO:0060259 | regulation of feeding behavior | 0.004080651 | 2 |
| BP | GO:0061437 | renal system vasculature development | 0.004080651 | 2 |
| BP | GO:0061440 | kidney vasculature development | 0.004080651 | 2 |
| BP | GO:0071280 | cellular response to copper ion | 0.004080651 | 2 |
| BP | GO:0071450 | cellular response to oxygen radical | 0.004080651 | 2 |
| BP | GO:0071451 | cellular response to superoxide | 0.004080651 | 2 |
| BP | GO:1905208 | negative regulation of cardiocyte differentiation | 0.004080651 | 2 |
| BP | GO:2000191 | regulation of fatty acid transport | 0.004080651 | 2 |
| BP | GO:0014909 | smooth muscle cell migration | 0.004106628 | 3 |
| BP | GO:0046364 | monosaccharide biosynthetic process | 0.004106628 | 3 |
| BP | GO:0060606 | tube closure | 0.004106628 | 3 |
| BP | GO:1902275 | regulation of chromatin organization | 0.00416902 | 4 |
| BP | GO:0035710 | CD4-positive, alpha-beta T cell activation | 0.004234433 | 3 |
| BP | GO:0055001 | muscle cell development | 0.004249478 | 4 |
| BP | GO:0070507 | regulation of microtubule cytoskeleton organization | 0.004249478 | 4 |
| BP | GO:0006476 | protein deacetylation | 0.004364636 | 3 |
| BP | GO:0032092 | positive regulation of protein binding | 0.004364636 | 3 |
| BP | GO:1903351 | cellular response to dopamine | 0.004364636 | 3 |
| BP | GO:0008209 | androgen metabolic process | 0.004384566 | 2 |
| BP | GO:0009065 | glutamine family amino acid catabolic process | 0.004384566 | 2 |
| BP | GO:0010818 | T cell chemotaxis | 0.004384566 | 2 |
| BP | GO:0032647 | regulation of interferon-alpha production | 0.004384566 | 2 |
| BP | GO:0033598 | mammary gland epithelial cell proliferation | 0.004384566 | 2 |
| BP | GO:0034377 | plasma lipoprotein particle assembly | 0.004384566 | 2 |
| BP | GO:0036296 | response to increased oxygen levels | 0.004384566 | 2 |
| BP | GO:0060512 | prostate gland morphogenesis | 0.004384566 | 2 |
| BP | GO:0090344 | negative regulation of cell aging | 0.004384566 | 2 |
| BP | GO:1902932 | positive regulation of alcohol biosynthetic process | 0.004384566 | 2 |
| BP | GO:1903792 | negative regulation of anion transport | 0.004384566 | 2 |
| BP | GO:1904994 | regulation of leukocyte adhesion to vascular endothelial cell | 0.004384566 | 2 |
| BP | GO:1990776 | response to angiotensin | 0.004384566 | 2 |
| BP | GO:1903350 | response to dopamine | 0.004497248 | 3 |
| BP | GO:0050810 | regulation of steroid biosynthetic process | 0.004632285 | 3 |
| BP | GO:0060337 | type I interferon signaling pathway | 0.004632285 | 3 |
| BP | GO:0071357 | cellular response to type I interferon | 0.004632285 | 3 |
| BP | GO:0000303 | response to superoxide | 0.004698655 | 2 |
| BP | GO:0005979 | regulation of glycogen biosynthetic process | 0.004698655 | 2 |
| BP | GO:0010575 | positive regulation of vascular endothelial growth factor production | 0.004698655 | 2 |
| BP | GO:0010800 | positive regulation of peptidyl-threonine phosphorylation | 0.004698655 | 2 |
| BP | GO:0010962 | regulation of glucan biosynthetic process | 0.004698655 | 2 |
| BP | GO:0014044 | Schwann cell development | 0.004698655 | 2 |
| BP | GO:0031116 | positive regulation of microtubule polymerization | 0.004698655 | 2 |
| BP | GO:0035666 | TRIF-dependent toll-like receptor signaling pathway | 0.004698655 | 2 |
| BP | GO:0050974 | detection of mechanical stimulus involved in sensory perception | 0.004698655 | 2 |
| BP | GO:0070229 | negative regulation of lymphocyte apoptotic process | 0.004698655 | 2 |
| BP | GO:0072538 | T-helper 17 type immune response | 0.004698655 | 2 |
| BP | GO:1900027 | regulation of ruffle assembly | 0.004698655 | 2 |
| BP | GO:0006164 | purine nucleotide biosynthetic process | 0.004754381 | 4 |
| BP | GO:1901990 | regulation of mitotic cell cycle phase transition | 0.004769611 | 6 |
| BP | GO:0010660 | regulation of muscle cell apoptotic process | 0.004769757 | 3 |
| BP | GO:0090277 | positive regulation of peptide hormone secretion | 0.004769757 | 3 |
| BP | GO:0097327 | response to antineoplastic agent | 0.004769757 | 3 |
| BP | GO:0044282 | small molecule catabolic process | 0.004821596 | 6 |
| BP | GO:0120162 | positive regulation of cold-induced thermogenesis | 0.004909678 | 3 |
| BP | GO:2001022 | positive regulation of response to DNA damage stimulus | 0.004909678 | 3 |
| BP | GO:0045333 | cellular respiration | 0.004931273 | 4 |
| BP | GO:0000305 | response to oxygen radical | 0.005022844 | 2 |
| BP | GO:0001844 | protein insertion into mitochondrial membrane involved in apoptotic signaling pathway | 0.005022844 | 2 |
| BP | GO:0021696 | cerebellar cortex morphogenesis | 0.005022844 | 2 |
| BP | GO:0032607 | interferon-alpha production | 0.005022844 | 2 |
| BP | GO:0032728 | positive regulation of interferon-beta production | 0.005022844 | 2 |
| BP | GO:0032743 | positive regulation of interleukin-2 production | 0.005022844 | 2 |
| BP | GO:0038111 | interleukin-7-mediated signaling pathway | 0.005022844 | 2 |
| BP | GO:0042759 | long-chain fatty acid biosynthetic process | 0.005022844 | 2 |
| BP | GO:0045948 | positive regulation of translational initiation | 0.005022844 | 2 |
| BP | GO:0048873 | homeostasis of number of cells within a tissue | 0.005022844 | 2 |
| BP | GO:0070168 | negative regulation of biomineral tissue development | 0.005022844 | 2 |
| BP | GO:0090314 | positive regulation of protein targeting to membrane | 0.005022844 | 2 |
| BP | GO:0110150 | negative regulation of biomineralization | 0.005022844 | 2 |
| BP | GO:1905606 | regulation of presynapse assembly | 0.005022844 | 2 |
| BP | GO:0006694 | steroid biosynthetic process | 0.005112543 | 4 |
| BP | GO:0034340 | response to type I interferon | 0.005196915 | 3 |
| BP | GO:0002221 | pattern recognition receptor signaling pathway | 0.005204835 | 4 |
| BP | GO:0007626 | locomotory behavior | 0.005298239 | 4 |
| BP | GO:0031099 | regeneration | 0.005298239 | 4 |
| BP | GO:0016570 | histone modification | 0.005308493 | 6 |
| BP | GO:0002824 | positive regulation of adaptive immune response based on somatic recombination of immune receptors built from immunoglobulin superfamily domains | 0.005344256 | 3 |
| BP | GO:0010657 | muscle cell apoptotic process | 0.005344256 | 3 |
| BP | GO:0060079 | excitatory postsynaptic potential | 0.005344256 | 3 |
| BP | GO:0120034 | positive regulation of plasma membrane bounded cell projection assembly | 0.005344256 | 3 |
| BP | GO:0001963 | synaptic transmission, dopaminergic | 0.005357061 | 2 |
| BP | GO:0007202 | activation of phospholipase C activity | 0.005357061 | 2 |
| BP | GO:0035767 | endothelial cell chemotaxis | 0.005357061 | 2 |
| BP | GO:0045737 | positive regulation of cyclin-dependent protein serine/threonine kinase activity | 0.005357061 | 2 |
| BP | GO:0045742 | positive regulation of epidermal growth factor receptor signaling pathway | 0.005357061 | 2 |
| BP | GO:0099174 | regulation of presynapse organization | 0.005357061 | 2 |
| BP | GO:1902253 | regulation of intrinsic apoptotic signaling pathway by p53 class mediator | 0.005357061 | 2 |
| BP | GO:0043010 | camera-type eye development | 0.005386567 | 5 |
| BP | GO:0061013 | regulation of mRNA catabolic process | 0.005392762 | 4 |
| BP | GO:0048259 | regulation of receptor-mediated endocytosis | 0.005494093 | 3 |
| BP | GO:0000079 | regulation of cyclin-dependent protein serine/threonine kinase activity | 0.005646439 | 3 |
| BP | GO:0098869 | cellular oxidant detoxification | 0.005646439 | 3 |
| BP | GO:0009615 | response to virus | 0.005673701 | 5 |
| BP | GO:0003338 | metanephros morphogenesis | 0.005701232 | 2 |
| BP | GO:0006356 | regulation of transcription by RNA polymerase I | 0.005701232 | 2 |
| BP | GO:0008210 | estrogen metabolic process | 0.005701232 | 2 |
| BP | GO:0045922 | negative regulation of fatty acid metabolic process | 0.005701232 | 2 |
| BP | GO:0045987 | positive regulation of smooth muscle contraction | 0.005701232 | 2 |
| BP | GO:0065005 | protein-lipid complex assembly | 0.005701232 | 2 |
| BP | GO:0071295 | cellular response to vitamin | 0.005701232 | 2 |
| BP | GO:0007266 | Rho protein signal transduction | 0.005782161 | 4 |
| BP | GO:0007229 | integrin-mediated signaling pathway | 0.005801305 | 3 |
| BP | GO:0034766 | negative regulation of ion transmembrane transport | 0.005801305 | 3 |
| BP | GO:0035601 | protein deacylation | 0.005801305 | 3 |
| BP | GO:0006473 | protein acetylation | 0.005882366 | 4 |
| BP | GO:0014812 | muscle cell migration | 0.005958701 | 3 |
| BP | GO:0030593 | neutrophil chemotaxis | 0.005958701 | 3 |
| BP | GO:0098732 | macromolecule deacylation | 0.005958701 | 3 |
| BP | GO:0072522 | purine-containing compound biosynthetic process | 0.005983726 | 4 |
| BP | GO:0002756 | MyD88-independent toll-like receptor signaling pathway | 0.006055286 | 2 |
| BP | GO:0010955 | negative regulation of protein processing | 0.006055286 | 2 |
| BP | GO:0014072 | response to isoquinoline alkaloid | 0.006055286 | 2 |
| BP | GO:0031112 | positive regulation of microtubule polymerization or depolymerization | 0.006055286 | 2 |
| BP | GO:0036003 | positive regulation of transcription from RNA polymerase II promoter in response to stress | 0.006055286 | 2 |
| BP | GO:0042755 | eating behavior | 0.006055286 | 2 |
| BP | GO:0043278 | response to morphine | 0.006055286 | 2 |
| BP | GO:0045191 | regulation of isotype switching | 0.006055286 | 2 |
| BP | GO:0060603 | mammary gland duct morphogenesis | 0.006055286 | 2 |
| BP | GO:1901186 | positive regulation of ERBB signaling pathway | 0.006055286 | 2 |
| BP | GO:1901797 | negative regulation of signal transduction by p53 class mediator | 0.006055286 | 2 |
| BP | GO:1903318 | negative regulation of protein maturation | 0.006055286 | 2 |
| BP | GO:0002708 | positive regulation of lymphocyte mediated immunity | 0.006118639 | 3 |
| BP | GO:0032006 | regulation of TOR signaling | 0.006118639 | 3 |
| BP | GO:1904029 | regulation of cyclin-dependent protein kinase activity | 0.006281129 | 3 |
| BP | GO:2001023 | regulation of response to drug | 0.006281129 | 3 |
| BP | GO:0008154 | actin polymerization or depolymerization | 0.006400822 | 4 |
| BP | GO:0003382 | epithelial cell morphogenesis | 0.00641915 | 2 |
| BP | GO:0007435 | salivary gland morphogenesis | 0.00641915 | 2 |
| BP | GO:0010574 | regulation of vascular endothelial growth factor production | 0.00641915 | 2 |
| BP | GO:0032148 | activation of protein kinase B activity | 0.00641915 | 2 |
| BP | GO:0032212 | positive regulation of telomere maintenance via telomerase | 0.00641915 | 2 |
| BP | GO:0032660 | regulation of interleukin-17 production | 0.00641915 | 2 |
| BP | GO:0043516 | regulation of DNA damage response, signal transduction by p53 class mediator | 0.00641915 | 2 |
| BP | GO:0048566 | embryonic digestive tract development | 0.00641915 | 2 |
| BP | GO:0060969 | negative regulation of gene silencing | 0.00641915 | 2 |
| BP | GO:0070884 | regulation of calcineurin-NFAT signaling cascade | 0.00641915 | 2 |
| BP | GO:0097009 | energy homeostasis | 0.00641915 | 2 |
| BP | GO:0106056 | regulation of calcineurin-mediated signaling | 0.00641915 | 2 |
| BP | GO:0043502 | regulation of muscle adaptation | 0.006446182 | 3 |
| BP | GO:0016569 | covalent chromatin modification | 0.006518957 | 6 |
| BP | GO:0009062 | fatty acid catabolic process | 0.006613808 | 3 |
| BP | GO:0099565 | chemical synaptic transmission, postsynaptic | 0.006613808 | 3 |
| BP | GO:0000266 | mitochondrial fission | 0.006792753 | 2 |
| BP | GO:0003203 | endocardial cushion morphogenesis | 0.006792753 | 2 |
| BP | GO:0019433 | triglyceride catabolic process | 0.006792753 | 2 |
| BP | GO:0060251 | regulation of glial cell proliferation | 0.006792753 | 2 |
| BP | GO:0070423 | nucleotide-binding oligomerization domain containing signaling pathway | 0.006792753 | 2 |
| BP | GO:0070873 | regulation of glycogen metabolic process | 0.006792753 | 2 |
| BP | GO:0098751 | bone cell development | 0.006792753 | 2 |
| BP | GO:0110111 | negative regulation of animal organ morphogenesis | 0.006792753 | 2 |
| BP | GO:1901030 | positive regulation of mitochondrial outer membrane permeabilization involved in apoptotic signaling pathway | 0.006792753 | 2 |
| BP | GO:1904031 | positive regulation of cyclin-dependent protein kinase activity | 0.006792753 | 2 |
| BP | GO:0045930 | negative regulation of mitotic cell cycle | 0.006847264 | 5 |
| BP | GO:0043087 | regulation of GTPase activity | 0.006850678 | 6 |
| BP | GO:1901987 | regulation of cell cycle phase transition | 0.006918472 | 6 |
| BP | GO:0045732 | positive regulation of protein catabolic process | 0.006948849 | 4 |
| BP | GO:0016101 | diterpenoid metabolic process | 0.006956819 | 3 |
| BP | GO:0009112 | nucleobase metabolic process | 0.007176024 | 2 |
| BP | GO:0010573 | vascular endothelial growth factor production | 0.007176024 | 2 |
| BP | GO:0032733 | positive regulation of interleukin-10 production | 0.007176024 | 2 |
| BP | GO:0034390 | smooth muscle cell apoptotic process | 0.007176024 | 2 |
| BP | GO:0034391 | regulation of smooth muscle cell apoptotic process | 0.007176024 | 2 |
| BP | GO:0035872 | nucleotide-binding domain, leucine rich repeat containing receptor signaling pathway | 0.007176024 | 2 |
| BP | GO:0045740 | positive regulation of DNA replication | 0.007176024 | 2 |
| BP | GO:0071392 | cellular response to estradiol stimulus | 0.007176024 | 2 |
| BP | GO:0071634 | regulation of transforming growth factor beta production | 0.007176024 | 2 |
| BP | GO:0090313 | regulation of protein targeting to membrane | 0.007176024 | 2 |
| BP | GO:0097242 | amyloid-beta clearance | 0.007176024 | 2 |
| BP | GO:1901099 | negative regulation of signal transduction in absence of ligand | 0.007176024 | 2 |
| BP | GO:2001240 | negative regulation of extrinsic apoptotic signaling pathway in absence of ligand | 0.007176024 | 2 |
| BP | GO:0000075 | cell cycle checkpoint | 0.007176497 | 4 |
| BP | GO:0002065 | columnar/cuboidal epithelial cell differentiation | 0.00731024 | 3 |
| BP | GO:0015918 | sterol transport | 0.00731024 | 3 |
| BP | GO:0042303 | molting cycle | 0.00731024 | 3 |
| BP | GO:0042633 | hair cycle | 0.00731024 | 3 |
| BP | GO:0050868 | negative regulation of T cell activation | 0.00731024 | 3 |
| BP | GO:1990748 | cellular detoxification | 0.00731024 | 3 |
| BP | GO:0016064 | immunoglobulin mediated immune response | 0.007409032 | 4 |
| BP | GO:0021987 | cerebral cortex development | 0.007490878 | 3 |
| BP | GO:0043583 | ear development | 0.007527146 | 4 |
| BP | GO:0007431 | salivary gland development | 0.007568891 | 2 |
| BP | GO:0014037 | Schwann cell differentiation | 0.007568891 | 2 |
| BP | GO:0032885 | regulation of polysaccharide biosynthetic process | 0.007568891 | 2 |
| BP | GO:0043243 | positive regulation of protein-containing complex disassembly | 0.007568891 | 2 |
| BP | GO:0045730 | respiratory burst | 0.007568891 | 2 |
| BP | GO:0046006 | regulation of activated T cell proliferation | 0.007568891 | 2 |
| BP | GO:0051193 | regulation of cofactor metabolic process | 0.007568891 | 2 |
| BP | GO:0098926 | postsynaptic signal transduction | 0.007568891 | 2 |
| BP | GO:1904358 | positive regulation of telomere maintenance via telomere lengthening | 0.007568891 | 2 |
| BP | GO:0002688 | regulation of leukocyte chemotaxis | 0.007674147 | 3 |
| BP | GO:0008543 | fibroblast growth factor receptor signaling pathway | 0.007674147 | 3 |
| BP | GO:0019724 | B cell mediated immunity | 0.007767093 | 4 |
| BP | GO:0007178 | transmembrane receptor protein serine/threonine kinase signaling pathway | 0.007809596 | 5 |
| BP | GO:0003298 | physiological muscle hypertrophy | 0.007971284 | 2 |
| BP | GO:0003301 | physiological cardiac muscle hypertrophy | 0.007971284 | 2 |
| BP | GO:0010831 | positive regulation of myotube differentiation | 0.007971284 | 2 |
| BP | GO:0032350 | regulation of hormone metabolic process | 0.007971284 | 2 |
| BP | GO:0032620 | interleukin-17 production | 0.007971284 | 2 |
| BP | GO:0048246 | macrophage chemotaxis | 0.007971284 | 2 |
| BP | GO:0060045 | positive regulation of cardiac muscle cell proliferation | 0.007971284 | 2 |
| BP | GO:0061049 | cell growth involved in cardiac muscle cell development | 0.007971284 | 2 |
| BP | GO:0071604 | transforming growth factor beta production | 0.007971284 | 2 |
| BP | GO:1905898 | positive regulation of response to endoplasmic reticulum stress | 0.007971284 | 2 |
| BP | GO:2000736 | regulation of stem cell differentiation | 0.00804861 | 3 |
| BP | GO:0002449 | lymphocyte mediated immunity | 0.008087626 | 5 |
| BP | GO:0007613 | memory | 0.008239823 | 3 |
| BP | GO:0051153 | regulation of striated muscle cell differentiation | 0.008239823 | 3 |
| BP | GO:0046394 | carboxylic acid biosynthetic process | 0.008372489 | 5 |
| BP | GO:0033146 | regulation of intracellular estrogen receptor signaling pathway | 0.008383133 | 2 |
| BP | GO:0033574 | response to testosterone | 0.008383133 | 2 |
| BP | GO:0045911 | positive regulation of DNA recombination | 0.008383133 | 2 |
| BP | GO:0048713 | regulation of oligodendrocyte differentiation | 0.008383133 | 2 |
| BP | GO:0072595 | maintenance of protein localization in organelle | 0.008383133 | 2 |
| BP | GO:1905521 | regulation of macrophage migration | 0.008383133 | 2 |
| BP | GO:2000648 | positive regulation of stem cell proliferation | 0.008383133 | 2 |
| BP | GO:1990266 | neutrophil migration | 0.0084337 | 3 |
| BP | GO:0016053 | organic acid biosynthetic process | 0.008468975 | 5 |
| BP | GO:0002823 | negative regulation of adaptive immune response based on somatic recombination of immune receptors built from immunoglobulin superfamily domains | 0.008804368 | 2 |
| BP | GO:0042026 | protein refolding | 0.008804368 | 2 |
| BP | GO:0044003 | modulation by symbiont of host process | 0.008804368 | 2 |
| BP | GO:0045124 | regulation of bone resorption | 0.008804368 | 2 |
| BP | GO:0071470 | cellular response to osmotic stress | 0.008804368 | 2 |
| BP | GO:0098760 | response to interleukin-7 | 0.008804368 | 2 |
| BP | GO:0098761 | cellular response to interleukin-7 | 0.008804368 | 2 |
| BP | GO:2000008 | regulation of protein localization to cell surface | 0.008804368 | 2 |
| BP | GO:0002698 | negative regulation of immune effector process | 0.008829481 | 3 |
| BP | GO:0006721 | terpenoid metabolic process | 0.008829481 | 3 |
| BP | GO:0022412 | cellular process involved in reproduction in multicellular organism | 0.008963042 | 5 |
| BP | GO:0001704 | formation of primary germ layer | 0.009031401 | 3 |
| BP | GO:0010811 | positive regulation of cell-substrate adhesion | 0.009031401 | 3 |
| BP | GO:0048675 | axon extension | 0.009031401 | 3 |
| BP | GO:0030111 | regulation of Wnt signaling pathway | 0.009166153 | 5 |
| BP | GO:0006111 | regulation of gluconeogenesis | 0.00923492 | 2 |
| BP | GO:0061028 | establishment of endothelial barrier | 0.00923492 | 2 |
| BP | GO:0097178 | ruffle assembly | 0.00923492 | 2 |
| BP | GO:0140353 | lipid export from cell | 0.00923492 | 2 |
| BP | GO:0016999 | antibiotic metabolic process | 0.009236017 | 3 |
| BP | GO:0002639 | positive regulation of immunoglobulin production | 0.00967472 | 2 |
| BP | GO:0010677 | negative regulation of cellular carbohydrate metabolic process | 0.00967472 | 2 |
| BP | GO:0031057 | negative regulation of histone modification | 0.00967472 | 2 |
| BP | GO:0031641 | regulation of myelination | 0.00967472 | 2 |
| BP | GO:1901031 | regulation of response to reactive oxygen species | 0.00967472 | 2 |
| BP | GO:0072330 | monocarboxylic acid biosynthetic process | 0.009869955 | 4 |
| BP | GO:0006066 | alcohol metabolic process | 0.009902158 | 5 |
| BP | GO:0090101 | negative regulation of transmembrane receptor protein serine/threonine kinase signaling pathway | 0.01008159 | 3 |
| BP | GO:0001953 | negative regulation of cell-matrix adhesion | 0.010123698 | 2 |
| BP | GO:0006775 | fat-soluble vitamin metabolic process | 0.010123698 | 2 |
| BP | GO:0032881 | regulation of polysaccharide metabolic process | 0.010123698 | 2 |
| BP | GO:0033173 | calcineurin-NFAT signaling cascade | 0.010123698 | 2 |
| BP | GO:0045687 | positive regulation of glial cell differentiation | 0.010123698 | 2 |
| BP | GO:0045746 | negative regulation of Notch signaling pathway | 0.010123698 | 2 |
| BP | GO:0046461 | neutral lipid catabolic process | 0.010123698 | 2 |
| BP | GO:0046464 | acylglycerol catabolic process | 0.010123698 | 2 |
| BP | GO:0060612 | adipose tissue development | 0.010123698 | 2 |
| BP | GO:0099054 | presynapse assembly | 0.010123698 | 2 |
| BP | GO:0003197 | endocardial cushion development | 0.010581787 | 2 |
| BP | GO:0014047 | glutamate secretion | 0.010581787 | 2 |
| BP | GO:0035850 | epithelial cell differentiation involved in kidney development | 0.010581787 | 2 |
| BP | GO:0050982 | detection of mechanical stimulus | 0.010581787 | 2 |
| BP | GO:0060999 | positive regulation of dendritic spine development | 0.010581787 | 2 |
| BP | GO:0061756 | leukocyte adhesion to vascular endothelial cell | 0.010581787 | 2 |
| BP | GO:0071622 | regulation of granulocyte chemotaxis | 0.010581787 | 2 |
| BP | GO:0090311 | regulation of protein deacetylation | 0.010581787 | 2 |
| BP | GO:1901028 | regulation of mitochondrial outer membrane permeabilization involved in apoptotic signaling pathway | 0.010581787 | 2 |
| BP | GO:0003206 | cardiac chamber morphogenesis | 0.010744426 | 3 |
| BP | GO:0034754 | cellular hormone metabolic process | 0.010744426 | 3 |
| BP | GO:0003231 | cardiac ventricle development | 0.010970864 | 3 |
| BP | GO:0043500 | muscle adaptation | 0.010970864 | 3 |
| BP | GO:0001974 | blood vessel remodeling | 0.011048919 | 2 |
| BP | GO:0002820 | negative regulation of adaptive immune response | 0.011048919 | 2 |
| BP | GO:0032309 | icosanoid secretion | 0.011048919 | 2 |
| BP | GO:0035987 | endodermal cell differentiation | 0.011048919 | 2 |
| BP | GO:0045581 | negative regulation of T cell differentiation | 0.011048919 | 2 |
| BP | GO:0048255 | mRNA stabilization | 0.011048919 | 2 |
| BP | GO:0071827 | plasma lipoprotein particle organization | 0.011048919 | 2 |
| BP | GO:1900271 | regulation of long-term synaptic potentiation | 0.011048919 | 2 |
| BP | GO:0030900 | forebrain development | 0.011139609 | 5 |
| BP | GO:1903362 | regulation of cellular protein catabolic process | 0.011355723 | 4 |
| BP | GO:0031333 | negative regulation of protein-containing complex assembly | 0.011432016 | 3 |
| BP | GO:0042177 | negative regulation of protein catabolic process | 0.011432016 | 3 |
| BP | GO:0042476 | odontogenesis | 0.011432016 | 3 |
| BP | GO:0072329 | monocarboxylic acid catabolic process | 0.011432016 | 3 |
| BP | GO:0043543 | protein acylation | 0.011511788 | 4 |
| BP | GO:0001754 | eye photoreceptor cell differentiation | 0.011525026 | 2 |
| BP | GO:0002437 | inflammatory response to antigenic stimulus | 0.011525026 | 2 |
| BP | GO:0005978 | glycogen biosynthetic process | 0.011525026 | 2 |
| BP | GO:0009250 | glucan biosynthetic process | 0.011525026 | 2 |
| BP | GO:0032369 | negative regulation of lipid transport | 0.011525026 | 2 |
| BP | GO:0043330 | response to exogenous dsRNA | 0.011525026 | 2 |
| BP | GO:0051339 | regulation of lyase activity | 0.011525026 | 2 |
| BP | GO:0051646 | mitochondrion localization | 0.011525026 | 2 |
| BP | GO:0060443 | mammary gland morphogenesis | 0.011525026 | 2 |
| BP | GO:0097720 | calcineurin-mediated signaling | 0.011525026 | 2 |
| BP | GO:0099172 | presynapse organization | 0.011525026 | 2 |
| BP | GO:0021537 | telencephalon development | 0.011669232 | 4 |
| BP | GO:0045926 | negative regulation of growth | 0.011669232 | 4 |
| BP | GO:0002920 | regulation of humoral immune response | 0.01190424 | 3 |
| BP | GO:0044409 | entry into host | 0.01190424 | 3 |
| BP | GO:0002204 | somatic recombination of immunoglobulin genes involved in immune response | 0.01201004 | 2 |
| BP | GO:0002208 | somatic diversification of immunoglobulins involved in immune response | 0.01201004 | 2 |
| BP | GO:0008206 | bile acid metabolic process | 0.01201004 | 2 |
| BP | GO:0010799 | regulation of peptidyl-threonine phosphorylation | 0.01201004 | 2 |
| BP | GO:0014911 | positive regulation of smooth muscle cell migration | 0.01201004 | 2 |
| BP | GO:0018198 | peptidyl-cysteine modification | 0.01201004 | 2 |
| BP | GO:0030574 | collagen catabolic process | 0.01201004 | 2 |
| BP | GO:0035722 | interleukin-12-mediated signaling pathway | 0.01201004 | 2 |
| BP | GO:0042551 | neuron maturation | 0.01201004 | 2 |
| BP | GO:0045190 | isotype switching | 0.01201004 | 2 |
| BP | GO:0046638 | positive regulation of alpha-beta T cell differentiation | 0.01201004 | 2 |
| BP | GO:0070231 | T cell apoptotic process | 0.01201004 | 2 |
| BP | GO:2000107 | negative regulation of leukocyte apoptotic process | 0.01201004 | 2 |
| BP | GO:2001239 | regulation of extrinsic apoptotic signaling pathway in absence of ligand | 0.01201004 | 2 |
| BP | GO:0001889 | liver development | 0.012144518 | 3 |
| BP | GO:0007292 | female gamete generation | 0.012387581 | 3 |
| BP | GO:0002762 | negative regulation of myeloid leukocyte differentiation | 0.012503895 | 2 |
| BP | GO:0007528 | neuromuscular junction development | 0.012503895 | 2 |
| BP | GO:0008038 | neuron recognition | 0.012503895 | 2 |
| BP | GO:0030850 | prostate gland development | 0.012503895 | 2 |
| BP | GO:0032648 | regulation of interferon-beta production | 0.012503895 | 2 |
| BP | GO:0035272 | exocrine system development | 0.012503895 | 2 |
| BP | GO:0045912 | negative regulation of carbohydrate metabolic process | 0.012503895 | 2 |
| BP | GO:0045933 | positive regulation of muscle contraction | 0.012503895 | 2 |
| BP | GO:0046850 | regulation of bone remodeling | 0.012503895 | 2 |
| BP | GO:0051150 | regulation of smooth muscle cell differentiation | 0.012503895 | 2 |
| BP | GO:0072604 | interleukin-6 secretion | 0.012503895 | 2 |
| BP | GO:0090199 | regulation of release of cytochrome c from mitochondria | 0.012503895 | 2 |
| BP | GO:0101023 | vascular endothelial cell proliferation | 0.012503895 | 2 |
| BP | GO:1905562 | regulation of vascular endothelial cell proliferation | 0.012503895 | 2 |
| BP | GO:0031644 | regulation of nervous system process | 0.012633432 | 3 |
| BP | GO:0098754 | detoxification | 0.012633432 | 3 |
| BP | GO:0061008 | hepaticobiliary system development | 0.012882078 | 3 |
| BP | GO:0030857 | negative regulation of epithelial cell differentiation | 0.013006525 | 2 |
| BP | GO:0071349 | cellular response to interleukin-12 | 0.013006525 | 2 |
| BP | GO:0071715 | icosanoid transport | 0.013006525 | 2 |
| BP | GO:0071825 | protein-lipid complex subunit organization | 0.013006525 | 2 |
| BP | GO:1901571 | fatty acid derivative transport | 0.013006525 | 2 |
| BP | GO:0006720 | isoprenoid metabolic process | 0.013133523 | 3 |
| BP | GO:0006839 | mitochondrial transport | 0.013149005 | 4 |
| BP | GO:0003007 | heart morphogenesis | 0.013320476 | 4 |
| BP | GO:0060078 | regulation of postsynaptic membrane potential | 0.013387771 | 3 |
| BP | GO:1903364 | positive regulation of cellular protein catabolic process | 0.013387771 | 3 |
| BP | GO:0001706 | endoderm formation | 0.013517862 | 2 |
| BP | GO:0014009 | glial cell proliferation | 0.013517862 | 2 |
| BP | GO:0032608 | interferon-beta production | 0.013517862 | 2 |
| BP | GO:0043489 | RNA stabilization | 0.013517862 | 2 |
| BP | GO:0070671 | response to interleukin-12 | 0.013517862 | 2 |
| BP | GO:2000725 | regulation of cardiac muscle cell differentiation | 0.013517862 | 2 |
| BP | GO:0007281 | germ cell development | 0.013843455 | 4 |
| BP | GO:0007030 | Golgi organization | 0.013904694 | 3 |
| BP | GO:0008277 | regulation of G protein-coupled receptor signaling pathway | 0.013904694 | 3 |
| BP | GO:0072006 | nephron development | 0.013904694 | 3 |
| BP | GO:1902904 | negative regulation of supramolecular fiber organization | 0.013904694 | 3 |
| BP | GO:0001954 | positive regulation of cell-matrix adhesion | 0.014037841 | 2 |
| BP | GO:0031103 | axon regeneration | 0.014037841 | 2 |
| BP | GO:0032007 | negative regulation of TOR signaling | 0.014037841 | 2 |
| BP | GO:0032206 | positive regulation of telomere maintenance | 0.014037841 | 2 |
| BP | GO:0045599 | negative regulation of fat cell differentiation | 0.014037841 | 2 |
| BP | GO:0045744 | negative regulation of G protein-coupled receptor signaling pathway | 0.014037841 | 2 |
| BP | GO:0048260 | positive regulation of receptor-mediated endocytosis | 0.014037841 | 2 |
| BP | GO:0051496 | positive regulation of stress fiber assembly | 0.014037841 | 2 |
| BP | GO:0030534 | adult behavior | 0.014432882 | 3 |
| BP | GO:1904064 | positive regulation of cation transmembrane transport | 0.014432882 | 3 |
| BP | GO:0016447 | somatic recombination of immunoglobulin gene segments | 0.014566397 | 2 |
| BP | GO:0032653 | regulation of interleukin-10 production | 0.014566397 | 2 |
| BP | GO:0050885 | neuromuscular process controlling balance | 0.014566397 | 2 |
| BP | GO:0072132 | mesenchyme morphogenesis | 0.014566397 | 2 |
| BP | GO:1902373 | negative regulation of mRNA catabolic process | 0.014566397 | 2 |
| BP | GO:2000772 | regulation of cellular senescence | 0.014566397 | 2 |
| BP | GO:0000077 | DNA damage checkpoint | 0.01470121 | 3 |
| BP | GO:0002822 | regulation of adaptive immune response based on somatic recombination of immune receptors built from immunoglobulin superfamily domains | 0.01470121 | 3 |
| BP | GO:0007605 | sensory perception of sound | 0.01470121 | 3 |
| BP | GO:0072331 | signal transduction by p53 class mediator | 0.014743878 | 4 |
| BP | GO:0007517 | muscle organ development | 0.014901283 | 5 |
| BP | GO:0022898 | regulation of transmembrane transporter activity | 0.014928315 | 4 |
| BP | GO:0051250 | negative regulation of lymphocyte activation | 0.014972366 | 3 |
| BP | GO:0010823 | negative regulation of mitochondrion organization | 0.015103464 | 2 |
| BP | GO:0031638 | zymogen activation | 0.015103464 | 2 |
| BP | GO:0032715 | negative regulation of interleukin-6 production | 0.015103464 | 2 |
| BP | GO:0043331 | response to dsRNA | 0.015103464 | 2 |
| BP | GO:0045661 | regulation of myoblast differentiation | 0.015103464 | 2 |
| BP | GO:0050732 | negative regulation of peptidyl-tyrosine phosphorylation | 0.015103464 | 2 |
| BP | GO:0060563 | neuroepithelial cell differentiation | 0.015103464 | 2 |
| BP | GO:0070228 | regulation of lymphocyte apoptotic process | 0.015103464 | 2 |
| BP | GO:1901185 | negative regulation of ERBB signaling pathway | 0.015103464 | 2 |
| BP | GO:1905517 | macrophage migration | 0.015103464 | 2 |
| BP | GO:0051494 | negative regulation of cytoskeleton organization | 0.015523173 | 3 |
| BP | GO:0002763 | positive regulation of myeloid leukocyte differentiation | 0.015648977 | 2 |
| BP | GO:0032210 | regulation of telomere maintenance via telomerase | 0.015648977 | 2 |
| BP | GO:0032663 | regulation of interleukin-2 production | 0.015648977 | 2 |
| BP | GO:0061005 | cell differentiation involved in kidney development | 0.015648977 | 2 |
| BP | GO:0097345 | mitochondrial outer membrane permeabilization | 0.015648977 | 2 |
| BP | GO:0002706 | regulation of lymphocyte mediated immunity | 0.015802832 | 3 |
| BP | GO:0048592 | eye morphogenesis | 0.015802832 | 3 |
| BP | GO:0006383 | transcription by RNA polymerase III | 0.016202873 | 2 |
| BP | GO:0016233 | telomere capping | 0.016202873 | 2 |
| BP | GO:0042304 | regulation of fatty acid biosynthetic process | 0.016202873 | 2 |
| BP | GO:0045620 | negative regulation of lymphocyte differentiation | 0.016202873 | 2 |
| BP | GO:0048016 | inositol phosphate-mediated signaling | 0.016202873 | 2 |
| BP | GO:0070830 | bicellular tight junction assembly | 0.016202873 | 2 |
| BP | GO:0043588 | skin development | 0.016223343 | 5 |
| BP | GO:0055088 | lipid homeostasis | 0.016370674 | 3 |
| BP | GO:0001912 | positive regulation of leukocyte mediated cytotoxicity | 0.016765086 | 2 |
| BP | GO:0002381 | immunoglobulin production involved in immunoglobulin mediated immune response | 0.016765086 | 2 |
| BP | GO:0120192 | tight junction assembly | 0.016765086 | 2 |
| BP | GO:0001822 | kidney development | 0.016853548 | 4 |
| BP | GO:0033344 | cholesterol efflux | 0.017335553 | 2 |
| BP | GO:0043525 | positive regulation of neuron apoptotic process | 0.017335553 | 2 |
| BP | GO:0060043 | regulation of cardiac muscle cell proliferation | 0.017335553 | 2 |
| BP | GO:0060760 | positive regulation of response to cytokine stimulus | 0.017335553 | 2 |
| BP | GO:0016573 | histone acetylation | 0.017840134 | 3 |
| BP | GO:0034767 | positive regulation of ion transmembrane transport | 0.017840134 | 3 |
| BP | GO:0016579 | protein deubiquitination | 0.017871983 | 4 |
| BP | GO:0030520 | intracellular estrogen receptor signaling pathway | 0.01791421 | 2 |
| BP | GO:0098930 | axonal transport | 0.01791421 | 2 |
| BP | GO:1903749 | positive regulation of establishment of protein localization to mitochondrion | 0.01791421 | 2 |
| BP | GO:0019827 | stem cell population maintenance | 0.018142592 | 3 |
| BP | GO:0031570 | DNA integrity checkpoint | 0.018142592 | 3 |
| BP | GO:0016482 | cytosolic transport | 0.01844791 | 3 |
| BP | GO:0052126 | movement in host environment | 0.01844791 | 3 |
| BP | GO:0001836 | release of cytochrome c from mitochondria | 0.018500995 | 2 |
| BP | GO:0030521 | androgen receptor signaling pathway | 0.018500995 | 2 |
| BP | GO:0031102 | neuron projection regeneration | 0.018500995 | 2 |
| BP | GO:0042130 | negative regulation of T cell proliferation | 0.018500995 | 2 |
| BP | GO:0097755 | positive regulation of blood vessel diameter | 0.018500995 | 2 |
| BP | GO:1902369 | negative regulation of RNA catabolic process | 0.018500995 | 2 |
| BP | GO:0006665 | sphingolipid metabolic process | 0.01875609 | 3 |
| BP | GO:0098727 | maintenance of cell number | 0.01875609 | 3 |
| BP | GO:0006605 | protein targeting | 0.018763469 | 5 |
| BP | GO:0048562 | embryonic organ morphogenesis | 0.01892817 | 4 |
| BP | GO:2000241 | regulation of reproductive process | 0.019067133 | 3 |
| BP | GO:0032507 | maintenance of protein location in cell | 0.019095844 | 2 |
| BP | GO:0051893 | regulation of focal adhesion assembly | 0.019095844 | 2 |
| BP | GO:0090109 | regulation of cell-substrate junction assembly | 0.019095844 | 2 |
| BP | GO:0090342 | regulation of cell aging | 0.019095844 | 2 |
| BP | GO:0120193 | tight junction organization | 0.019095844 | 2 |
| BP | GO:0150116 | regulation of cell-substrate junction organization | 0.019095844 | 2 |
| BP | GO:1902110 | positive regulation of mitochondrial membrane permeability involved in apoptotic process | 0.019095844 | 2 |
| BP | GO:0010970 | transport along microtubule | 0.019381042 | 3 |
| BP | GO:0018393 | internal peptidyl-lysine acetylation | 0.019381042 | 3 |
| BP | GO:1902652 | secondary alcohol metabolic process | 0.019381042 | 3 |
| BP | GO:0000723 | telomere maintenance | 0.019697818 | 3 |
| BP | GO:0030833 | regulation of actin filament polymerization | 0.019697818 | 3 |
| BP | GO:0032233 | positive regulation of actin filament bundle assembly | 0.019698694 | 2 |
| BP | GO:0032370 | positive regulation of lipid transport | 0.019698694 | 2 |
| BP | GO:0034113 | heterotypic cell-cell adhesion | 0.019698694 | 2 |
| BP | GO:0042490 | mechanoreceptor differentiation | 0.019698694 | 2 |
| BP | GO:0046513 | ceramide biosynthetic process | 0.019698694 | 2 |
| BP | GO:0090287 | regulation of cellular response to growth factor stimulus | 0.019800554 | 4 |
| BP | GO:0072001 | renal system development | 0.020022485 | 4 |
| BP | GO:0002562 | somatic diversification of immune receptors via germline recombination within a single locus | 0.020309484 | 2 |
| BP | GO:0002753 | cytoplasmic pattern recognition receptor signaling pathway | 0.020309484 | 2 |
| BP | GO:0010830 | regulation of myotube differentiation | 0.020309484 | 2 |
| BP | GO:0016444 | somatic cell DNA recombination | 0.020309484 | 2 |
| BP | GO:0032623 | interleukin-2 production | 0.020309484 | 2 |
| BP | GO:0032835 | glomerulus development | 0.020309484 | 2 |
| BP | GO:0032922 | circadian regulation of gene expression | 0.020309484 | 2 |
| BP | GO:0045453 | bone resorption | 0.020309484 | 2 |
| BP | GO:0051898 | negative regulation of protein kinase B signaling | 0.020309484 | 2 |
| BP | GO:1902686 | mitochondrial outer membrane permeabilization involved in programmed cell death | 0.020309484 | 2 |
| BP | GO:1904356 | regulation of telomere maintenance via telomere lengthening | 0.020309484 | 2 |
| BP | GO:1905268 | negative regulation of chromatin organization | 0.020309484 | 2 |
| BP | GO:0001818 | negative regulation of cytokine production | 0.020697524 | 4 |
| BP | GO:0032371 | regulation of sterol transport | 0.020928152 | 2 |
| BP | GO:0034394 | protein localization to cell surface | 0.020928152 | 2 |
| BP | GO:0046530 | photoreceptor cell differentiation | 0.020928152 | 2 |
| BP | GO:2000401 | regulation of lymphocyte migration | 0.020928152 | 2 |
| BP | GO:0090288 | negative regulation of cellular response to growth factor stimulus | 0.02099361 | 3 |
| BP | GO:0070646 | protein modification by small protein removal | 0.021386489 | 4 |
| BP | GO:0016445 | somatic diversification of immunoglobulins | 0.021554637 | 2 |
| BP | GO:0030032 | lamellipodium assembly | 0.021554637 | 2 |
| BP | GO:0035773 | insulin secretion involved in cellular response to glucose stimulus | 0.021554637 | 2 |
| BP | GO:0035794 | positive regulation of mitochondrial membrane permeability | 0.021554637 | 2 |
| BP | GO:0043297 | apical junction assembly | 0.021554637 | 2 |
| BP | GO:0046503 | glycerolipid catabolic process | 0.021554637 | 2 |
| BP | GO:0048247 | lymphocyte chemotaxis | 0.021554637 | 2 |
| BP | GO:2000378 | negative regulation of reactive oxygen species metabolic process | 0.021554637 | 2 |
| BP | GO:1990138 | neuron projection extension | 0.021658733 | 3 |
| BP | GO:0010769 | regulation of cell morphogenesis involved in differentiation | 0.02185357 | 4 |
| BP | GO:0010469 | regulation of signaling receptor activity | 0.021995604 | 3 |
| BP | GO:0018394 | peptidyl-lysine acetylation | 0.021995604 | 3 |
| BP | GO:0006940 | regulation of smooth muscle contraction | 0.022188876 | 2 |
| BP | GO:0014823 | response to activity | 0.022188876 | 2 |
| BP | GO:0045739 | positive regulation of DNA repair | 0.022188876 | 2 |
| BP | GO:0003205 | cardiac chamber development | 0.022677968 | 3 |
| BP | GO:0006687 | glycosphingolipid metabolic process | 0.02283081 | 2 |
| BP | GO:0015800 | acidic amino acid transport | 0.02283081 | 2 |
| BP | GO:1902108 | regulation of mitochondrial membrane permeability involved in apoptotic process | 0.02283081 | 2 |
| BP | GO:1905710 | positive regulation of membrane permeability | 0.02283081 | 2 |
| BP | GO:0016525 | negative regulation of angiogenesis | 0.023371827 | 3 |
| BP | GO:0060038 | cardiac muscle cell proliferation | 0.023480377 | 2 |
| BP | GO:2001252 | positive regulation of chromosome organization | 0.023723067 | 3 |
| BP | GO:0008544 | epidermis development | 0.024012783 | 5 |
| BP | GO:0032200 | telomere organization | 0.024077182 | 3 |
| BP | GO:2000181 | negative regulation of blood vessel morphogenesis | 0.024077182 | 3 |
| BP | GO:0050891 | multicellular organismal water homeostasis | 0.024137517 | 2 |
| BP | GO:0050918 | positive chemotaxis | 0.024137517 | 2 |
| BP | GO:0051965 | positive regulation of synapse assembly | 0.024137517 | 2 |
| BP | GO:0006986 | response to unfolded protein | 0.02443417 | 3 |
| BP | GO:0022406 | membrane docking | 0.025156766 | 3 |
| BP | GO:0034121 | regulation of toll-like receptor signaling pathway | 0.025474279 | 2 |
| BP | GO:0072091 | regulation of stem cell proliferation | 0.025474279 | 2 |
| BP | GO:0006611 | protein export from nucleus | 0.025522373 | 3 |
| BP | GO:0008064 | regulation of actin polymerization or depolymerization | 0.025522373 | 3 |
| BP | GO:0071347 | cellular response to interleukin-1 | 0.025522373 | 3 |
| BP | GO:0030705 | cytoskeleton-dependent intracellular transport | 0.02589085 | 3 |
| BP | GO:0030832 | regulation of actin filament length | 0.02589085 | 3 |
| BP | GO:2001257 | regulation of cation channel activity | 0.02589085 | 3 |
| BP | GO:0033692 | cellular polysaccharide biosynthetic process | 0.026153781 | 2 |
| BP | GO:0050795 | regulation of behavior | 0.026153781 | 2 |
| BP | GO:0010498 | proteasomal protein catabolic process | 0.026646508 | 5 |
| BP | GO:0032024 | positive regulation of insulin secretion | 0.026840619 | 2 |
| BP | GO:0032272 | negative regulation of protein polymerization | 0.026840619 | 2 |
| BP | GO:0051155 | positive regulation of striated muscle cell differentiation | 0.026840619 | 2 |
| BP | GO:0021953 | central nervous system neuron differentiation | 0.027013504 | 3 |
| BP | GO:0061136 | regulation of proteasomal protein catabolic process | 0.027013504 | 3 |
| BP | GO:2000045 | regulation of G1/S transition of mitotic cell cycle | 0.027393458 | 3 |
| BP | GO:0030239 | myofibril assembly | 0.027534734 | 2 |
| BP | GO:0031100 | animal organ regeneration | 0.027534734 | 2 |
| BP | GO:0061844 | antimicrobial humoral immune response mediated by antimicrobial peptide | 0.027534734 | 2 |
| BP | GO:1903311 | regulation of mRNA metabolic process | 0.027678028 | 4 |
| BP | GO:0007369 | gastrulation | 0.027776278 | 3 |
| BP | GO:0030308 | negative regulation of cell growth | 0.028161963 | 3 |
| BP | GO:0005977 | glycogen metabolic process | 0.028236067 | 2 |
| BP | GO:0009064 | glutamine family amino acid metabolic process | 0.028236067 | 2 |
| BP | GO:0030104 | water homeostasis | 0.028236067 | 2 |
| BP | GO:0043507 | positive regulation of JUN kinase activity | 0.028236067 | 2 |
| BP | GO:0051145 | smooth muscle cell differentiation | 0.028236067 | 2 |
| BP | GO:0060998 | regulation of dendritic spine development | 0.028236067 | 2 |
| BP | GO:1900006 | positive regulation of dendrite development | 0.028236067 | 2 |
| BP | GO:0099111 | microtubule-based transport | 0.028550512 | 3 |
| BP | GO:1901343 | negative regulation of vasculature development | 0.028550512 | 3 |
| BP | GO:0001960 | negative regulation of cytokine-mediated signaling pathway | 0.028944561 | 2 |
| BP | GO:0002200 | somatic diversification of immune receptors | 0.028944561 | 2 |
| BP | GO:0003281 | ventricular septum development | 0.028944561 | 2 |
| BP | GO:0006073 | cellular glucan metabolic process | 0.028944561 | 2 |
| BP | GO:0010611 | regulation of cardiac muscle hypertrophy | 0.028944561 | 2 |
| BP | GO:0044042 | glucan metabolic process | 0.028944561 | 2 |
| BP | GO:0046173 | polyol biosynthetic process | 0.028944561 | 2 |
| CC | GO:0045121 | membrane raft | 5.13E-15 | 16 |
| CC | GO:0098857 | membrane microdomain | 5.39E-15 | 16 |
| CC | GO:0098589 | membrane region | 9.63E-15 | 16 |
| CC | GO:0005942 | phosphatidylinositol 3-kinase complex | 2.75E-08 | 5 |
| CC | GO:0005769 | early endosome | 2.39E-07 | 10 |
| CC | GO:0005925 | focal adhesion | 9.06E-07 | 10 |
| CC | GO:0030055 | cell-substrate junction | 1.06E-06 | 10 |
| CC | GO:0031983 | vesicle lumen | 1.37E-06 | 9 |
| CC | GO:0044853 | plasma membrane raft | 1.72E-06 | 6 |
| CC | GO:0043025 | neuronal cell body | 5.63E-06 | 10 |
| CC | GO:0005901 | caveola | 7.13E-06 | 5 |
| CC | GO:0031143 | pseudopodium | 2.36E-05 | 3 |
| CC | GO:0009897 | external side of plasma membrane | 4.86E-05 | 8 |
| CC | GO:0042629 | mast cell granule | 5.27E-05 | 3 |
| CC | GO:0034774 | secretory granule lumen | 9.71E-05 | 7 |
| CC | GO:0060205 | cytoplasmic vesicle lumen | 0.000104879 | 7 |
| CC | GO:0005790 | smooth endoplasmic reticulum | 0.000199033 | 3 |
| CC | GO:0009925 | basal plasma membrane | 0.000199033 | 3 |
| CC | GO:0031904 | endosome lumen | 0.000199033 | 3 |
| CC | GO:0061695 | transferase complex, transferring phosphorus-containing groups | 0.000227793 | 6 |
| CC | GO:0031091 | platelet alpha granule | 0.000245907 | 4 |
| CC | GO:0005775 | vacuolar lumen | 0.000277088 | 5 |
| CC | GO:0016607 | nuclear speck | 0.000356634 | 7 |
| CC | GO:0019898 | extrinsic component of membrane | 0.00045615 | 6 |
| CC | GO:0030139 | endocytic vesicle | 0.000525274 | 6 |
| CC | GO:0045178 | basal part of cell | 0.000665097 | 3 |
| CC | GO:0101002 | ficolin-1-rich granule | 0.000794749 | 4 |
| CC | GO:1904813 | ficolin-1-rich granule lumen | 0.000794749 | 4 |
| CC | GO:0005635 | nuclear envelope | 0.000898322 | 7 |
| CC | GO:0031093 | platelet alpha granule lumen | 0.001472721 | 3 |
| CC | GO:0000790 | nuclear chromatin | 0.001602164 | 6 |
| CC | GO:0005770 | late endosome | 0.001663772 | 5 |
| CC | GO:0043197 | dendritic spine | 0.001990218 | 4 |
| CC | GO:0071682 | endocytic vesicle lumen | 0.002016734 | 2 |
| CC | GO:0044309 | neuron spine | 0.002082848 | 4 |
| CC | GO:0090575 | RNA polymerase II transcription regulator complex | 0.002327397 | 4 |
| CC | GO:0062023 | collagen-containing extracellular matrix | 0.002356245 | 6 |
| CC | GO:0030426 | growth cone | 0.002591073 | 4 |
| CC | GO:0150034 | distal axon | 0.002652574 | 5 |
| CC | GO:0005911 | cell-cell junction | 0.002659194 | 6 |
| CC | GO:0120111 | neuron projection cytoplasm | 0.002813202 | 3 |
| CC | GO:0030427 | site of polarized growth | 0.002874607 | 4 |
| CC | GO:0097386 | glial cell projection | 0.002904409 | 2 |
| CC | GO:0005741 | mitochondrial outer membrane | 0.002993743 | 4 |
| CC | GO:0031965 | nuclear membrane | 0.00312085 | 5 |
| CC | GO:0001891 | phagocytic cup | 0.00366985 | 2 |
| CC | GO:0005788 | endoplasmic reticulum lumen | 0.003748167 | 5 |
| CC | GO:0043202 | lysosomal lumen | 0.003981285 | 3 |
| CC | GO:0031968 | organelle outer membrane | 0.004615057 | 4 |
| CC | GO:0019867 | outer membrane | 0.004779127 | 4 |
| CC | GO:0032838 | plasma membrane bounded cell projection cytoplasm | 0.005206396 | 4 |
| CC | GO:0010008 | endosome membrane | 0.005289231 | 6 |
| CC | GO:0005819 | spindle | 0.006097533 | 5 |
| CC | GO:0098978 | glutamatergic synapse | 0.006244449 | 5 |
| CC | GO:0001772 | immunological synapse | 0.006460579 | 2 |
| CC | GO:0034399 | nuclear periphery | 0.009671498 | 3 |
| CC | GO:0099568 | cytoplasmic region | 0.009973809 | 4 |
| CC | GO:0043209 | myelin sheath | 0.011727952 | 2 |
| CC | GO:0043195 | terminal bouton | 0.012660896 | 2 |
| CC | GO:0031901 | early endosome membrane | 0.013678005 | 3 |
| MF | GO:0004713 | protein tyrosine kinase activity | 5.72E-22 | 17 |
| MF | GO:0019902 | phosphatase binding | 1.85E-16 | 15 |
| MF | GO:0043560 | insulin receptor substrate binding | 2.36E-15 | 7 |
| MF | GO:0004714 | transmembrane receptor protein tyrosine kinase activity | 2.36E-14 | 10 |
| MF | GO:0019199 | transmembrane receptor protein kinase activity | 3.01E-13 | 10 |
| MF | GO:0019903 | protein phosphatase binding | 3.78E-12 | 11 |
| MF | GO:0051219 | phosphoprotein binding | 2.10E-11 | 9 |
| MF | GO:0001784 | phosphotyrosine residue binding | 1.22E-10 | 7 |
| MF | GO:0004674 | protein serine/threonine kinase activity | 6.50E-10 | 14 |
| MF | GO:0045309 | protein phosphorylated amino acid binding | 7.36E-10 | 7 |
| MF | GO:0005126 | cytokine receptor binding | 7.92E-09 | 11 |
| MF | GO:0004712 | protein serine/threonine/tyrosine kinase activity | 1.17E-08 | 6 |
| MF | GO:0004715 | non-membrane spanning protein tyrosine kinase activity | 1.78E-08 | 6 |
| MF | GO:0004879 | nuclear receptor activity | 2.03E-08 | 6 |
| MF | GO:0098531 | ligand-activated transcription factor activity | 2.03E-08 | 6 |
| MF | GO:0051427 | hormone receptor binding | 2.72E-08 | 9 |
| MF | GO:0016303 | 1-phosphatidylinositol-3-kinase activity | 3.64E-08 | 4 |
| MF | GO:0019838 | growth factor binding | 4.14E-08 | 8 |
| MF | GO:0035004 | phosphatidylinositol 3-kinase activity | 5.71E-08 | 4 |
| MF | GO:0097110 | scaffold protein binding | 8.23E-08 | 6 |
| MF | GO:0004707 | MAP kinase activity | 1.72E-07 | 4 |
| MF | GO:0004708 | MAP kinase kinase activity | 3.11E-07 | 4 |
| MF | GO:0016307 | phosphatidylinositol phosphate kinase activity | 3.11E-07 | 4 |
| MF | GO:0052742 | phosphatidylinositol kinase activity | 3.11E-07 | 4 |
| MF | GO:0044389 | ubiquitin-like protein ligase binding | 2.02E-06 | 9 |
| MF | GO:0003707 | steroid hormone receptor activity | 2.04E-06 | 5 |
| MF | GO:0043548 | phosphatidylinositol 3-kinase binding | 4.50E-06 | 4 |
| MF | GO:0031072 | heat shock protein binding | 5.33E-06 | 6 |
| MF | GO:0008022 | protein C-terminus binding | 6.05E-06 | 7 |
| MF | GO:0061629 | RNA polymerase II-specific DNA-binding transcription factor binding | 8.53E-06 | 8 |
| MF | GO:0042169 | SH2 domain binding | 9.50E-06 | 4 |
| MF | GO:0031625 | ubiquitin protein ligase binding | 1.19E-05 | 8 |
| MF | GO:0051879 | Hsp90 protein binding | 1.61E-05 | 4 |
| MF | GO:0035257 | nuclear hormone receptor binding | 2.16E-05 | 6 |
| MF | GO:0032813 | tumor necrosis factor receptor superfamily binding | 2.56E-05 | 4 |
| MF | GO:0042562 | hormone binding | 3.90E-05 | 5 |
| MF | GO:0140297 | DNA-binding transcription factor binding | 4.66E-05 | 8 |
| MF | GO:0005158 | insulin receptor binding | 7.25E-05 | 3 |
| MF | GO:0051019 | mitogen-activated protein kinase binding | 0.000121168 | 3 |
| MF | GO:0070491 | repressing transcription factor binding | 0.000142112 | 4 |
| MF | GO:0070851 | growth factor receptor binding | 0.000142551 | 5 |
| MF | GO:0020037 | heme binding | 0.000147611 | 5 |
| MF | GO:0046906 | tetrapyrrole binding | 0.000206122 | 5 |
| MF | GO:0005164 | tumor necrosis factor receptor binding | 0.000206708 | 3 |
| MF | GO:0001228 | DNA-binding transcription activator activity, RNA polymerase II-specific | 0.000219316 | 8 |
| MF | GO:0001216 | DNA-binding transcription activator activity | 0.000222724 | 8 |
| MF | GO:0051721 | protein phosphatase 2A binding | 0.000227484 | 3 |
| MF | GO:0097718 | disordered domain specific binding | 0.000249567 | 3 |
| MF | GO:0001103 | RNA polymerase II repressing transcription factor binding | 0.00029779 | 3 |
| MF | GO:0005496 | steroid binding | 0.000435459 | 4 |
| MF | GO:0140296 | general transcription initiation factor binding | 0.000512685 | 3 |
| MF | GO:0048020 | CCR chemokine receptor binding | 0.000549673 | 3 |
| MF | GO:0005165 | neurotrophin receptor binding | 0.000604733 | 2 |
| MF | GO:0001664 | G protein-coupled receptor binding | 0.000609951 | 6 |
| MF | GO:0048407 | platelet-derived growth factor binding | 0.00073734 | 2 |
| MF | GO:0050661 | NADP binding | 0.000961074 | 3 |
| MF | GO:0016653 | oxidoreductase activity, acting on NAD(P)H, heme protein as acceptor | 0.001040657 | 2 |
| MF | GO:0019207 | kinase regulator activity | 0.001045466 | 5 |
| MF | GO:0002020 | protease binding | 0.00133109 | 4 |
| MF | GO:0005161 | platelet-derived growth factor receptor binding | 0.001394157 | 2 |
| MF | GO:0097153 | cysteine-type endopeptidase activity involved in apoptotic process | 0.001394157 | 2 |
| MF | GO:0005178 | integrin binding | 0.001490943 | 4 |
| MF | GO:0010181 | FMN binding | 0.001589493 | 2 |
| MF | GO:0043274 | phospholipase binding | 0.001589493 | 2 |
| MF | GO:0005123 | death receptor binding | 0.001797098 | 2 |
| MF | GO:0034185 | apolipoprotein binding | 0.001797098 | 2 |
| MF | GO:0002039 | p53 binding | 0.001917413 | 3 |
| MF | GO:0042379 | chemokine receptor binding | 0.001917413 | 3 |
| MF | GO:0001091 | RNA polymerase II general transcription initiation factor binding | 0.002492608 | 2 |
| MF | GO:0050839 | cell adhesion molecule binding | 0.002517244 | 7 |
| MF | GO:0051428 | peptide hormone receptor binding | 0.002748376 | 2 |
| MF | GO:0016705 | oxidoreductase activity, acting on paired donors, with incorporation or reduction of molecular oxygen | 0.002934935 | 4 |
| MF | GO:0004252 | serine-type endopeptidase activity | 0.003001852 | 4 |
| MF | GO:0001223 | transcription coactivator binding | 0.00329527 | 2 |
| MF | GO:0008201 | heparin binding | 0.003651737 | 4 |
| MF | GO:0019209 | kinase activator activity | 0.004067068 | 3 |
| MF | GO:0004866 | endopeptidase inhibitor activity | 0.004134482 | 4 |
| MF | GO:0004709 | MAP kinase kinase kinase activity | 0.004202682 | 2 |
| MF | GO:0046875 | ephrin receptor binding | 0.00452802 | 2 |
| MF | GO:0008236 | serine-type peptidase activity | 0.004750252 | 4 |
| MF | GO:0030414 | peptidase inhibitor activity | 0.004750252 | 4 |
| MF | GO:0061135 | endopeptidase regulator activity | 0.004750252 | 4 |
| MF | GO:0003785 | actin monomer binding | 0.004864648 | 2 |
| MF | GO:0035258 | steroid hormone receptor binding | 0.004912149 | 3 |
| MF | GO:1990782 | protein tyrosine kinase binding | 0.0050625 | 3 |
| MF | GO:0004175 | endopeptidase activity | 0.005109983 | 6 |
| MF | GO:0016825 | hydrolase activity, acting on acid phosphorus-nitrogen bonds | 0.005128458 | 4 |
| MF | GO:0017171 | serine hydrolase activity | 0.005128458 | 4 |
| MF | GO:0035035 | histone acetyltransferase binding | 0.005212479 | 2 |
| MF | GO:0031406 | carboxylic acid binding | 0.005838065 | 4 |
| MF | GO:0004497 | monooxygenase activity | 0.006022933 | 3 |
| MF | GO:0042393 | histone binding | 0.006271607 | 4 |
| MF | GO:0051087 | chaperone binding | 0.006541239 | 3 |
| MF | GO:0005516 | calmodulin binding | 0.006610486 | 4 |
| MF | GO:0005504 | fatty acid binding | 0.007116699 | 2 |
| MF | GO:0043177 | organic acid binding | 0.007201955 | 4 |
| MF | GO:0001530 | lipopolysaccharide binding | 0.007529966 | 2 |
| MF | GO:0047485 | protein N-terminus binding | 0.007851628 | 3 |
| MF | GO:0016922 | nuclear receptor binding | 0.008050533 | 3 |
| MF | GO:0000287 | magnesium ion binding | 0.008219436 | 4 |
| MF | GO:0042826 | histone deacetylase binding | 0.008252396 | 3 |
| MF | GO:0061134 | peptidase regulator activity | 0.009041655 | 4 |
| MF | GO:0016709 | oxidoreductase activity, acting on paired donors, with incorporation or reduction of molecular oxygen, NAD(P)H as one donor, and incorporation of one atom of oxygen | 0.009288346 | 2 |
| MF | GO:0005539 | glycosaminoglycan binding | 0.010528403 | 4 |
| MF | GO:0030331 | estrogen receptor binding | 0.010715424 | 2 |
| MF | GO:0001221 | transcription cofactor binding | 0.011211314 | 2 |
| MF | GO:0048156 | tau protein binding | 0.012232914 | 2 |
| MF | GO:0004857 | enzyme inhibitor activity | 0.012936282 | 5 |
| MF | GO:0043021 | ribonucleoprotein complex binding | 0.013459066 | 3 |
| MF | GO:1901681 | sulfur compound binding | 0.01414599 | 4 |
| MF | GO:0017046 | peptide hormone binding | 0.014393201 | 2 |
| MF | GO:0070888 | E-box binding | 0.014957214 | 2 |
| MF | GO:0005080 | protein kinase C binding | 0.016705488 | 2 |

| Table S3. KEGG enrichment analysis results (*P*<0.05). | | | |
| --- | --- | --- | --- |
| ID | Description | *p*-value | Count |
| hsa05205 | Proteoglycans in cancer | 4.31E-28 | 28 |
| hsa01521 | EGFR tyrosine kinase inhibitor resistance | 2.07E-27 | 21 |
| hsa05235 | PD-L1 expression and PD-1 checkpoint pathway in cancer | 3.43E-26 | 21 |
| hsa01522 | Endocrine resistance | 3.18E-25 | 21 |
| hsa05230 | Central carbon metabolism in cancer | 5.48E-25 | 19 |
| hsa04151 | PI3K-Akt signaling pathway | 6.20E-24 | 30 |
| hsa05215 | Prostate cancer | 1.19E-23 | 20 |
| hsa04933 | AGE-RAGE signaling pathway in diabetic complications | 2.31E-23 | 20 |
| hsa04625 | C-type lectin receptor signaling pathway | 5.37E-23 | 20 |
| hsa05167 | Kaposi sarcoma-associated herpesvirus infection | 7.61E-23 | 24 |
| hsa04066 | HIF-1 signaling pathway | 5.55E-21 | 19 |
| hsa04014 | Ras signaling pathway | 5.90E-21 | 24 |
| hsa05417 | Lipid and atherosclerosis | 2.15E-20 | 23 |
| hsa05161 | Hepatitis B | 2.25E-20 | 21 |
| hsa04917 | Prolactin signaling pathway | 9.35E-20 | 16 |
| hsa05218 | Melanoma | 1.53E-19 | 16 |
| hsa05214 | Glioma | 3.13E-19 | 16 |
| hsa05212 | Pancreatic cancer | 3.94E-19 | 16 |
| hsa05163 | Human cytomegalovirus infection | 1.24E-18 | 22 |
| hsa04072 | Phospholipase D signaling pathway | 2.36E-18 | 19 |
| hsa04926 | Relaxin signaling pathway | 4.54E-18 | 18 |
| hsa04370 | VEGF signaling pathway | 1.30E-17 | 14 |
| hsa04915 | Estrogen signaling pathway | 1.58E-17 | 18 |
| hsa04722 | Neurotrophin signaling pathway | 2.89E-17 | 17 |
| hsa04935 | Growth hormone synthesis, secretion and action | 2.89E-17 | 17 |
| hsa05231 | Choline metabolism in cancer | 3.03E-17 | 16 |
| hsa04660 | T cell receptor signaling pathway | 8.19E-17 | 16 |
| hsa04012 | ErbB signaling pathway | 9.73E-17 | 15 |
| hsa04664 | Fc epsilon RI signaling pathway | 1.14E-16 | 14 |
| hsa05207 | Chemical carcinogenesis - receptor activation | 1.27E-16 | 20 |
| hsa04068 | FoxO signaling pathway | 1.55E-16 | 17 |
| hsa05160 | Hepatitis C | 1.67E-16 | 18 |
| hsa05223 | Non-small cell lung cancer | 2.69E-16 | 14 |
| hsa04668 | TNF signaling pathway | 2.80E-16 | 16 |
| hsa04010 | MAPK signaling pathway | 3.99E-16 | 22 |
| hsa05164 | Influenza A | 7.83E-16 | 18 |
| hsa04919 | Thyroid hormone signaling pathway | 9.99E-16 | 16 |
| hsa05206 | MicroRNAs in cancer | 1.22E-15 | 22 |
| hsa05142 | Chagas disease | 1.71E-15 | 15 |
| hsa05221 | Acute myeloid leukemia | 3.56E-15 | 13 |
| hsa05210 | Colorectal cancer | 3.76E-15 | 14 |
| hsa05211 | Renal cell carcinoma | 5.36E-15 | 13 |
| hsa04630 | JAK-STAT signaling pathway | 5.93E-15 | 17 |
| hsa05145 | Toxoplasmosis | 7.23E-15 | 15 |
| hsa04140 | Autophagy - animal | 1.20E-14 | 16 |
| hsa04510 | Focal adhesion | 1.39E-14 | 18 |
| hsa05213 | Endometrial cancer | 1.96E-14 | 12 |
| hsa05220 | Chronic myeloid leukemia | 2.03E-14 | 13 |
| hsa05224 | Breast cancer | 2.33E-14 | 16 |
| hsa04015 | Rap1 signaling pathway | 3.02E-14 | 18 |
| hsa05132 | Salmonella infection | 4.33E-14 | 19 |
| hsa04914 | Progesterone-mediated oocyte maturation | 4.47E-14 | 14 |
| hsa04380 | Osteoclast differentiation | 5.53E-14 | 15 |
| hsa04620 | Toll-like receptor signaling pathway | 5.90E-14 | 14 |
| hsa05165 | Human papillomavirus infection | 5.96E-14 | 21 |
| hsa04650 | Natural killer cell mediated cytotoxicity | 7.85E-14 | 15 |
| hsa05208 | Chemical carcinogenesis - reactive oxygen species | 8.63E-14 | 18 |
| hsa04210 | Apoptosis | 1.38E-13 | 15 |
| hsa04550 | Signaling pathways regulating pluripotency of stem cells | 2.92E-13 | 15 |
| hsa04071 | Sphingolipid signaling pathway | 4.01E-13 | 14 |
| hsa05170 | Human immunodeficiency virus 1 infection | 5.30E-13 | 17 |
| hsa05219 | Bladder cancer | 5.75E-13 | 10 |
| hsa04218 | Cellular senescence | 1.07E-12 | 15 |
| hsa05010 | Alzheimer disease | 1.10E-12 | 21 |
| hsa04062 | Chemokine signaling pathway | 1.57E-12 | 16 |
| hsa04930 | Type II diabetes mellitus | 2.03E-12 | 10 |
| hsa04659 | Th17 cell differentiation | 2.25E-12 | 13 |
| hsa04931 | Insulin resistance | 2.25E-12 | 13 |
| hsa05171 | Coronavirus disease - COVID-19 | 2.31E-12 | 17 |
| hsa05135 | Yersinia infection | 2.88E-12 | 14 |
| hsa05169 | Epstein-Barr virus infection | 3.45E-12 | 16 |
| hsa05418 | Fluid shear stress and atherosclerosis | 3.52E-12 | 14 |
| hsa05152 | Tuberculosis | 8.68E-12 | 15 |
| hsa05226 | Gastric cancer | 9.17E-12 | 14 |
| hsa01524 | Platinum drug resistance | 1.04E-11 | 11 |
| hsa04666 | Fc gamma R-mediated phagocytosis | 1.23E-11 | 12 |
| hsa04611 | Platelet activation | 1.36E-11 | 13 |
| hsa04662 | B cell receptor signaling pathway | 3.87E-11 | 11 |
| hsa05225 | Hepatocellular carcinoma | 4.72E-11 | 14 |
| hsa04213 | Longevity regulating pathway - multiple species | 4.83E-11 | 10 |
| hsa04910 | Insulin signaling pathway | 4.88E-11 | 13 |
| hsa05162 | Measles | 5.87E-11 | 13 |
| hsa04929 | GnRH secretion | 6.72E-11 | 10 |
| hsa04725 | Cholinergic synapse | 7.74E-11 | 12 |
| hsa04211 | Longevity regulating pathway | 9.67E-11 | 11 |
| hsa04912 | GnRH signaling pathway | 1.57E-10 | 11 |
| hsa05166 | Human T-cell leukemia virus 1 infection | 1.76E-10 | 15 |
| hsa04150 | mTOR signaling pathway | 2.53E-10 | 13 |
| hsa05140 | Leishmaniasis | 4.48E-10 | 10 |
| hsa05415 | Diabetic cardiomyopathy | 5.95E-10 | 14 |
| hsa05203 | Viral carcinogenesis | 6.34E-10 | 14 |
| hsa05131 | Shigellosis | 7.88E-10 | 15 |
| hsa04371 | Apelin signaling pathway | 8.87E-10 | 12 |
| hsa04613 | Neutrophil extracellular trap formation | 2.92E-09 | 13 |
| hsa04932 | Non-alcoholic fatty liver disease | 3.13E-09 | 12 |
| hsa04657 | IL-17 signaling pathway | 3.32E-09 | 10 |
| hsa04810 | Regulation of actin cytoskeleton | 1.55E-08 | 13 |
| hsa04540 | Gap junction | 3.07E-08 | 9 |
| hsa04658 | Th1 and Th2 cell differentiation | 4.54E-08 | 9 |
| hsa05130 | Pathogenic Escherichia coli infection | 4.70E-08 | 12 |
| hsa04920 | Adipocytokine signaling pathway | 7.02E-08 | 8 |
| hsa04750 | Inflammatory mediator regulation of TRP channels | 7.92E-08 | 9 |
| hsa05146 | Amoebiasis | 1.12E-07 | 9 |
| hsa05022 | Pathways of neurodegeneration - multiple diseases | 1.50E-07 | 17 |
| hsa05133 | Pertussis | 1.51E-07 | 8 |
| hsa04024 | cAMP signaling pathway | 1.67E-07 | 12 |
| hsa04360 | Axon guidance | 1.97E-07 | 11 |
| hsa04621 | NOD-like receptor signaling pathway | 2.20E-07 | 11 |
| hsa05020 | Prion disease | 2.21E-07 | 13 |
| hsa04923 | Regulation of lipolysis in adipocytes | 2.89E-07 | 7 |
| hsa04670 | Leukocyte transendothelial migration | 2.95E-07 | 9 |
| hsa04726 | Serotonergic synapse | 3.18E-07 | 9 |
| hsa04921 | Oxytocin signaling pathway | 3.85E-07 | 10 |
| hsa04960 | Aldosterone-regulated sodium reabsorption | 4.39E-07 | 6 |
| hsa05216 | Thyroid cancer | 4.39E-07 | 6 |
| hsa04152 | AMPK signaling pathway | 4.57E-07 | 9 |
| hsa05222 | Small cell lung cancer | 6.74E-07 | 8 |
| hsa04936 | Alcoholic liver disease | 1.90E-06 | 9 |
| hsa04020 | Calcium signaling pathway | 3.09E-06 | 11 |
| hsa04217 | Necroptosis | 4.85E-06 | 9 |
| hsa04730 | Long-term depression | 8.17E-06 | 6 |
| hsa04720 | Long-term potentiation | 1.56E-05 | 6 |
| hsa04916 | Melanogenesis | 1.61E-05 | 7 |
| hsa05120 | Epithelial cell signaling in Helicobacter pylori infection | 2.01E-05 | 6 |
| hsa04520 | Adherens junction | 2.18E-05 | 6 |
| hsa05168 | Herpes simplex virus 1 infection | 3.37E-05 | 14 |
| hsa04973 | Carbohydrate digestion and absorption | 3.60E-05 | 5 |
| hsa04114 | Oocyte meiosis | 8.60E-05 | 7 |
| hsa05017 | Spinocerebellar ataxia | 0.000149211 | 7 |
| hsa05321 | Inflammatory bowel disease | 0.000173271 | 5 |
| hsa04064 | NF-kappa B signaling pathway | 0.000187226 | 6 |
| hsa04928 | Parathyroid hormone synthesis, secretion and action | 0.000207834 | 6 |
| hsa05100 | Bacterial invasion of epithelial cells | 0.000384498 | 5 |
| hsa04022 | cGMP-PKG signaling pathway | 0.000387556 | 7 |
| hsa05144 | Malaria | 0.000694927 | 4 |
| hsa04270 | Vascular smooth muscle contraction | 0.000732323 | 6 |
| hsa05034 | Alcoholism | 0.000763402 | 7 |
| hsa05134 | Legionellosis | 0.001142221 | 4 |
| hsa04723 | Retrograde endocannabinoid signaling | 0.001229926 | 6 |
| hsa04261 | Adrenergic signaling in cardiomyocytes | 0.001318142 | 6 |
| hsa05416 | Viral myocarditis | 0.001384567 | 4 |
| hsa04215 | Apoptosis - multiple species | 0.002155772 | 3 |
| hsa04622 | RIG-I-like receptor signaling pathway | 0.00244997 | 4 |
| hsa00562 | Inositol phosphate metabolism | 0.002855866 | 4 |
| hsa04115 | p53 signaling pathway | 0.002855866 | 4 |
| hsa05202 | Transcriptional misregulation in cancer | 0.004546605 | 6 |
| hsa04070 | Phosphatidylinositol signaling system | 0.007862374 | 4 |
| hsa04913 | Ovarian steroidogenesis | 0.008110463 | 3 |
| hsa04934 | Cushing syndrome | 0.008364977 | 5 |
| hsa04922 | Glucagon signaling pathway | 0.011025103 | 4 |
| hsa00220 | Arginine biosynthesis | 0.013561279 | 2 |
| hsa05204 | Chemical carcinogenesis - DNA adducts | 0.018402005 | 3 |
| hsa04137 | Mitophagy - animal | 0.020590575 | 3 |
| hsa05012 | Parkinson disease | 0.020634571 | 6 |
| hsa04728 | Dopaminergic synapse | 0.022213495 | 4 |
| hsa04612 | Antigen processing and presentation | 0.025380281 | 3 |
| hsa04610 | Complement and coagulation cascades | 0.031662938 | 3 |
| hsa05143 | African trypanosomiasis | 0.036169393 | 2 |
| hsa05330 | Allograft rejection | 0.037982059 | 2 |
| hsa05340 | Primary immunodeficiency | 0.037982059 | 2 |
| hsa05323 | Rheumatoid arthritis | 0.039750548 | 3 |
| hsa04714 | Thermogenesis | 0.039956588 | 5 |
| hsa04350 | TGF-beta signaling pathway | 0.040828707 | 3 |
| hsa04713 | Circadian entrainment | 0.044151671 | 3 |
| hsa05332 | Graft-versus-host disease | 0.045568522 | 2 |
| hsa04940 | Type I diabetes mellitus | 0.047545855 | 2 |
| hsa04144 | Endocytosis | 0.052901211 | 5 |
| hsa00330 | Arginine and proline metabolism | 0.064430343 | 2 |
| hsa04724 | Glutamatergic synapse | 0.065415631 | 3 |
| hsa04961 | Endocrine and other factor-regulated calcium reabsorption | 0.068925889 | 2 |
| hsa05014 | Amyotrophic lateral sclerosis | 0.074661571 | 6 |
| hsa00590 | Arachidonic acid metabolism | 0.087872424 | 2 |
| hsa05016 | Huntington disease | 0.102719329 | 5 |
| hsa05031 | Amphetamine addiction | 0.108165681 | 2 |
| hsa00982 | Drug metabolism - cytochrome P450 | 0.116072982 | 2 |
| hsa03320 | PPAR signaling pathway | 0.124123833 | 2 |
| hsa04918 | Thyroid hormone synthesis | 0.124123833 | 2 |
| hsa00980 | Metabolism of xenobiotics by cytochrome P450 | 0.13230692 | 2 |
| hsa04530 | Tight junction | 0.158265877 | 3 |
| hsa04141 | Protein processing in endoplasmic reticulum | 0.16219127 | 3 |
| hsa04976 | Bile secretion | 0.163271971 | 2 |
| hsa03060 | Protein export | 0.171511146 | 1 |
| hsa04977 | Vitamin digestion and absorption | 0.178270976 | 1 |
| hsa05150 | Staphylococcus aureus infection | 0.183603583 | 2 |
| hsa05414 | Dilated cardiomyopathy | 0.183603583 | 2 |
| hsa04640 | Hematopoietic cell lineage | 0.192431456 | 2 |
| hsa04061 | Viral protein interaction with cytokine and cytokine receptor | 0.195387073 | 2 |
| hsa00591 | Linoleic acid metabolism | 0.211263784 | 1 |
| hsa01523 | Antifolate resistance | 0.21770404 | 1 |
| hsa05310 | Asthma | 0.2240925 | 1 |
| hsa04136 | Autophagy - other | 0.230429574 | 1 |
| hsa00760 | Nicotinate and nicotinamide metabolism | 0.249136534 | 1 |
| hsa04110 | Cell cycle | 0.273569549 | 2 |
| hsa04975 | Fat digestion and absorption | 0.296860083 | 1 |
| hsa04962 | Vasopressin-regulated water reabsorption | 0.302611383 | 1 |
| hsa04672 | Intestinal immune network for IgA production | 0.330680157 | 1 |
| hsa05030 | Cocaine addiction | 0.330680157 | 1 |
| hsa04979 | Cholesterol metabolism | 0.336158901 | 1 |
| hsa05110 | Vibrio cholerae infection | 0.336158901 | 1 |
| hsa04514 | Cell adhesion molecules | 0.342797621 | 2 |
| hsa05320 | Autoimmune thyroid disease | 0.352331497 | 1 |
| hsa04340 | Hedgehog signaling pathway | 0.36811592 | 1 |
| hsa00480 | Glutathione metabolism | 0.378427757 | 1 |
| hsa00140 | Steroid hormone biosynthesis | 0.393585513 | 1 |
| hsa04310 | Wnt signaling pathway | 0.395623132 | 2 |
| hsa04927 | Cortisol synthesis and secretion | 0.413230857 | 1 |
| hsa00010 | Glycolysis / Gluconeogenesis | 0.422817121 | 1 |
| hsa00830 | Retinol metabolism | 0.427552245 | 1 |
| hsa04924 | Renin secretion | 0.432249106 | 1 |
| hsa01230 | Biosynthesis of amino acids | 0.459643646 | 1 |
| hsa04971 | Gastric acid secretion | 0.464081063 | 1 |
| hsa00983 | Drug metabolism - other enzymes | 0.481474654 | 1 |
| hsa04146 | Peroxisome | 0.48996175 | 1 |
| hsa04742 | Taste transduction | 0.506527585 | 1 |
| hsa04911 | Insulin secretion | 0.506527585 | 1 |
| hsa04727 | GABAergic synapse | 0.518603119 | 1 |
| hsa05410 | Hypertrophic cardiomyopathy | 0.522563258 | 1 |
| hsa05032 | Morphine addiction | 0.526491309 | 1 |
| hsa04970 | Salivary secretion | 0.53425217 | 1 |
| hsa04080 | Neuroactive ligand-receptor interaction | 0.552880833 | 3 |
| hsa04925 | Aldosterone synthesis and secretion | 0.55311046 | 1 |
| hsa01200 | Carbon metabolism | 0.611774776 | 1 |
| hsa05322 | Systemic lupus erythematosus | 0.673858089 | 1 |
| hsa04120 | Ubiquitin mediated proteolysis | 0.689724258 | 1 |
| hsa04060 | Cytokine-cytokine receptor interaction | 0.69845523 | 2 |
| hsa04145 | Phagosome | 0.714495188 | 1 |
| hsa04390 | Hippo signaling pathway | 0.726139661 | 1 |
| hsa04740 | Olfactory transduction | 0.975174371 | 1 |
